# Design and synthesis of chromone-nitrogen mustard derivatives and evaluation of anti-breast cancer activity

**DOI:** 10.1080/14756366.2021.2018685

**Published:** 2021-12-27

**Authors:** Jianan Sun, Jiahui Mu, Shenglin Wang, Cai Jia, Dahong Li, Huiming Hua, Hao Cao

**Affiliations:** a Key Laboratory of Structure-Based Drug Design & Discovery, Ministry of Education, and School of Traditional Chinese Materia Medica, Shenyang Pharmaceutical University, Shenyang, PR China; b State Key Laboratory of Multiphase Complex Systems, Institute of Process Engineering, Chinese Academy of Sciences, Beijing, PR China; c School of Life Science and Biopharmaceutics, Shenyang Pharmaceutical University, Shenyang, PR China

**Keywords:** Chromone, nitrogen mustard, breast cancer, apoptosis

## Abstract

Chromone has emerged as one of the most important synthetic scaffolds for antitumor activity, which promotes the development of candidate drugs with better activity. In this study, a series of nitrogen mustard derivatives of chromone were designed and synthesised, in order to discover promising anti-breast tumour candidates. Almost all target derivatives showed antiproliferative activity against MCF-7 and MDA-MB-231 cell lines. In particular, methyl (*S*)-3-(4-(bis(2-chloroethyl)amino)phenyl)-2-(5-(((6-methoxy-4-oxo-4*H*-chromen-3-yl)methyl)amino)-5-oxopentanamido)propanoate showed the most potent antiproliferative activity with IC_50_ values of 1.83 and 1.90 μM, respectively, and it also exhibited certain selectivity between tumour cells and normal cells. Further mechanism exploration against MDA-MB-231 cells showed that it possibly induced G2/M phase arrest and apoptosis by generating intracellular ROS and activating DNA damage. In addition, it also inhibited MDA-MB-231 cells metastasis, invasion and adhesion. Overall, methyl (*S*)-3-(4-(bis(2-chloroethyl)amino)phenyl)-2-(5-(((6-methoxy-4-oxo-4*H*-chromen-3-yl)methyl)amino)-5-oxopentanamido)propanoate showed potent antitumor activities and relatively low side effects, and deserved further investigation.

## Graphical Abstract

**Figure:**
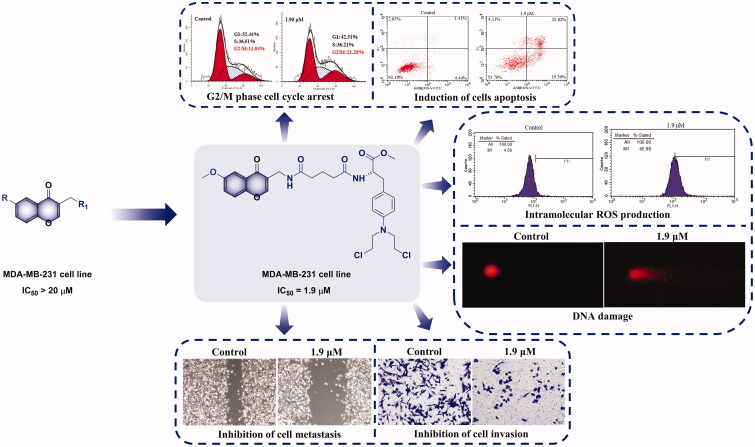

## 1. Introduction

As the most frequently diagnosed cancer, breast cancer is also the second most common cause of cancer mortality in female around the world^1–3^. It is regarded as a diverse and heterogeneous disease with different phenotypes, prognoses, and responses to treatment^4–6^. Statistics show that breast cancer accounts for 30% of female’s newly diagnosed cancer cases and 15% of female’s cancer deaths^7^^,^^8^. It is estimated that there are 1 million confirmed cases of breast cancer each year all over the world. Among them, approximately 170,000 (12–20%) cases belong to triple-negative breast cancers (TNBC)^9^^,^^10^. TNBC, which is characterised by lacking of oestrogen receptor (ER), progesterone receptor (PR), and human epidermal growth factor receptor 2 (HER2)^11^^,^^12^. Compared with hormone receptor-positive or HER2-positive diseases, TNBC has the characteristics of a highly aggressive clinical course, an earlier age of onset, greater metastatic potential, and poorer clinical outcomes^11^^,^^13–15^. TNBC has a high incidence rate among young premenopausal women^16^^,^^17^. Many efforts have been devoted to finding new drugs for the treatment of TNBC over the past decade, and chemotherapy is currently considered the most important therapeutic option for TNBC^17^.

**Figure 1.**
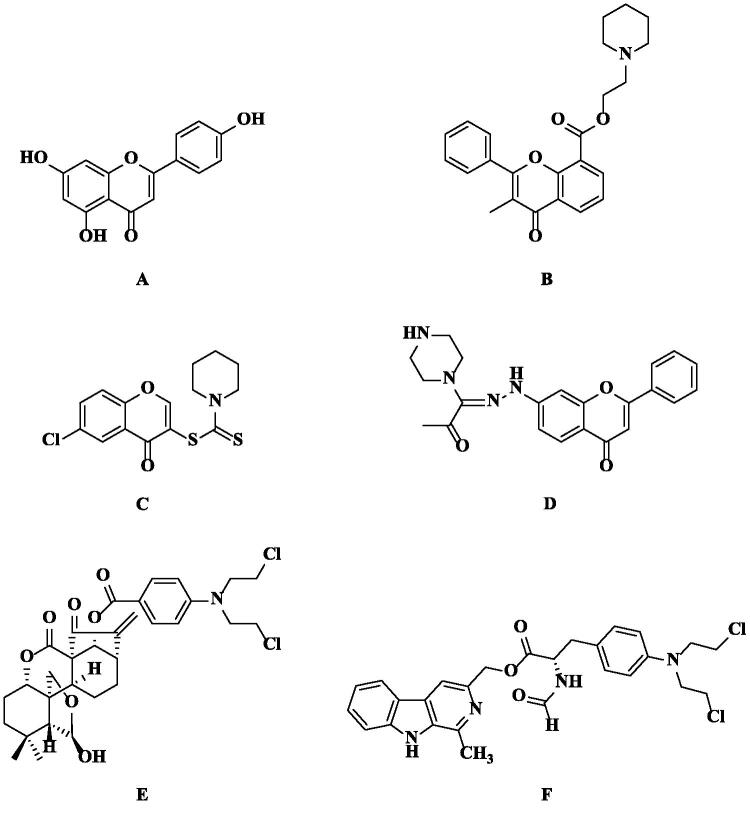
The chemical structures of reported chromone (**A**, **B**, **C** and **D**) and nitrogen mustard (**E** and **F**) derivatives.

From late 1930s to 2014, 77% of anti-tumour drugs approved worldwide are closely related to natural products, and the utilisation of natural products and/or their novel structures is still alive and well^18^^,^^19^. Chromones are a type of compounds with a benzo-*γ*-pyrone skeleton, which are widely distributed in nature^20^^,^^21^. It has been proved that chromones have various kinds of biological activities, including antitumor^22^, antimicrobial^23^^,^^24^, anti-HIV^25^, anti-inflammatory^26^, antioxidant^27^, wound healing^28^, and so on. In addition, chromones and its derivatives are also the most important heterocyclic compounds, playing an important role in the design and discovery of new physiologically/pharmacologically active compounds^20^^,^^29^, such as apigenin (4′,5,7-trihydroxyflavone, **A**, Figure 1), flavoxate (2-(1-piperidyl)ethyl 3-methyl-4-oxo-2-phenylchromene-8-carboxylate, **B**, Figure 1), etc.^30–32^. More importantly, chromone derivatives possess low mammalian toxicity and are present in large amounts in the diet of humans due to their origin in plants^21^. In terms of antitumor activity, chromones show activity against many kinds of tumour cells, and the antiproliferative mechanisms involve cytotoxicity, anti-metastasis, anti-angiogenesis, chemoprevention, immunomodulation, etc.^33–39^. To date, there are some chromone derivatives showing potent cytotoxic effect on breast cancer cells, such as **C** and **D** (Figure 1)^40^^,^^41^.

Nitrogen mustards, a class of DNA alkylating agents, mark the beginning of modern cancer chemotherapy and have been developed into many therapeutic compounds with broad antitumor activity^42^^,^^43^. They exert their cytotoxic effects through the formation of DNA interstrand cross-links^44^^,^^45^ and have been widely used in the treatment of various blood cancers and solid tumours^46^^,^^47^. In addition, nitrogen mustards are representative of dichloroethylamine alkylating agents. Aliphatic nitrogen mustard has the characteristics of sufficient therapeutic index but high reactivity and peripheral cytotoxicity. In contrast, aromatic nitrogen mustard is less electrophilic with low reactivity and can be administered orally. Nevertheless, this kind of nitrogen mustard derivatives still causes severe side effects and acquired drug resistance due to non-specific affinity to cancer cells^46^^,^^48^^,^^49^. To solve these problems, linking aromatic nitrogen mustards to natural products is a good way to reduce the side effects and improve activities, such as **E** and **F** (Figure 1)^50^^,^^51^. Thus, we designed and synthesised a series of chromone-nitrogen mustard derivatives, and evaluated their antiproliferative activities as well as further mechanisms on breast cancer cells in order to obtain candidate compounds with stronger antitumor activities and lower side effects.

## 2. Experimental

### 2.1. Chemistry

All chemical materials and reagents were purchased from commercial suppliers and used directly without purification. Anhydrous reagents were prepared by routine laboratory methods. Bruker ARX-400 NMR spectrometer (Bruker, Karlsruhe, Germany) was used to measure ^1^H and ^13^C NMR spectra, tetramethylsilane (TMS) was chosen as the internal standard, and *δ* was used to report chemical shifts. Agilent QTOF6520 high-resolution mass spectrometer (Agilent Technologies, Palo Alto, CA) was used to measure high-resolution mass spectra (HR-MS).

#### 2.1.1. General procedures for the synthesis of compounds 17a–e, 18a–e, 21a–e and 22a–e

DMAP (0.13 mmol), EDCI (0.80 mmol) and corresponding nitrogen mustard derivatives (**4, 6**, **7**, **9** or **10**, 0.26 mmol) were successively added to **15** or **16** dissolved in anhydrous DCM (5 ml) and stirred at room temperature overnight. When the reaction was complete, water (10 ml) and DCM (10 ml × 3) were added to extract. The organic layer was combined, washed with saturated brine, dried over anhydrous Na_2_SO_4_, filtered and concentrated *in vacuo* to obtain the crude product. It was purified by silica gel column chromatography (dichloromethane/methanol system) and concentrated to obtain target compounds **17a**–**e** and **18a**–**e**.

Compound **19** or **20** (0.14 mmol) was dissolved in anhydrous DCM (4 ml), and then corresponding nitrogen mustard derivatives (**4, 6**, **7**, **9** or **10**, 0.14 mmol), HOBt (0.17 mmol), EDCI (0.21 mmol) were added successively, and the mixture was stirred at room temperature for 6 h. After the reaction was completed, the reaction solution was poured into water (10 ml), extracted with DCM (10 ml × 3), washed with saturated brine, dried over Na_2_SO_4_, and concentrated *in vacuo* to get the crude product. Subsequently, the target compounds **21a–e** and **22a–e** were obtained by silica gel column chromatography (dichloromethane/methanol system).

#### 2.1.2. (6-Methyl-4-oxo-4H-chromen-3-yl)methyl 4-(bis(2-chloroethyl)amino)benzoate (17a)

White oil, yield: 66.5%. ^1^H NMR (400 MHz, CDCl_3_) *δ*: 8.12 (s, 1H, 2-H), 8.02 (d, 1H, *J* = 2.0 Hz, 5-H), 7.94 (d, 2H, *J* = 9.1 Hz, Ar-H), 7.47 (dd, 1H, *J* = 8.6, 2.0 Hz, 7-H), 7.34 (d, 1H, *J* = 8.6 Hz, 8-H), 6.63 (d, 2H, *J* = 9.1 Hz, Ar-H), 5.27 (s, 2H, -CH_2_), 3.78 (t, 4H, *J* = 7.0 Hz, -CH_2_), 3.64 (t, 4H, *J* = 7.0 Hz, -CH_2_), 2.45 (s, 3H, -CH_3_); ^13 ^C NMR (100 MHz, CDCl_3_) *δ*: 176.8, 166.4, 155.4, 154.8, 149.8, 135.4, 135.1, 132.0 (×2), 125.2, 123.8, 119.8, 118.6, 118.0, 110.9 (×2), 58.12, 53.3 (×2), 40.2 (×2), 21.0; HRMS (ESI) *m/z* calcd for C_22_H_20_Cl_2_NO_4_ [M-H]^−^ 432.0769, found 432.0748.

#### 2.1.3. (6-Methyl-4-oxo-4H-chromen-3-yl)methyl (S)-3–(4-(bis(2-chloroethyl)amino)phenyl)-2-formamidopropanoate (17b)

Yellow oil, yield: 35.4%. ^1^H NMR (400 MHz, CDCl_3_) *δ*: 8.18 (s, 1H, -CHO), 8.04 (d, 1H, *J* = 1.8 Hz, 5-H) , 7.95 (s, 1H, −2-H), 7.52 (dd, 1H, *J* = 8.6, 2.0 Hz, 7-H), 7.39 (d, 1H, *J* = 8.6 Hz, 8-H), 6.98 (d, 2H, *J* = 8.8 Hz, Ar-H), 6.50 (d, 2H, *J* = 8.8 Hz, Ar-H), 6.07 (d, 1H, *J* = 7.7 Hz, -NH), 5.16, 5.06 (d, each 1H, *J* = 12.4 Hz, -CH_2_), 4.92 (m, 1H, -CH), 3.62 (m, 4H, -CH_2_), 3.56 (m, 4H, -CH_2_), 3.08, 3.03 (dd, each 1H, *J* = 14.0, 5.5 Hz, -CH_2_), 2.47 (s, 3H, -CH_3_); ^13^C NMR (100 MHz, CDCl_3_) *δ*: 176.5, 171.3, 160.4, 156.0, 154.7, 145.1, 135.8, 135.4, 130.7 (×2), 125.3, 124.2, 123.7, 118.6, 118.0, 112.1 (×2), 59.4, 53.5 (×2), 52.0, 40.3 (×2), 36.7, 21.0; HRMS (ESI) *m/z* calcd for C_25_H_25_Cl_2_N_2_O_5_ [M-H]^−^ 503.1141, found 503.1129.

#### 2.1.4. (6-Methyl-4-oxo-4H-chromen-3-yl)methyl (S)-3-(4-(bis(2-chloroethyl)amino)phenyl)-2-((tert-butoxycarbonyl)amino)propanoate (17c)

Colourless oil, yield: 39.2%. ^1^H NMR (400 MHz, CDCl_3_) *δ*: 8.03 (d, 1H, *J* = 1.8 Hz, 5-H), 7.91 (s, 1H, 2-H), 7.51 (dd, 1H, *J* = 8.6, 1.8 Hz, 7-H), 7.38 (d, 1H, *J* = 8.6 Hz, 8-H), 6.97 (d, 2H, *J* = 8.7 Hz, Ar-H), 6.47 (d, 2H, *J* = 8.7 Hz, Ar-H), 5.13, 5.04 (d, each 1H, *J* = 12.7 Hz, -CH_2_), 4.99 (d, 1H, *J* = 7.8 Hz, -NH), 4.54, (m, 1H, -CH), 3.62 (m, 4H, -CH_2_), 3.55 (m, 4H, -CH_2_), 3.02, 2.95 (m, each 1H, -CH_2_), 2.46 (s, 3H, -CH_3_), 1.41 (s, 9H, *t*-Bu-H); ^13^C NMR (100 MHz, CDCl_3_) *δ*: 176.5, 172.1, 155.8, 155.1, 154.7, 145.0, 135.6, 135.3, 130.7 (×2), 125.2, 124.8, 123.7, 118.8, 118.0, 112.0 (×2), 79.9, 59.0, 54.6, 53.5 (×2), 40.3 (×2), 37.1, 28.3 (×3), 21.0; HRMS (ESI) *m/z* calcd for C_29_H_33_Cl_2_N_2_O_6_ [M-H] ^–^ 575.1716, found 575.1713.

#### 2.1.5. (6-Methyl-4-oxo-4H-chromen-3-yl)methyl (S)-4-((3-(4-(bis(2-chloroethyl)amino)phenyl)-1-methoxy-1-oxopropan-2-yl)amino)-4-oxobutanoate (17d)

Yellow solid, yield: 65.4%. ^1^H NMR (400 MHz, CDCl_3_) *δ*: 8.04 (s, 1H, 2-H), 8.00 (d, 1H, *J* = 2.0 Hz, 5-H), 7.48 (dd, 1H, *J* = 8.6, 2.0 Hz, 7-H), 7.35 (d, 1H, *J* = 8.6 Hz, 8-H), 6.98 (d, 2H, *J* = 8.8 Hz, Ar-H), 6.61 (d, 2H, *J* = 8.8 Hz, Ar-H), 6.11 (d, 1H, *J* = 7.8 Hz, -NH), 5.07 (s, 2H, -CH_2_), 4.80 (m, 1H, -CH), 3.72 (s, 3H, -OCH_3_), 3.70 (m, 4H, -CH_2_), 3.61 (m, 4H, -CH_2_), 3.04, 2.98 (dd, each 1H, *J* = 14.0, 5.7 Hz, -CH_2_), 2.66 (m, 2H, -CH_2_), 2.50 (t, 2H, *J* = 7.0 Hz, -CH_2_), 2.45 (s, 3H, -CH_3_); ^13 ^C NMR (100 MHz, CDCl_3_) *δ*: 176.7, 172.6, 172.1, 170.8, 155.4, 154.8, 144.9, 135.5, 135.2, 130.6 (×2), 125.1, 123.6, 119.1, 117.9, 112.3 (×2), 58.6, 53.6 (×2), 53.0, 52.3, 40.4 (×2), 36.7, 30.8, 29.4, 21.0; HRMS (ESI) *m/z* calcd for C_29_H_31_Cl_2_N_2_O_7_ [M-H] ^–^ 589.1508, found 589.1506.

#### 2.1.6. (6-Methyl-4-oxo-4H-chromen-3-yl)methyl (S)-5-((3-(4-(bis(2-chloroethyl)amino)phenyl)-1-methoxy-1-oxopropan-2-yl)amino)-5-oxopentanoate (17e)

White oil, yield: 46.7%. ^1^H NMR (400 MHz, CDCl_3_) *δ*: 8.05 (s, 1H, 2-H), 8.01 (d, 1H, *J* = 2.0 Hz, 5-H), 7.49 (dd, 1H, *J* = 8.6, 2.0 Hz, 7-H), 7.37 (d, 1H, *J* = 8.6 Hz, 8-H), 6.98 (d, 2H, *J* = 8.7 Hz, Ar-H), 6.61 (d, 2H, *J* = 8.7 Hz, Ar-H), 6.19 (d, 1H, *J* = 8.0 Hz, -NH), 5.03 (s, 2H, -CH_2_), 4.82 (m, 1H, -CH), 3.71 (s, 3H, -OCH_3_), 3.69 (m, 4H, -CH_2_), 3.60 (m, 4H, -CH_2_), 3.05, 2.95 (dd, each 1H, *J* = 13.9, 5.7 Hz, -CH_2_), 2.45 (s, 3H, -CH_3_), 2.24 (t, 2H, *J* = 7.1 Hz, -CH_2_), 1.92 (m, 2H, -CH_2_); ^13 ^C NMR (100 MHz, CDCl_3_) *δ*: 176.9, 173.1, 172.2, 171.8, 155.7, 154.8, 144.8, 135.6, 135.3, 130.6 (×2), 125.2, 123.7, 119.2, 118.0, 112.4 (×2), 58.5, 53.7 (×2), 53.2, 52.3, 40.3 (×2), 36.8, 35.1, 33.1, 21.0, 20.9; HRMS (ESI) *m/z* calcd for C_30_H_33_Cl_2_N_2_O_7_ [M-H] ^–^ 603.1665, found 603.1667.

#### 2.1.7. (6-Methoxy-4-oxo-4H-chromen-3-yl)methyl 4-(bis(2-chloroethyl)amino)benzoate (18a)

Yellow oil, yield: 43.8%. ^1^H NMR (400 MHz, CDCl_3_) *δ*: 8.14 (s, 1H, 2-H), 7.95 (d, 2H, *J* = 9.1 Hz, Ar-H), 7.61 (d, 1H, *J* = 3.1 Hz, 5-H), 7.40 (d, 1H, *J* = 9.1 Hz, 8-H), 7.26 (dd, 1H, *J* = 9.1, 3.1 Hz, 7-H), 6.64 (d, 2H, *J* = 9.1 Hz, Ar-H), 5.27 (s, 2H, -CH_2_), 3.90 (s, 3H, -OCH_3_), 3.79 (t, 4H, *J* = 6.8 Hz, -CH_2_), 3.64 (t, 4H, *J* = 6.8 Hz, -CH_2_); ^13 ^C NMR (100 MHz, CDCl_3_) *δ*: 176.6, 166.4, 157.1, 155.3, 151.3, 149.8, 132.0 (×2), 124.7, 124.0, 119.6, 119.2, 118.6, 110.9 (×2), 105.0, 58.1, 56.0, 53.3 (×2), 40.1 (×2); HRMS (ESI) *m/z* calcd for C_22_H_20_Cl_2_NO_5_ [M-H] ^–^ 448.0719, found 448.0714.

#### 2.1.8. (6-Methoxy-4-oxo-4H-chromen-3-yl)methyl (S)-3-(4-(bis(2-chloroethyl)amino)phenyl)-2-formamidopropanoate (18b)

Yellow oil, yield: 39.2%. ^1^H NMR (400 MHz, CDCl_3_) *δ*: 8.18 (s, 1H, -CHO), 7.96 (s, 1H, 2-H), 7.60 (d, 1H, *J* = 3.1 Hz, 5-H), 7.43 (d, 1H, *J* = 9.1 Hz, 8-H), 7.30 (dd, 1H, *J* = 9.1, 3.1 Hz, 7-H), 6.98 (d, 2H, *J* = 8.7 Hz, Ar- H), 6.50 (d, 2H, *J* = 8.7 Hz, Ar-H) , 6.06 (d, 1H, *J* = 7.6 Hz, -NH), 5.16, 5.08 (d, each 1H, *J* = 12.4 Hz, -CH_2_), 4.92 (m, 1H, -CH), 3.91 (s, 3H, -OCH_3_), 3.63 (m, 4H, -CH_2_), 3.57 (m, 4H, -CH_2_), 3.09, 3.03 (m, each 1H, -CH_2_); ^13 ^C NMR (100 MHz, CDCl_3_) *δ*: 176.3, 171.3, 160.5, 157.3, 155.8, 151.3, 145.2, 130.7 (×2), 124.6, 124.2, 124.2, 119.7, 118.0, 112.0 (×2), 105.0, 59.4, 56.0, 53.4 (×2), 52.0, 40.3 (×2), 36.7; HRMS (ESI) *m/z* calcd for C_25_H_25_Cl_2_N_2_O_6_ [M-H] ^–^ 519.1090, found 519.1094.

#### 2.1.9. (6-Methoxy-4-oxo-4H-chromen-3-yl)methyl (S)-3-(4-(bis(2-chloroethyl)amino)phenyl)-2-((tert-butoxycarbonyl)amino)propanoate (18c)

Colourless oil, yield: 35.9%. ^1^H NMR (400 MHz, CDCl_3_) *δ*: 7.91 (s, 1H, 2-H), 7.59 (d, 1H, *J* = 3.1 Hz, 5-H), 7.41 (d, 1H, *J* = 9.2 Hz, 8-H), 7.28 (dd, 1H, *J* = 9.2, 3.1 Hz, 7-H), 6.97 (d, 2H, *J* = 8.7 Hz, Ar-H), 6.49 (d, 2H, *J* = 8.7 Hz, Ar-H), 5.14, 5.05 (d, each 1H, *J* = 12.6 Hz, -CH_2_), 5.00 (d, 1H, *J* = 8.2 Hz, -NH), 4.54 (m, 1H, -CH), 3.89 (s, 3H, -OCH_3_), 3.63 (m, 4H, -CH_2_), 3.55 (m, 4H, -CH_2_), 3.01, 2.96 (m, each 1H, -CH_2_), 1.41 (s, 9H, *t*-Bu-H); ^13 ^C NMR (100 MHz, CDCl_3_) *δ*: 176.3, 172.1, 157.2, 155.6, 155.1, 151.3, 145.0, 130.7 (×2), 124.8, 124.6, 124.1, 119.6, 118.2, 112.0 (×2), 105.0, 79.9, 59.0, 56.0, 54.6, 53.5 (×2), 40.3 (×2), 37.1, 28.3 (×3); HRMS (ESI) *m/z* calcd for C_29_H_33_Cl_2_N_2_O_7_ [M-H] ^–^ 591.1665, found 591.1677.

#### 2.1.10. (6-Methoxy-4-oxo-4H-chromen-3-yl)methyl (S)-4-((3-(4-(bis(2-chloroethyl)amino)phenyl)-1-methoxy-1-oxopropan-2-yl)amino)-4-oxobutanoate (18d)

Yellow solid, yield: 62.3%. ^1^H NMR (400 MHz, CDCl_3_) *δ*: 8.05 (s, 1H, 2-H), 7.58 (d, 1H, *J* = 3.1 Hz, 5-H), 7.39 (d, 1H, *J* = 9.2 Hz, 8-H), 7.26 (dd, 1H, *J* = 9.2, 3.1 Hz, 7-H), 6.98 (d, 2H, *J* = 8.7 Hz, Ar-H), 6.62 (d, 2H, *J* = 8.7 Hz, Ar-H), 6.08 (d, 1H, *J* = 7.9 Hz, -NH), 5.08 (s, 2H, -CH_2_), 4.80 (m, 1H, -CH), 3.89 (s, 3H, -OCH_3_), 3.72 (s, 3H, -OCH_3_), 3.70 (m, 4H, -CH_2_), 3.62 (m, 4H, -CH_2_), 3.04, 2.99 (m, each 1H, -CH_2_), 2.67 (m, 2H, -CH_2_), 2.51 (m, 2H, -CH_2_); ^13 ^C NMR (100 MHz, CDCl_3_) *δ*: 176.5, 172.6, 172.0, 170.8, 157.1, 155.3, 151.3, 144.9, 130.6 (×2), 125.1, 124.6, 124.0, 119.6, 118.6, 112.4 (×2), 104.9, 58.6, 55.9, 53.7 (×2), 53.3, 52.3, 40.3 (×2), 36.7, 30.8, 29.4; HRMS (ESI) *m/z* calcd for C_29_H_31_Cl_2_N_2_O_8_ [M-H] ^–^ 605.1457, found 605.1450.

#### 2.1.11. (6-Methoxy-4-oxo-4H-chromen-3-yl)methyl (S)-5-((3-(4-(bis(2-chloroethyl)amino)phenyl)-1-methoxy-1-oxopropan-2-yl)amino)-5-oxopentanoate (18e)

Colourless oil, yield: 64.7%. ^1^H NMR (400 MHz, CDCl_3_) *δ*: 8.07 (s, 1H, 2-H), 7.60 (d, 1H, *J* = 3.1 Hz, 5-H), 7.41 (d, 1H, *J* = 9.1 Hz, 8-H), 7.28 (dd, 1H, *J* = 9.1, 3.1 Hz, 7-H), 6.98 (d, 2H, *J* = 8.7 Hz, Ar-H), 6.61 (d, 2H, *J* = 8.7 Hz, Ar-H), 6.17 (d, 1H, *J* = 7.9 Hz, -NH), 5.05 (s, 2H, -CH_2_), 4.82 (m, 1H, -CH), 3.89 (s, 3H, -OCH_3_), 3.71 (s, 3H, -OCH_3_), 3.68 (m, 4H, -CH_2_), 3.61 (m, 4H, -CH_2_), 3.06, 2.96 (m, each 1H, -CH_2_), 2.36 (m, 2H, -CH_2_), 2.25 (m, 2H, -CH_2_), 1.93 (m, 2H, -CH_2_); ^13 ^C NMR (100 MHz, CDCl_3_) *δ*: 176.7, 173.1, 172.2, 171.8, 157.2, 155.5, 151.4, 144.9, 130.5 (×2), 125.2, 124.7, 124.1, 119.7, 118.6, 112.4 (×2), 105.0, 58.5, 56.0, 53.6, 53.1 (×2), 52.3, 40.3 (×2), 36.8, 35.1, 33.1, 20.9; HRMS (ESI) *m/z* calcd for C_30_H_33_Cl_2_N_2_O_8_ [M-H] ^–^ 619.1614, found 619.1616.

#### 2.1.12. 4-(Bis(2-chloroethyl)amino)-N-((6-methyl-4-oxo-4H-chromen-3-yl)methyl)benzamide (21a)

White solid, yield: 36.8%. ^1^H NMR (400 MHz, CDCl_3_) *δ*: 8.17 (s, 1H, 2-H), 7.98 (d, 1H, *J* = 2.0 Hz, 5-H), 7.70 (d, 2H, *J* = 8.9 Hz, Ar-H), 7.48 (dd, 1H, *J* = 8.6, 2.0 Hz, 7-H), 7.36 (d, 1H, *J* = 8.6 Hz, 8-H), 7.14 (s, 1H, -NH), 6.64 (d, 2H, *J* = 8.9 Hz, Ar-H), 4.45 (d, 2H, *J* = 5.6 Hz, -CH_2_), 3.76 (m, 4H, -CH_2_), 3.62 (m, 4H, -CH_2_), 2.45 (s, 3H, -CH_3_); ^13 ^C NMR (100 MHz, CDCl_3_) *δ*: 178.5, 167.0, 154.9, 154.5, 148.7, 135.3, 135.2, 129.1 (×2), 124.8 (×2), 123.6, 121.0, 118.1, 111.1 (×2), 53.3 (×2), 40.2 (×2), 36.3, 20.9; HRMS (ESI) *m/z* calcd for C_22_H_21_Cl_2_N_2_O_3_ [M-H] ^–^ 431.0929, found 431.0921.

#### 2.1.13. (S)-3-(4-(bis(2-chloroethyl)amino)phenyl)-2-formamido-N-((6-methyl-4-oxo-4H-chromen-3-yl)methyl)propanamide (21b)

White solid, yield: 61.0%. ^1^H NMR (400 MHz, CDCl_3_) *δ*: 8.18 (s, 1H, -CHO), 7.99 (s, 1H, 2-H), 7.96 (d, 1H, *J* = 2.1 Hz, 5-H), 7.51 (dd, 1H, *J* = 8.6, 2.1 Hz, 7-H), 7.38 (d, 1H, *J* = 6.8 Hz, 8-H), 6.95 (d, 2H, *J* = 8.7 Hz, Ar-H), 6.73 (m, 1H, -NH), 6.44 (m, 1H, -NH), 6.42 (d, 2H, *J* = 8.7 Hz, Ar-H), 4.68 (m, 1H, -CH), 4.24, 4.17 (dd, each 1H, *J* = 14.3, 6.5 Hz, -CH_2_), 3.54 (m, 8H, -CH_2_), 3.00, 2.90 (dd, each 1H, *J* = 14.0, 5.5 Hz, -CH_2_), 2.47 (s, 3H, -CH_3_); ^13^C NMR (100 MHz, CDCl_3_) *δ*: 177.7, 170.5, 160.6, 154.8, 154.4, 145.0, 135.5, 135.3, 130.6 (×2), 124.9, 124.8, 123.5, 120.4, 118.1, 112.0 (×2), 53.4 (×2), 53.1, 40.3 (×2), 37.6, 35.7, 21.0; HRMS (ESI) *m/z* calcd for C_25_H_26_Cl_2_N_3_O_4_ [M-H] ^–^ 502.1300, found 502.1294.

#### 2.1.14. Tert-butyl (S)-(3-(4-(bis(2-chloroethyl)amino)phenyl)-1-(((6-methyl-4-oxo-4H-chromen-3-yl)methyl)amino)-1-oxopropan-2-yl)carbamate (21c)

White oil, yield: 49.4%. ^1^H NMR (400 MHz, CDCl_3_) *δ*: 8.00 (s, 1H, 2-H), 7.95 (d, 1H, *J* = 2.1 Hz, 5-H), 7.50 (dd, 1H, *J* = 8.6, 2.1 Hz, 7- H), 7.37 (d, 1H, *J* = 8.6 Hz, 8-H), 6.96 (d, 2H, *J* = 8.6 Hz, Ar-H), 6.64 (m, 1H, -NH), 6.43 (d, 2H, *J* = 8.6 Hz, Ar-H), 5.00 (s, 1H, -NH), 4.2 (m, 3H, -CH_2_, -CH), 3.55 (m, 8H, -CH_2_), 2.97, 2.88 (m, each 1H, -CH_2_), 2.46 (s, 3H, -CH_3_), 1.39 (s, 9H, *t*-Bu-H); ^13 ^C NMR (100 MHz, CDCl_3_) *δ*: 177.7, 171.5, 154.8, 154.3, 144.9, 135.3, 135.2, 130.6 (×2), 125.2, 124.9, 123.6, 120.6, 118.0, 112.0 (×2), 80.1, 55.8, 53.4 (×2), 40.3 (×2), 37.6, 35.4, 29.7, 28.3 (×3), 21.0; HRMS (ESI) *m/z* calcd for C_29_H_34_Cl_2_N_3_O_5_ [M-H] ^–^ 574.1876, found 574.1889.

#### 2.1.15. Methyl (S)-3-(4-(bis(2-chloroethyl)amino)phenyl)-2-(4-(((6-methyl-4-oxo-4H-chromen-3-yl)methyl)amino)-4-oxobutanamido)propanoate (21d)

White oil, yield: 54.4%. ^1^H NMR (400 MHz, CDCl_3_) *δ*: 8.04 (s, 1H, 2-H), 7.96 (d, 1H, *J* = 2.1 Hz, 5-H), 7.47 (dd, 1H, *J* = 8.6, 2.1 Hz, 7-H), 7.33 (d, 1H, *J* = 8.6 Hz, 8-H), 6.96 (d, 2H, *J* = 8.7 Hz, Ar- H), 6.77 (m, 1H, -NH), 6.59 (d, 2H, *J* = 8.7 Hz, Ar-H), 6.39 (d, 1H, *J* = 7.7 Hz, -NH), 4.76 (m, 1H, -CH), 4.24 (d, 2H, *J* = 6.1 Hz, -CH_2_), 3.68 (m, 7H, -CH_2_, -OCH_3_), 3.60 (m, 4H, -CH_2_), 3.00, 2.94 (m, each 1H, -CH_2_), 2.50 (m, 2H, -CH_2_), 2.47 (m, 2H, -CH_2_), 2.44 (s, 3H, -CH_3_); ^13 ^C NMR (100 MHz, CDCl_3_) *δ*: 178.1, 172.2, 172.1, 171.7, 154.9, 154.3, 145.1, 135.3, 135.2, 130.5 (×2), 124.9, 124.8, 123.6, 120.8, 118.0, 112.2 (×2), 53.5 (×2), 53.4, 52.2, 40.4 (×2), 36.7, 35.8, 31.4, 31.3, 20.9; HRMS (ESI) *m/z* calcd for C_29_H_32_Cl_2_N_3_O_6_ [M-H] ^–^ 588.1668, found 588.1677.

#### 2.1.16. Methyl (S)-3-(4-(bis(2-chloroethyl)amino)phenyl)-2–(5-(((6-methyl-4-oxo-4H-chromen-3-yl)methyl)amino)-5-oxopentanamido)propanoate (21e)

White solid, yield: 37.3%. ^1^H NMR (400 MHz, CDCl_3_) *δ*: 8.14 (s, 1H, 2-H), 7.99 (d, 1H, *J* = 2.0 Hz, 5-H), 7.51 (dd, 1H, *J* = 8.6, 2.1 Hz, 7-H), 7.39 (d, 1H, *J* = 8.6 Hz, 8-H), 7.22 (m, 1H, -NH), 7.17 (d, 2H, *J* = 8.7 Hz, Ar-H), 6.87 (d, 1H, *J* = 8.2 Hz, -NH), 6.64 (d, 2H, *J* = 8.7 Hz, Ar-H), 4.84 (m, 1H, -CH), 4.23, 4.13 (m, each 1H, -CH_2_), 3.76 (s, 3H, -OCH_3_), 3.71 (m, 4H, -CH_2_), 3.62 (m, 4H, -CH_2_), 3.16, 3.10 (m, each 1H, -CH_2_), 2.48 (s, 3H, -CH_3_), 2.10 (m, 2H, -CH_2_), 1.95 (m, 2H, -CH_2_), 1.83 (m, 2H, -CH_2_); ^13 ^C NMR (100 MHz, CDCl_3_) *δ*: 178.6, 173.7, 173.1, 172.6, 155.3, 155.0, 145.1, 135.4, 135.3, 130.4 (×2), 125.6, 124.8, 123.7, 120.7, 118.2, 112.1 (×2), 53.6 (×2), 52.5, 40.4 (×2), 36.5, 36.3, 34.7, 34.4, 29.7, 22.0, 21.1; HRMS (ESI) *m/z* calcd for C_30_H_34_Cl_2_N_3_O_6_ [M-H] ^–^ 602.1825, found 602.1837.

#### 2.1.17. 4-(Bis(2-chloroethyl)amino)-N-((6-methoxy-4-oxo-4H-chromen-3-yl)methyl)benzamide (22a)

White solid, yield: 43.5%. ^1^H NMR (400 MHz, CDCl_3_) *δ*: 8.17 (s, 1H, 2-H), 7.70 (d, 2H, *J* = 8.8 Hz, Ar-H), 7.55 (d, 1H, *J* = 3.1 Hz, 5-H), 7.40 (d, 1H, *J* = 9.1 Hz, 8-H), 7.27 (dd, 1H, *J* = 9.1, 3.1 Hz, 7-H), 7.11 (s, 1H, -NH), 6.64 (d, 2H, *J* = 8.8 Hz, Ar-H), 4.46 (d, 2H, *J* = 5.7 Hz, -CH_2_), 3.89 (s, 3H, -OCH_3_), 3.76 (m, 4H, -CH_2_), 3.62 (m, 4H, -CH_2_); ^13 ^C NMR (100 MHz, CDCl_3_) *δ*: 178.3, 167.0, 157.0, 154.4, 151.6, 148.7, 129.1 (×2), 124.6, 124.1, 120.4, 119.8, 111.1 (×2), 104.5, 55.9, 53.3 (×2), 40.1 (×2), 36.3, 29.7; HRMS (ESI) *m/z* calcd for C_22_H_21_Cl_2_N_2_O_4_ [M-H] ^–^ 447.0878, found 447.0878.

#### 2.1.18. (S)-3-(4-(bis(2-chloroethyl)amino)phenyl)-2-formamido-N-((6-methoxy-4-oxo-4H-chromen-3-yl)methyl)propanamide (22b)

White oil, yield: 51.2%. ^1^H NMR (600 MHz, CDCl_3_) *δ*: 8.18 (s, 1H, -CHO), 7.99 (s, 1H, 2-H), 7.52 (d, 1H, *J* = 2.8 Hz, 5-H), 7.43 (d, 1H, *J* = 9.0 Hz, 8-H), 7.30 (dd, 1H, *J* = 9.0, 2.8 Hz, 7-H), 6.97 (d, 2H, *J* = 8.3 Hz, Ar-H), 6.64 (m, 1H, -NH), 6.44 (d, 2H, *J* = 8.3 Hz, Ar-H), 6.30 (d, 1H, *J* = 6.1 Hz, -NH), 4.66 (m, 1H, -CH), 4.23 (m, 2H, -CH_2_), 3.90 (s, H, -OCH_3_), 3.55 (m, 8H, -CH_2_), 3.01, 2.91 (m, each 1H, -CH_2_); ^13 ^C NMR (150 MHz, CDCl_3_) *δ*: 117.5, 170.5, 160.6, 157.1, 154.1, 151.4, 145.0, 130.6 (2), 124.7, 124.4, 124.1, 119.8, 119.7, 112.0 (×2), 104.7, 56.0, 53.4 (×2), 53.1, 40.3 (×2), 37.5, 35.7; HRMS (ESI) *m/z* calcd for C_25_H_26_Cl_2_N_3_O_5_ [M-H] ^–^ 518.1250, found 518.1246.

#### 2.1.19. Tert-butyl (S)-(3-(4-(bis(2-chloroethyl)amino)phenyl)-1-(((6-methoxy-4-oxo-4H-chromen-3-yl)methyl)amino)-1-oxopropan-2-yl)carbamate (22c)

Colourless oil, yield: 34.6%. ^1^H NMR (400 MHz, CDCl_3_) *δ*: 8.00 (s, 1H, 2-H), 7.52 (d, 1H, *J* = 3.0 Hz, 5-H), 7.42 (d, 1H, *J* = 9.2 Hz, 8-H), 7.28 (dd, 1H, *J* = 9.2, 3.0 Hz, 7-H), 6.98 (d, 2H, *J* = 8.7 Hz, Ar-H), 6.63 (m, 1H, -NH), 6.46 (d, 2H, *J* = 8.7 Hz), 4.97 (s, 1H, -NH), 4.23 (m, 2H, -CH_2_), 3.89 (s, 3H, -OCH_3_), 3.56 (m, 8H, -CH_2_), 2.96, 2.89 (m, each 1H, -CH_2_), 1.39 (s, 9H, *t*-Bu-H); ^13 ^C NMR (100 MHz, CDCl_3_) *δ*: 177.5, 171.5, 157.0, 154.1 (×2), 151.4, 144.9, 130.6 (×2), 125.2, 124.5, 124.0, 120.1, 119.7, 112.1 (×2), 104.7, 55.9 (×2), 53.4 (×2), 40.3 (×2), 37.5, 35.4, 29.7, 28.3 (×3); HRMS (ESI) *m/z* calcd for C_29_H_34_Cl_2_N_3_O_6_ [M-H] ^–^ 590.1825, found 590.1823.

#### 2.1.20. Methyl (S)-3-(4-(bis(2-chloroethyl)amino)phenyl)-2-(4-(((6-methoxy-4-oxo-4H-chromen-3-yl)methyl)amino)-4-oxobutanamido)propanoate (22d)

White oil, yield: 36.5%. ^1^H NMR (400 MHz, CDCl_3_) *δ*: 8.06 (s, 1H, 2-H), 7.53 (d, 1H, *J* = 3.1 Hz, 5-H), 7.37 (d, 1H, *J* = 9.1 Hz, 8-H), 7.25 (dd, 1H, *J* = 9.1, 3.1 Hz, 7-H), 6.95 (d, 2H, *J* = 8.6 Hz, Ar-H), 6.73 (m, 1H, -NH), 6.58 (d, 2H, *J* = 8.6 Hz, Ar-H), 6.34 (d, 1H, *J* = 7.7 Hz, -NH), 4.76 (m, 1H, -CH), 4.25 (d, 2H, *J* = 6.0 Hz), 3.88 (s, 3H, -OCH_3_), 3.68 (m, 7H, -OCH_3_, -CH_2_), 3.60 (m, 4H, -CH_2_), 3.00, 2.94 (m, each 1H, -CH_2_), 2.52, 2.47 (m, each 2H, -CH_2_); ^13 ^C NMR (100 MHz, CDCl_3_) *δ*: 177.8, 172.1, 172.1, 171.5, 157.0, 154.1, 151.5, 145.1, 130.5 (×2), 124.8, 124.5, 124.0, 120.2, 119.7, 112.1 (×2), 104.6, 55.9, 53.5 (×2), 53.4, 52.2, 40.4 (×2), 36.7, 35.8, 31.4, 31.3; HRMS (ESI) *m/z* calcd for C_29_H_32_Cl_2_N_3_O_7_ [M-H] ^–^ 604.1617, found 604.1627.

#### 2.1.21. Methyl (S)-3-(4-(bis(2-chloroethyl)amino)phenyl)-2–(5-(((6-methoxy-4-oxo-4H-chromen-3-yl)methyl)amino)-5-oxopentanamido)propanoate (22e)

White oil, yield: 42.9%. ^1^H NMR (400 MHz, CDCl_3_) *δ*: 8.12 (s, 1H, 2-H), 7.56 (d, 1H, *J* = 3.1 Hz, 5-H), 7.43 (d, 1H, *J* = 9.2 Hz, 8-H), 7.30 (dd, 1H, *J* = 9.2, 3.1 Hz, 7-H), 7.12 (d, 2H, *J* = 8.7 Hz, Ar-H), 6.90 (d, 1H, *J* = 8.2 Hz, -NH), 6.62 (d, 2H, *J* = 8.2 Hz, Ar-H), 4.83 (m, 1H, -CH), 4.24, 4.15 (m, each 1H, -CH_2_), 3.89 (s, 3H, -OCH_3_), 3.73 (s, 3H, -OCH_3_), 3.70 (m, 4H, -CH_2_), 3.60 (m, 4H, -CH_2_), 3.14, 3.07 (m, each 1H, -CH_2_), 2.11 (m, 2H, -CH_2_), 1.99 (m, 2H, -CH_2_), 1.84 (m, 2H, -CH_2_); ^13 ^C NMR (100 MHz, CDCl_3_) *δ*: 178.3, 173.5, 173.4, 172.7, 157.1, 155.2, 151.6, 145.1, 130.4 (×2), 125.5, 124.6, 123.9, 119.9, 119.8, 112.2 (×2), 104.8, 55.9, 53.5 (×2), 52.5, 40.4 (×2), 36.5, 36.3, 34.7, 34.5, 29.7, 21.9; HRMS (ESI) *m/z* calcd for C_30_H_34_Cl_2_N_3_O_7_ [M-H] ^–^ 618.1774, found 618.1776.

### 2.2. Cck-8 assay

The CCK-8 assay was carried out to investigate the cytotoxic effects of all target compounds against human mammary gland tumour cell line MCF-7, TNBC cell line MDA-MB-231 and human normal breast cell line MCF-10A. The source of all cell lines was from KeyGEN Biotech, Beijing, China. These cells were cultured with DMEM medium (medium containing 10% (v/v) foetal bovine serum, 100 U/mL penicillin and 100 mg/mL streptomycin) in a humidified atmosphere with 5% CO_2_ at 37 °C. Exponentially growing cells were prepared into a cell suspension with the concentration of 5 × 10^4^ cells/mL, added to 96-well plates (100 μL/well), and then incubated at 37 °C and 5% CO_2_ for 24 h. After the cells were attached, different concentrations of test compounds (100 μL/well) were added and incubated for 72 h. Finally, CCK-8 (10 μL/well) was added, incubated for 3 h and then mixed for 10 min. The absorbance of each well was measured at 450 nm by microplate reader (BioTek Elx800, Winooski, VT), and inhibition rates were calculated. Inhibition rate (%) = [(A_negative control group_− A_experimental group_)/A_negative control group_] × 100%.

### 2.3. Cell-cycle analysis

MDA-MB-231 cells were placed in 6-well plates and incubated at 37 °C for 24 h, then different concentrations of **22e** (0, 0.475, 0.95 and 1.9 μM) were added, and the incubation continued for 72 h. The cell suspension was prepared by digestion, collection and washing in sequence. Subsequently, the cell suspension was fixed with 70% ethanol, washed with phosphate-buffered solution (PBS), incubated with Rnase A (100 μL), and water bathed at 37 °C for 30 min. Finally, PI (400 μL) was added and placed in the dark at 4 °C for 30 min, and the DNA content distribution was detected by flow cytometry (FACS Calibur Becton-Dickinson, Franklin Lake, NJ).

### 2.4. Cell apoptosis assay

MDA-MB-231 cells were cultured in 6-well plates at 37 °C for 24 h, and then different concentrations of **22e** (0, 0.475, 0.95 and 1.9 μM) were added. After 72 h of incubation, the cells were collected and suspended in binding buffer (500 μL). Finally, annexin V-fluorescein isothiocyanate (Annexin V-FITC, 5 μL) and propidium iodide (PI, 5 μL) were added, and the reaction was kept at room temperature in the dark for 10 min, then cell apoptosis was measured by flow cytometry.

### 2.5. Detection of ROS

MDA-MB-231 cells were seeded overnight in 6-well plates, treated with different concentrations of **22e** (0, 0.475, 0.95 and 1.9 μM) for another 72 h. After digestion and washing, the cell suspension was prepared and cultured with fluorescent probe 2′,7′-dichlorodihydrofluorescein diacetate (DCFH-DA, Sigma-Aldrich, Darmstadt, Germany) at 37 °C for 20 min. Then, after the cells were washed, the intracellular reactive oxygen species (ROS) was measured by flow cytometry at 488 nm (excitation wavelength) and 530 nm (emission wavelength).

### 2.6. Comet assay

DNA damage was detected by Comet Assay Kit (Keygen, Nanjing, China) according to the manufacturer’s instructions. Briefly, MDA-MB-231 cells were treated with **22e** (0, 0.475, 0.95 and 1.9 μM) for 72 h. The collected cells were fixed on CometSlide for 15 min at 4 °C. Subsequently, the cells were incubated in lysis solution at 4 °C for 90 min, the slides were electrophoresed for 20 min, fixed in ethanol for 5 min, and stained with Vista Green DNA Dye. Finally, the data were got by fluorescence microscope.

### 2.7. Wound healing assay

MDA-MB-231 cells were cultured in 6-well plates at 37 °C for 24 h. After the cells attached, different concentrations of **22e** (0, 0.475, 0.95 and 1.9 μM) were added. Sterile pipette tips were used to scratch evenly in 6-well plates, the floating cells were washed away with PBS, and the fresh culture medium was replaced. After incubation for 24 h, the cells were photographed and the migration distance was measured.

### 2.8. Transwell assay

MDA-MB-231 cells were seeded in 24-well transwell plates upper chambers, different concentrations of **22e** (0, 0.475, 0.95 and 1.9 μM) were incubated on the lower surface for 24 h. The cells in the upper surface of the membrane were removed, and the migrated or invaded cells of the membranes bottom surface were placed in 0.1% crystal violet for staining for 30 min. Finally, the cells that migrated or invaded of the chamber bottom were photographed and counted.

### 2.9. Adhesion assay

Different concentrations of **22e** (0, 0.475, 0.95 and 1.9 μM) were used to treat MDA-MB-231 cells for 72 h. Then the serum-free medium containing CaM stain was added, cell suspension (100 μL) was cultured to 96-well plates (2000 cells/well) for 1 h. Finally, plates were washed with PBS, fixed with 3.7% formaldehyde, washed with PBS again, and took pictures with fluorescence microscope.

## 3. Results and discussion

### 3.1. Chemistry

**Scheme 1.**
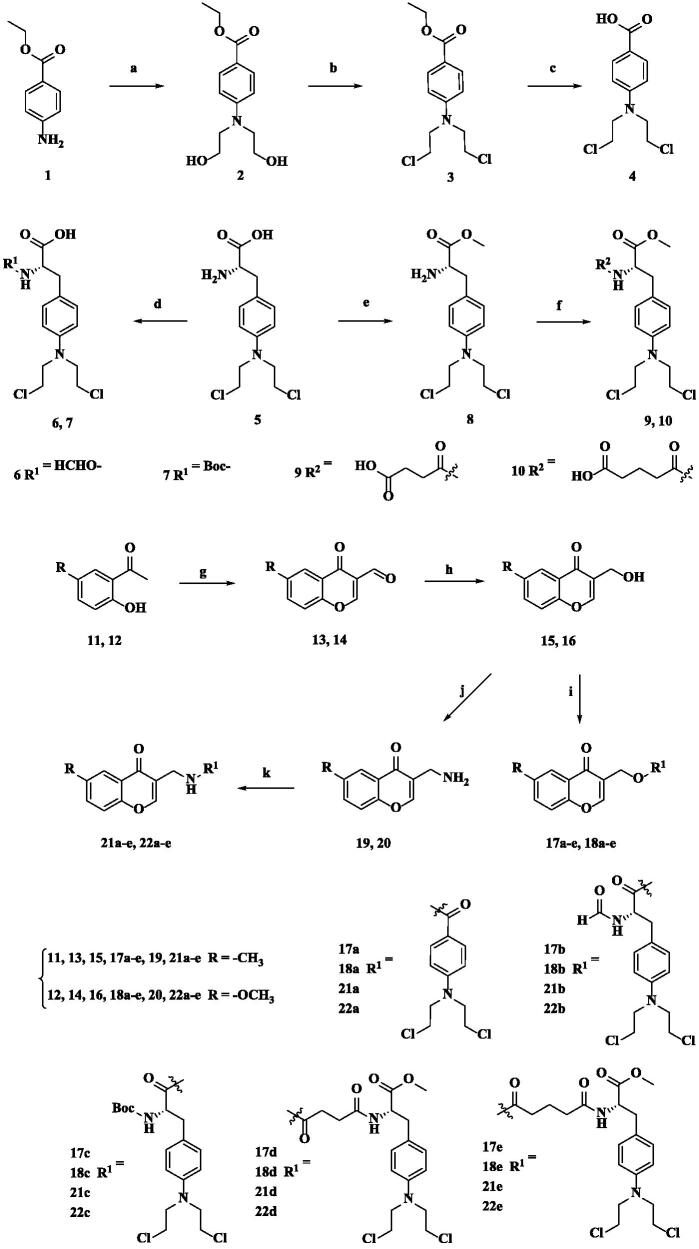
Synthesis of **4**, **6**, **7**, **9**, **10**, **17a–e**, **18a–e**, **21a–e** and **22a–e**. Reagents and conditions: (a) ethylene oxide, H_2_O, HCOOH, rt, 24 h; (b) POCl_3_, 50 °C, 0.5 h; (c) 10% HCl, rt, 12 h; (d) Ac_2_O, HCOOH, 50 °C, 5 h for compound **6**; Boc_2_O, TEA, dioxane, rt, 3 h for compound **7**; (e) SOCl_2_, MeOH, reflux, 12 h; (f) corresponding anhydride, DMAP, DCM, rt, overnight; (g) POCl_3_, DMF, −10 °C to rt, 8 h; (h) basic Al_2_O_3_, 2-propanol, 75 °C, 4 h; (i) **4**/**6**/**7**/**9**/**10**, EDCI, DMAP, anhydrous DCM, rt, overnight; (j) PBr_3_, 0 °C to rt, 15 h; NH_3_·H_2_O, DMF, rt, overnight; (k) **4**/**6**/**7**/**9**/**10**, EDCI, HOBt, anhydrous DCM, rt, 6 h.

The synthesis of the new target compounds is outlined in Scheme 1. Five nitrogen mustard derivatives **4**, **6**, **7**, **9** and **10** were prepared according to the literature^52–54^. Using compound **1** as the raw material for the reaction, benzoic acid mustard **4** was synthesised in a three-step sequence. The amino group of melphalan **5** was formylated and protected to obtain formylmerphalan **6** and Boc-protected melphalan **7**. The carboxyl group was methylated, and then reacted with the corresponding anhydride to obtain compounds **9** and **10** (Scheme 1).

Intermediates **13** and **14** were obtained from compounds **11** and **12** in the presence of Vilsmeier Haack reagent (POCl_3_ and DMF)^55^. The aldehyde group of Intermediates **13** and **14** was reduced to hydroxyl group by using alkaline aluminium oxide, which gave compounds **15** and **16,** and then its hydroxyl group was converted to amino group to give compounds **19** and **20** through one step reaction. The obtained compounds **15**, **16**, **19** and **20** were, respectively, subjected to esterification and amidation reactions with five nitrogen mustard derivatives **4**, **6**, **7**, **9** and **10** to synthetise target compounds **17a**-**e**, **18a–e**, **21a–e** and **22a–e** (Scheme 1). The target derivatives were identified by ^1^H NMR, ^13^C NMR and HR-MS.

### 3.2. Biological evaluation

#### 3.2.1. Antiproliferative activity

The antiproliferative activities of target compounds (**17a–e**, **18a–e**, **21a–e** and **22a–e**) against two human cancer cell lines (human mammary gland tumour cell line MCF-7 and TNBC cell line MDA-MB-231) were evaluated and compared with the chromone parent compounds (**15**, **16**, **19** and **20**), nitrogen mustard derivatives (**4**, **5**, **6**, **7**, **9** and **10)** and positive control doxorubicin. In addition, in order to better show the selective cytotoxicity between malignant and normal cells, the activity against normal human cell line MCF 10A was also evaluated.

As shown in Table 1, almost all target compounds were more potent than the corresponding chromone parent compounds and nitrogen mustard derivatives. Compounds **21b**, **21d**, **21e**, **22d** and **22e** showed strong IC_50_ values (3.07 μM, 2.99 μM, 2.15 μM, 2.60 μM, and 1.83 μM, respectively) against MCF-7 cells. The antiproliferative activities of a group of melphalan derivatives **17e**, **18e**, **21e** and **22e** against the two breast cancer cell lines ranged from 1.83 μM to 8.98 μM, which were stronger than almost all of the corresponding four groups of nitrogen mustard derivatives. Among the compounds **21a–e** and **22a–e** with amide bonds, **22a–e** with a methoxy group at the 6-position of chromone had stronger inhibitory activity to MDA-MB-231 cells than the corresponding compounds **21a–e** with methyl group. However, it would no longer be in line with this trend when ester bond was used as the connecting group. In addition, **22e** with amide bond as the linking group showed stronger antiproliferative activity than the corresponding compound **18e** with ester bond against two breast cancer cell lines, and other compounds generally followed this rule. It is worth noting that among all target compounds, **22e** showed the strongest antiproliferative activity against two breast cancer cell lines, with IC_50_ values of 1.83 and 1.90 μM, respectively. Especially, its inhibitory effect on MDA-MB-231 cells was much stronger than other compounds, which encourages us to further investigate the possible cellular mechanism.

**Table 1.** The antiproliferative effects of the target compounds and parent compounds against different cell lines.

Compound	IC_50_ (μM)^a^			SI	
	MCF-7	MDA-MB-231	MCF-10A	SI_(MCF-7)_^b^	SI_(MDA-MB-231)_^c^
**4**	12.13 ± 0.67	>20	>40	>3.3	NC^e^
**5**	>20	>20	>40	NC^e^	NC^e^
**6**	8.72 ± 0.57	>20	>40	>4.6	NC^e^
**7**	7.92 ± 0.38	>20	36.55 ± 1.47	4.6	NC^e^
**9**	>20	>20	>40	NC^e^	NC^e^
**10**	9.26 ± 0.45	>20	>40	>4.3	NC^e^
**15**	>20	>20	>40	NC^e^	NC^e^
**16**	>20	>20	>40	NC^e^	NC^e^
**19**	>20	>20	>40	NC^e^	NC^e^
**20**	>20	>20	>40	NC^e^	NC^e^
**17a**	7.58 ± 0.26	9.57 ± 0.65	24.16 ± 1.57	3.2	2.5
**17b**	5.90 ± 0.38	13.24 ± 1.12	36.68 ± 1.74	6.2	2.8
**17c**	8.56 ± 0.35	>20	>40	>4.7	NC^e^
**17d**	3.57 ± 0.15	11.42 ± 0.20	25.15 ± 1.42	7.0	2.2
**17e**	3.74 ± 0.15	8.32 ± 0.34	29.56 ± 1.67	7.9	3.6
**18a**	7.69 ± 0.26	9.47 ± 0.38	30.28 ± 1.46	3.9	3.2
**18b**	5.27 ± 0.43	11.54 ± 0.35	37.37 ± 1.35	7.1	3.2
**18c**	7.62 ± 0.28	16.74 ± 0.69	>40	>5.2	>2.4
**18d**	4.62 ± 0.36	12.78 ± 0.32	>40	>8.7	>3.1
**18e**	3.37 ± 0.16	8.98 ± 0.26	24.14 ± 1.25	7.2	2.7
**21a**	6.17 ± 0.53	14.87 ± 1.06	>40	>6.5	>2.7
**21b**	3.07 ± 0.18	9.42 ± 0.29	25.38 ± 1.75	8.3	2.7
**21c**	7.53 ± 0.31	12.67 ± 0.48	>40	>5.3	>3.2
**21d**	2.99 ± 0.16	6.87 ± 0.19	25.25 ± 1.80	8.4	3.7
**21e**	2.15 ± 0.12	4.47 ± 0.15	17.32 ± 1.03	8.1	3.9
**22a**	6.80 ± 0.30	10.57 ± 0.58	36.97 ± 3.14	5.4	3.5
**22b**	3.66 ± 0.19	4.65 ± 0.22	29.68 ± 1.48	8.1	6.4
**22c**	7.99 ± 0.16	9.87 ± 0.36	>40	>5.0	>4.1
**22d**	2.60 ± 0.15	3.05 ± 0.14	21.38 ± 1.24	8.2	7.0
**22e**	1.83 ± 0.11	1.90 ± 0.18	23.46 ± 1.73	12.8	12.3
**Adriamycin**	3.28 ± 0.15	2.94 ± 0.12	NT^d^		

^a^IC_50_: half inhibitory concentrations measured by the CCK-8 assay. The values are expressed as average ± standard deviation of three independent experiments.

^b^SI_MCF-7_: selectivity index between MCF-7 and MCF-10A. It was calculated as: SI = IC_50(MCF-10A)_/IC_50(MCF-7)_.

^c^SI_(MDA-MB-231)_: selectivity index between MCF-7 and MDA-MB-231. It was calculated as: SI = IC_50(MCF-10A)_/IC_50(MDA-MB-231)_.

^d^NT: not tested.

^e^NC: not calculated.

The selectivity index between MCF-7 or MDA-MB-231 cells and MCF-10A cells was calculated. Besides potent antiproliferative activity against tumour cells, **22e** also exhibited weak inhibitory effect to normal cell line MCF 10 A. As shown in Table 1, **22e** had both SI > 12, indicating that it had good inhibitory selectivity between tumour and normal cells.

#### 3.2.2. Cell-cycle analysis

**Figure 2.**
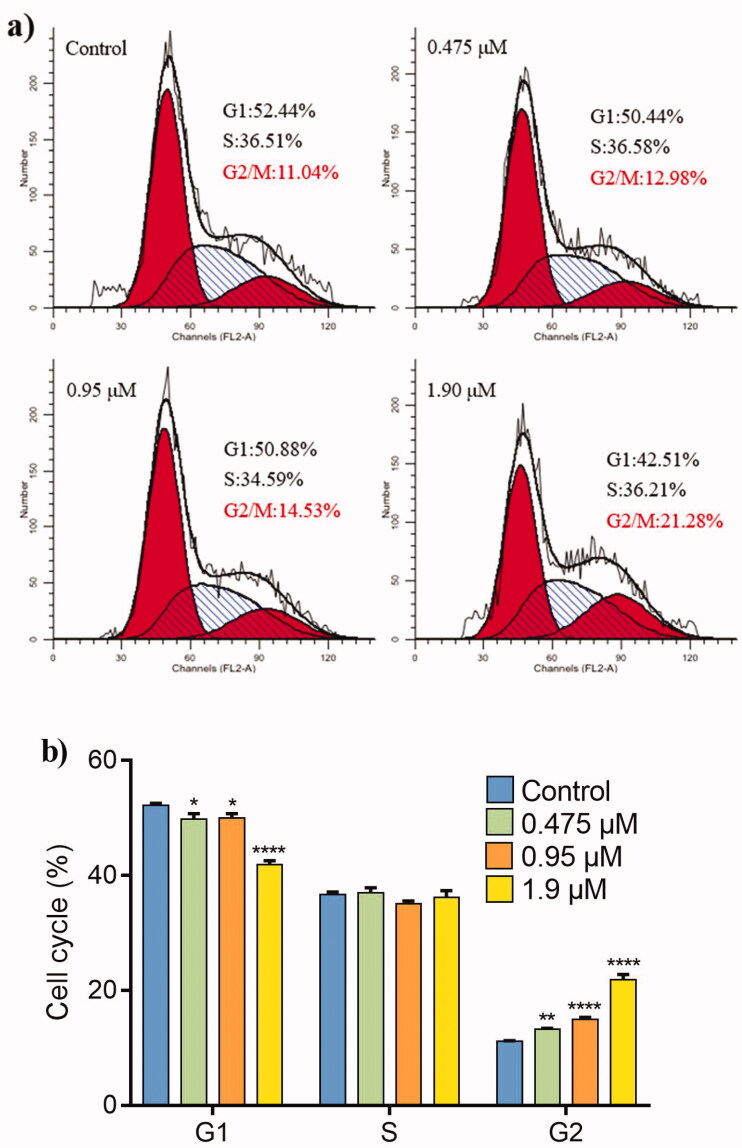
MDA-MB-231 cells were treated with **22e** (0, 0.475, 0.95 and 1.9 μM). (a) Cells were stained with PI and investigated by flow cytometry. (b) Histograms showed the cell-cycle distribution percentages. Data are represented as mean ± SD of three independent experiments. *p < 0.05, **p < 0.01, ****p < 0.0001 versus control group.

The occurrence of cancer due to uncontrolled cell proliferation is closely related to the cell cycle. Therefore, inhibition of tumour cell cycle is considered as a method of cancer treatment^56–58^. To determine whether **22e** affected cell cycle, MDA-MB-231 cells were treated with different concentrations of **22e** (0, 0.475, 0.95 and 1.9 μM), and flow cytometry was used to investigate PI staining-based cell-cycle analysis. As shown in Figure 2, as the concentration of **22e** increased, the cells in the G2/M phase increased from 11.04% in the control group to 21.28%. Meanwhile, the cells in the G1 phase decreased from 52.44% in the control group to 42.51%, while the cells in the S phase remained essentially unchanged. These results indicated that **22e** might exert antiproliferative effects by arresting cell cycle at the G2/M phase.

#### 3.2.3. Cell apoptosis assay

**Figure 3.**
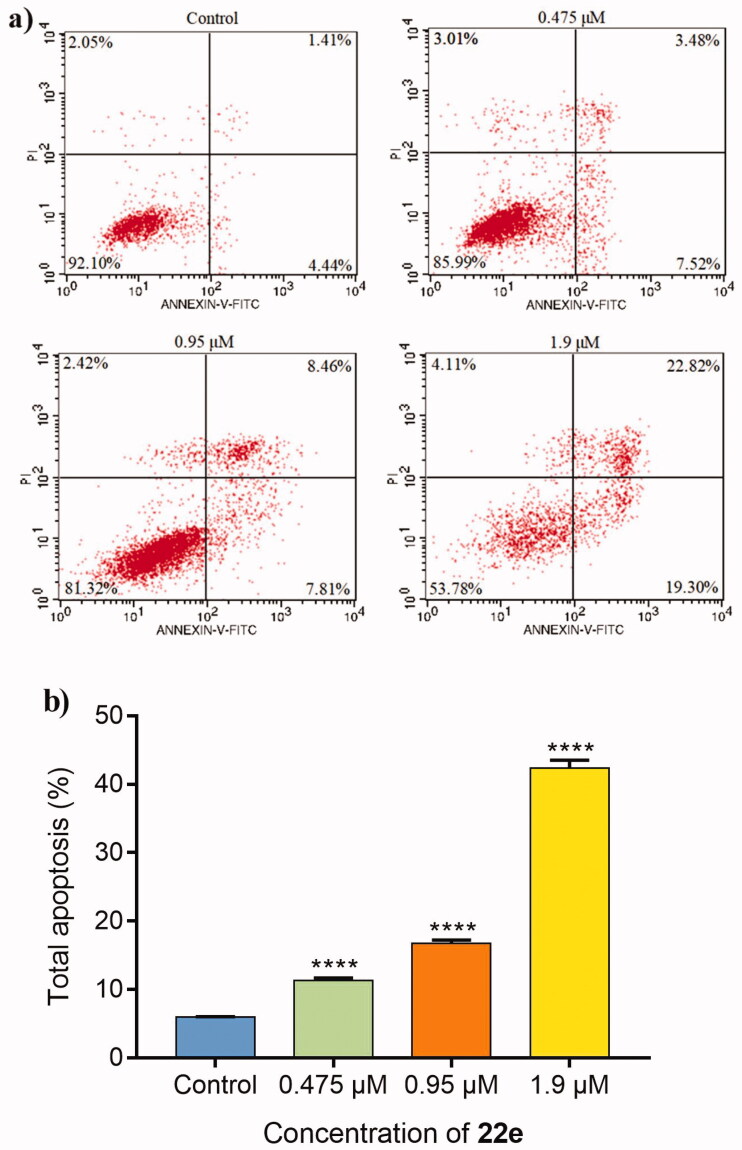
MDA-MB-231 cells were incubated with **22e** (0, 0.475, 0.95 and 1.9 μM). (a) Cells were stained with annexin V-FITC/PI and analysed by flow cytometry. (b) Histograms displayed the cell distribution percentage. Data are represented as mean ± SD of three independent experiments. ****p < 0.0001 versus control group.

At present, clinical oncology is still expected to eliminate cancer cells through apoptosis to treat cancer^59^. In order to investigate the effects of **22e** on induction of apoptosis, the annexin V-FITC/PI binding assay was performed. MDA-MB-231 cells were treated with different concentrations of **22e** (0, 0.475, 0.95 and 1.9 μM), and the percentage of apoptotic cells was determined by flow cytometry. As listed in Figure 3, with the concentration of **22e** increased, the total apoptosis ratios of MDA-MB-231 cells increased in a concentration-dependent manner, from 5.85% in the control group to 11.00%, 16.27%, and 42.12%, respectively. The results confirmed that **22e** could induce apoptosis of MDA-MB-231 cells.

#### 3.2.4. Detection of reactive oxygen species (ROS) generation and DNA damage

**Figure 4.**
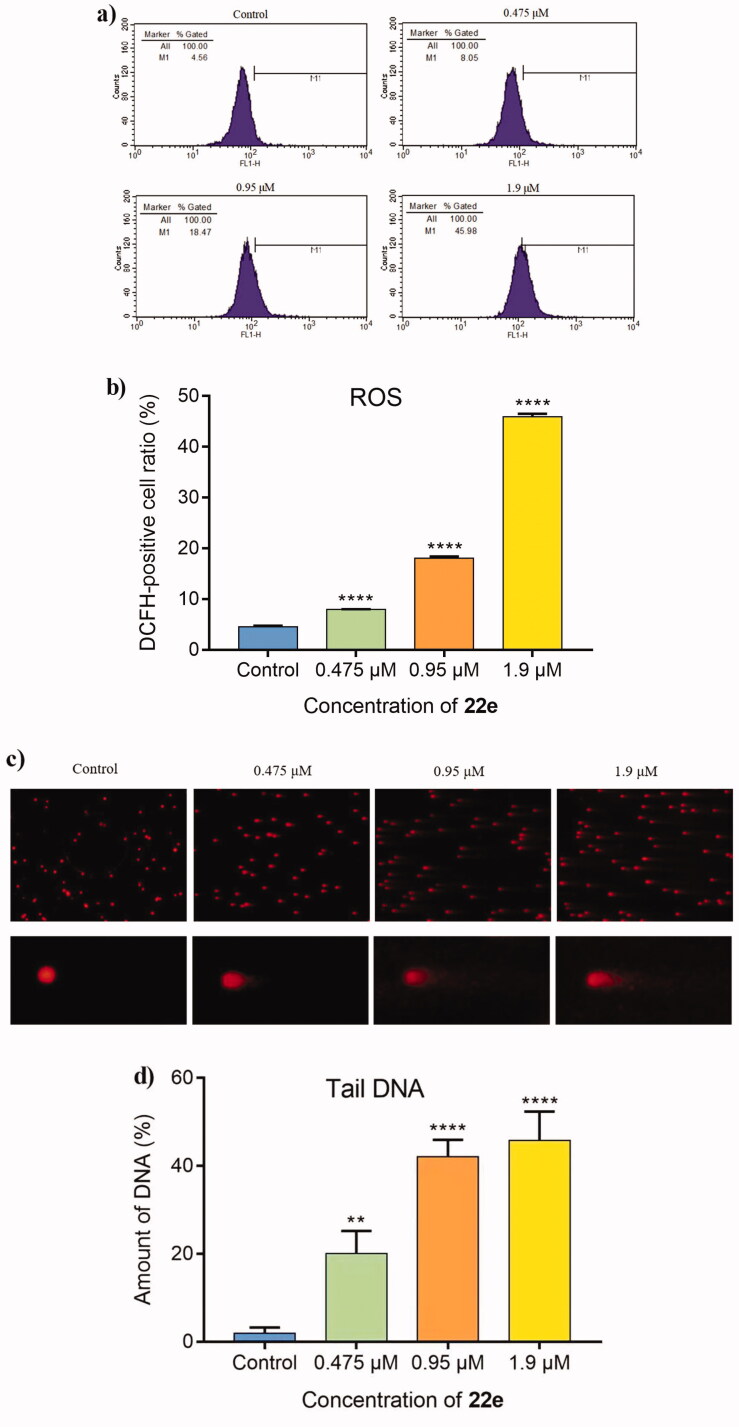
(a) MDA-MB-231 cells were treated with **22e** (0, 0.475, 0.95 and 1.9 μM), and then were cultured with DCFH-DA. The generation of ROS was measured by flow cytometry. (b) Corresponding histograms of DCFH-positive cell ratio were showed. (c) MDA-MB-231 cells were incubated with **22e** (0, 0.475, 0.95 and 1.9 μM). comet assay was used to evaluated DNA damage and photomicrographs were provided. (d) Tail DNA% were measured and showed in corresponding histograms. Data are represented as mean ± SD of three independent experiments. **p < 0.01, ****p < 0.0001 versus control group.

Compared with normal cells, cancer cells have higher levels of endogenous ROS, which makes them more susceptible to related ROS treatments^60^. To verify whether **22e** could induce the generation of intracellular ROS, MDA-MB-231 cells were treated with different concentrations of **22e** (0, 0.475, 0.95 and 1.9 μM), and then 2′,7′-dichlorodihydrofluorescein diacetate (DCFH-DA) was used to detect the level of intracellular ROS. As illustrated in Figure 4(a,b), **22e** could significantly increase the generation of intracellular ROS, which was positively correlated with the concentration of **22e**. With the increase of the concentration of **22e**, the DCFH-DA positive cells ratio increased from 4.56% in the control group to 8.05%, 18.47% and 45.98%, respectively. Through oxidative damage to intracellular biological macromolecules, such as proteins, lipids and DNA, excessive generation of ROS can lead to cell death^60^^,^^61^. We further used comet assay to investigate whether **22e** would cause DNA damage, and endogenous double-strand DNA breaks (DSBs) adducts were detected to evaluate DNA damage. As shown in Figure 4(c,d), **22e** increased the generation of DSBs. In addition, as the concentration of the compound increased, the tail DNA percentage increased from 0.48% to 19.95%, 38.15%, and 48.73%, respectively. These results preliminarily indicated that **22e** caused DNA damage by promoting the generation of intracellular ROS, and ultimately led to MDA-MB-231 cells apoptosis.

#### 3.2.5. Wound healing assay, transwell assay and adhesion assay

**Figure 5.**
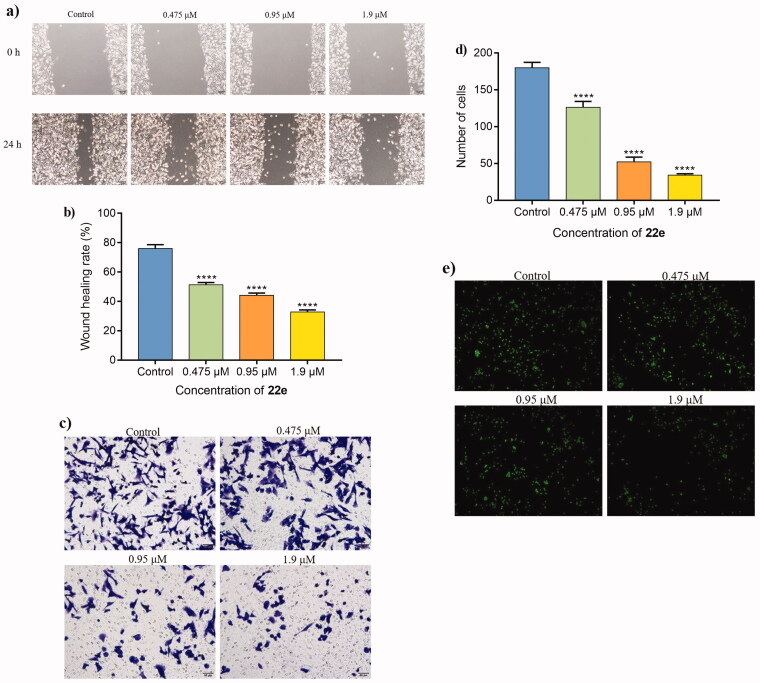
(a) MDA-MB-231 cells were treated with **22e** (0, 0.475, 0.95 and 1.9 μM), sterile pipette tips were used to scratch evenly, the incubation were continued, and representative images were captured (b) Corresponding histograms of wound-healing rate were showed. (c) MDA-MB-231 cells were seeded onto chambers and incubated with **22e** (0, 0.475, 0.95 and 1.9 μM), stained with crystal violet, and representative images were photographed. (d) The number of cells were displayed in corresponding histograms. (e) MDA-MB-231 cells were incubated with **22e** (0, 0.475, 0.95 and 1.9 μM), then fixed, washed and photographed with fluorescence microscope. All data are represented as the mean ± SD of three independent experiments. ****p < 0.0001 versus the control group.

Including TNBC, tumour invasion and metastasis are still the main causes of death in cancer patients^62^^,^^63^. First, to analyse the influence of **22e** to migration ability for MDA-MB-231 cells, we conducted wound healing assay to evaluate the cell migration ability by recording the wound closure distance. As illustrated in Figure 5(a,b), compared with untreated cells, the wound closure distance of cells treated with **22e** were significantly shortened, indicating that **22e** could inhibit the migration of MDA-MB-231 cells. In the next step, the effect of **22e** on the invasion ability of MDA-MB-231 cells was studied by the transwell assay. As shown in Figure 5(c,d), **22e** significantly inhibited MDA-MB-231 cells invasion and was positively correlated with the concentration. The adhesion of cancer cells is closely related to cancer invasion and metastasis, the adhesion effect of **22e** in MDA-MB-231 cells was also explored. Figure 5(e) reveals that **22e** could inhibit the adhesion of MDA-MB-231 cells in a concentration-dependent manner. Taken together, these results proved that **22e** significantly inhibited the migration, invasion and adhesion of MDA-MB-231 cells.

## 4. Conclusion

In general, we designed and synthesised a series of nitrogen mustard derivatives with chromone as the lead compound. The antiproliferative activities of all target derivatives on breast cancer cells MCF-7 and MDA-MB-231 were tested. Almost all compounds exhibited stronger antiproliferative activity than the parent compounds. The compound **22e**, which showed the strongest antiproliferative activity against the two cell lines with IC_50_ values of 1.83 and 1.90 μM, respectively, and showed low toxicity to the human normal cell MCF 10 A. Subsequently, preliminary mechanisms studies indicated that **22e** induced G2/M phase cell-cycle arrest and apoptosis in MDA-MB-231 cells. The DCFH-DA fluorescent probe assay and comet assay showed that **22e** induced DNA damage by intracellular ROS accumulation. In addition, **22e** also significantly inhibited the migration, invasion and adhesion of MDA-MB-231 cells *in vitro*. All considered, as a promising antimetastatic agent for TNBC, **22e** is worthy of further exploration.

## Supplementary Material

Supplemental Material
