# Supplementary material for: Design and synthesis of chromone-nitrogen mustard derivatives and evaluation of anti-breast cancer activity
Source: J Enzyme Inhib Med Chem. 2021 Dec 27;37(1):431–44. doi: 10.1080/14756366.2021.2018685 (PMC8725944; doi:10.1080/14756366.2021.2018685)
Supplement: Supplemental Material [file IENZ_A_2018685_SM9895.pdf]

Design and synthesis of chromone-nitrogen mustard deriveatives and evaluation of anti-breast cancer activity

Jianan Sun[a,1], Jiahui Mu[a,1], Shenglin Wang[a], Cai Jia[b,*], Dahong Li[a,*], Huiming Hua[a], Hao Cao[a,c,*]

[a]*Key Laboratory of Structure-Based Drug Design & Discovery, Ministry of Education, and School of Traditional Chinese Materia Medica, Shenyang Pharmaceutical University, 103 Wenhua Road, Shenyang 110016, P. R. China*

[b]*State Key Laboratory of Multiphase Complex Systems, Institute of Process Engineering, Chinese Academy of Sciences, Beijing 100190, P. R. China*

[c]*School of Life Science and Biopharmaceutics, Shenyang Pharmaceutical University, 103 Wenhua Road, Shenyang 110016, P. R. China*

*Corresponding author. E-mail address: jc@ipe.ac.cn (C. Jia); lidahong0203@163.com (D. Li); caohao2008@163.com (H. Cao)

Supplementary Data

^{1}H NMR, ^{13}C NMR and HR-MS spectrums

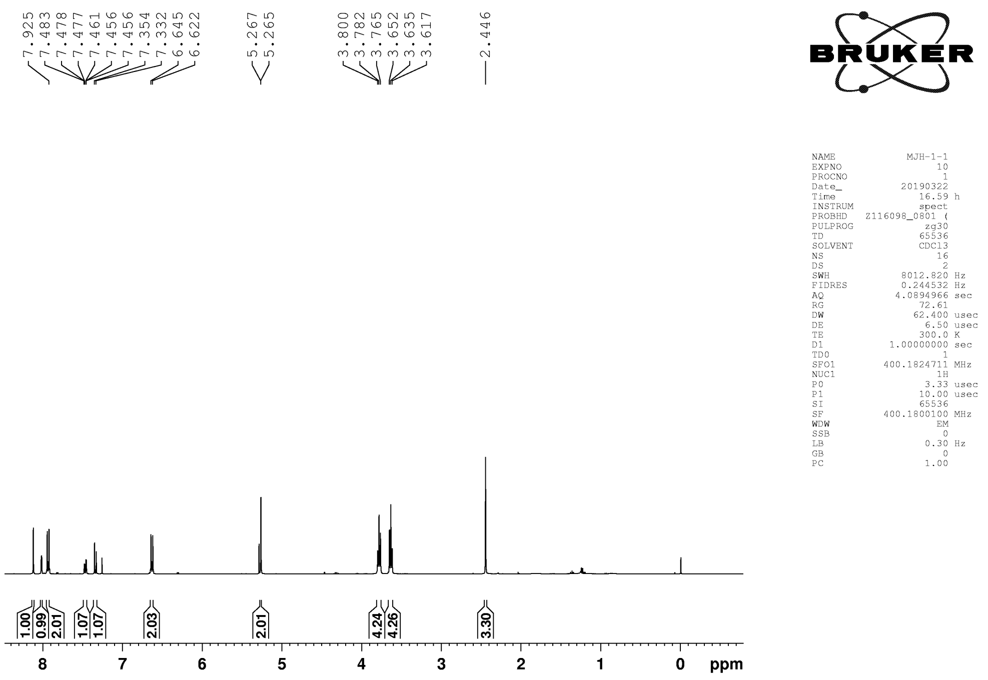

Figure S1. ^{1}H NMR of compound **17a**

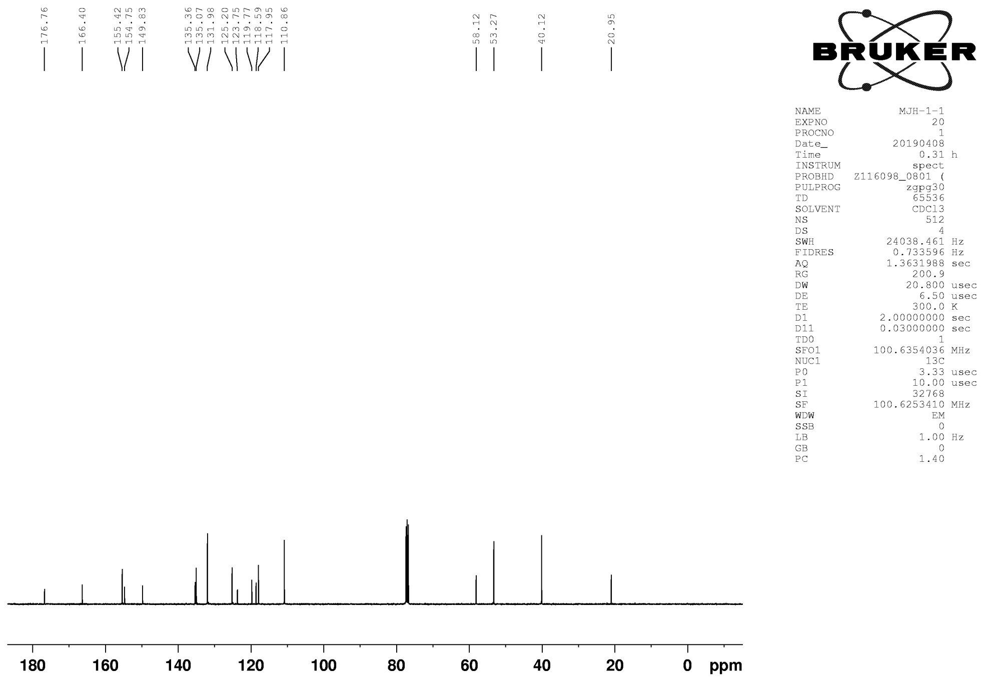

Figure S2. ^{13}C NMR of compound **17a**

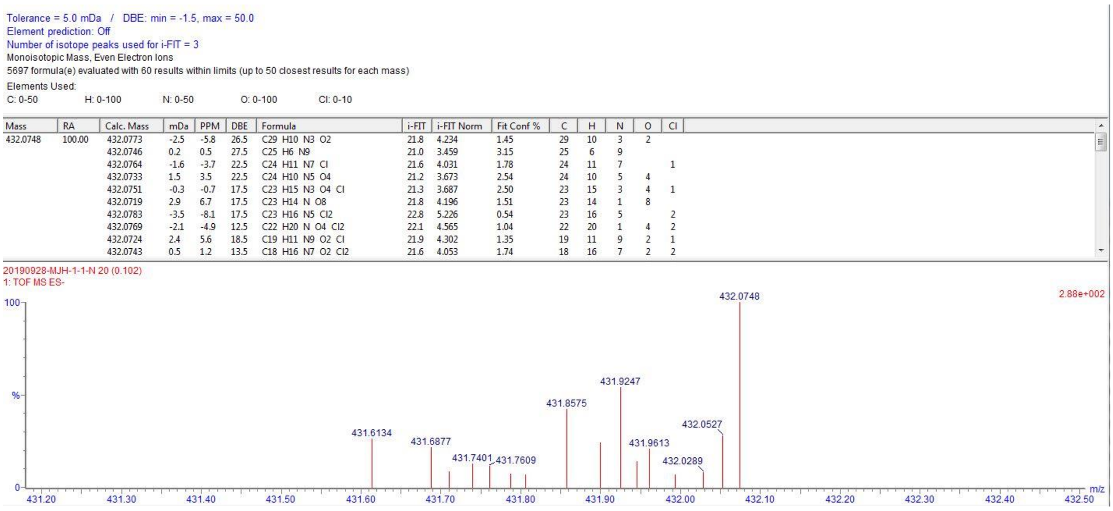

Mass	RA	Calc. Mass	mDa	PPM	DBE	Formula	i-FIT	i-FIT Norm	Fit Conf %	C	H	N	O	Cl
432.0748	100.00	432.0773	-2.5	-5.8	26.5	C29 H10 N3 O2	21.8	4.234	1.45	29	10	3	2	
		432.0746	0.2	0.5	27.5	C25 H6 N9	21.0	3.459	3.15	25	6	9		
		432.0764	-1.6	-3.7	22.5	C24 H11 N7 Cl	21.6	4.031	1.78	24	11	7		1
		432.0733	1.5	3.5	22.5	C24 H10 N5 O4	21.2	3.673	2.54	24	10	5	4	
		432.0751	-0.3	-0.7	17.5	C23 H15 N3 O4 Cl	21.3	3.687	2.50	23	15	3	4	1
		432.0719	2.9	6.7	17.5	C23 H14 N O8	21.8	4.196	1.51	23	14	1	8	
		432.0783	-3.5	-8.1	17.5	C23 H16 N5 Cl2	22.8	5.226	0.54	23	16	5		2
		432.0769	-2.1	-4.9	12.5	C22 H20 N O4 Cl2	22.1	4.565	1.04	22	20	1	4	2
		432.0724	2.4	5.6	18.5	C19 H11 N9 O2 Cl	21.9	4.302	1.35	19	11	9	2	1
		432.0743	0.5	1.2	13.5	C18 H16 N7 O2 Cl2	21.6	4.053	1.74	18	16	7	2	2

Figure S3. HR-MS of compound **17a**

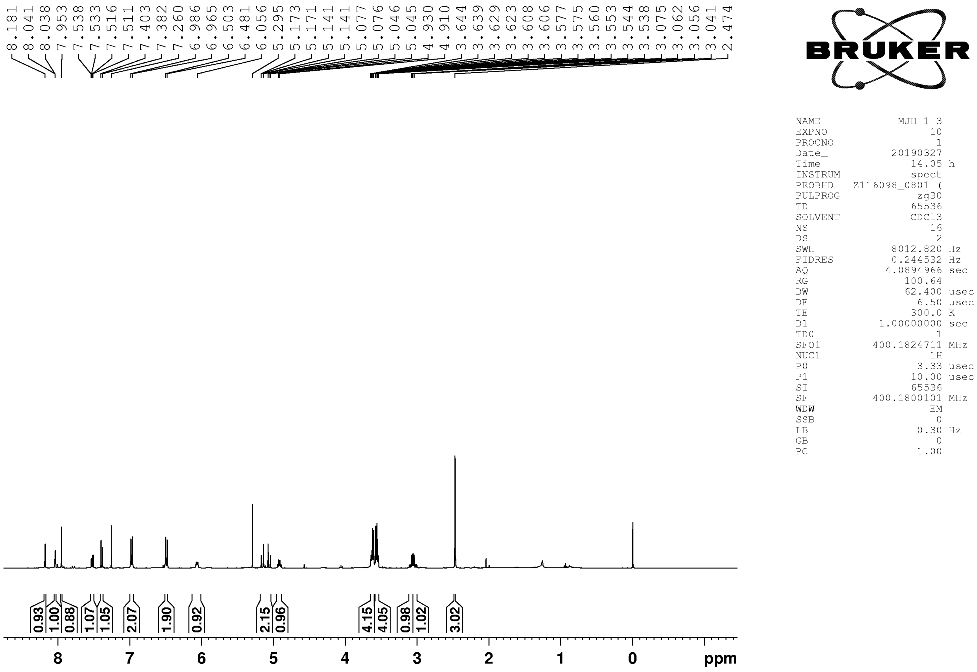

Figure S4. ^{1}H NMR of compound **17b**

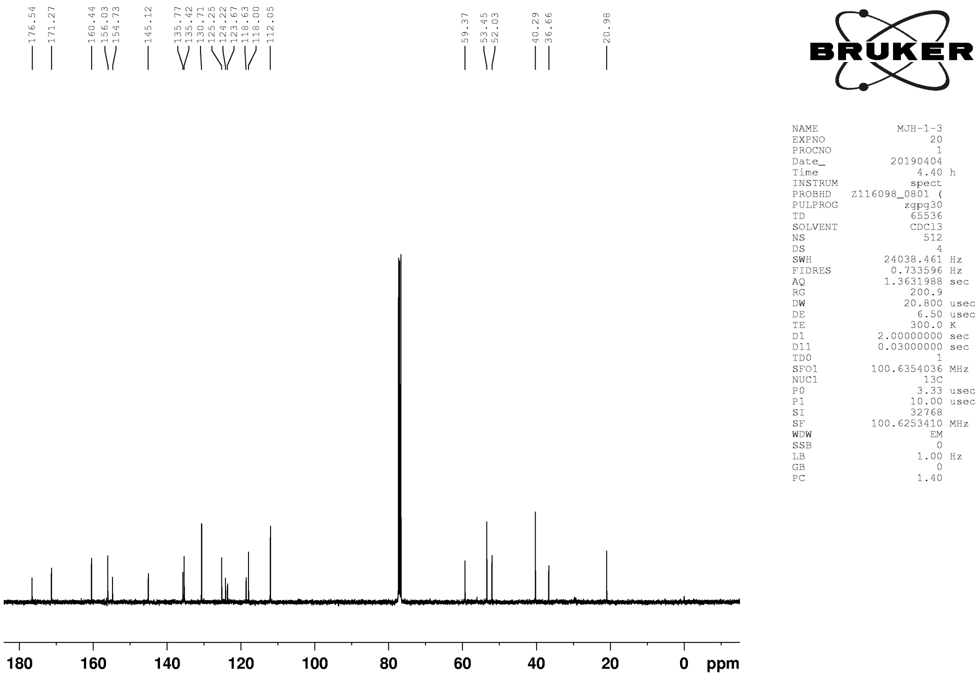

Figure S5. ^{13}C NMR of compound **17b**

Tolerance = 5.0 mDa / DBE: min = -1.5, max = 50.0
Element prediction: Off
Number of isotope peaks used for i-FIT = 3
Monoisotopic Mass, Even Electron Ions
9568 formula(e) evaluated with 85 results within limits (up to 20 closest results for each mass)
Elements Used:
C: 0-50 H: 0-100 N: 0-50 O: 0-100 Cl: 0-10

Mass	RA	Calc. Mass	mDa	PPM	DBE	Formula	i-FIT	i-FIT Norm	Fit Conf %	C	H	N	O	Cl
503.1129	100.00	503.1131	-0.2	-0.4	22.5	C31 H19 O7	184.9	2.876	5.64	31	19		7	
		503.1117	1.2	2.4	28.5	C28 H11 N10 O	185.1	3.081	4.59	28	11	10	1	
		503.1136	-0.7	-1.4	23.5	C27 H16 N8 O Cl	185.1	3.100	4.50	27	16	8	1	1
		503.1122	0.7	1.4	18.5	C26 H20 N4 O5 Cl	185.1	3.096	4.52	26	20	4	5	1
		503.1141	-1.2	-2.4	13.5	C25 H25 N2 O5 Cl2	185.5	3.485	3.07	25	25	2	5	2
		503.1132	-0.3	-0.6	9.5	C20 H26 N6 O3 Cl3	185.5	3.503	3.01	20	26	6	3	3
		503.1119	1.0	2.0	4.5	C19 H30 N2 O7 Cl3	185.6	3.665	2.56	19	30	2	7	3
		503.1137	-0.8	-1.6	-0.5	C18 H35 O7 Cl4	185.7	3.722	2.42	18	35		7	4
		503.1136	-0.7	-1.4	15.5	C16 H15 N12 O8	184.6	2.617	7.30	16	15	12	8	
		503.1123	0.6	1.2	5.5	C15 H27 N10 O Cl4	185.7	3.681	2.52	15	27	10	1	4
		503.1122	0.7	1.4	10.5	C15 H19 N8 O12	184.5	2.529	7.97	15	19	8	12	

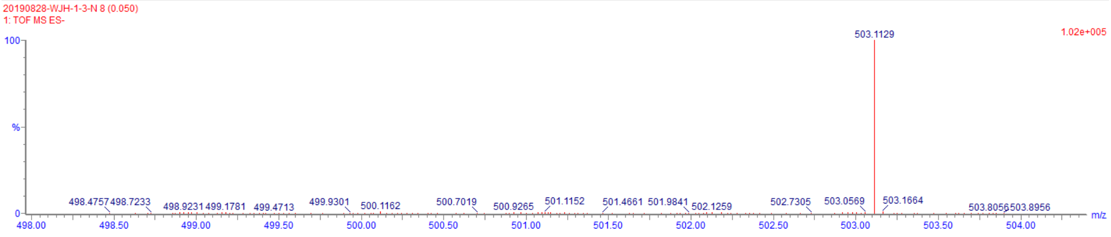

Figure S6. HR-MS of compound **17b**

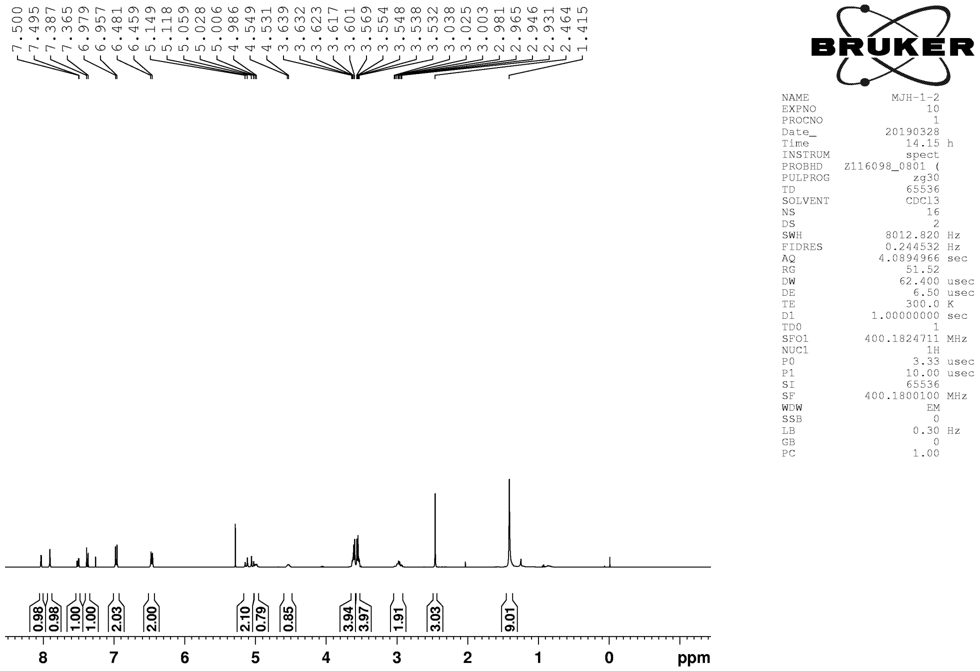

Figure S7. ^{1}H NMR of compound **17c**

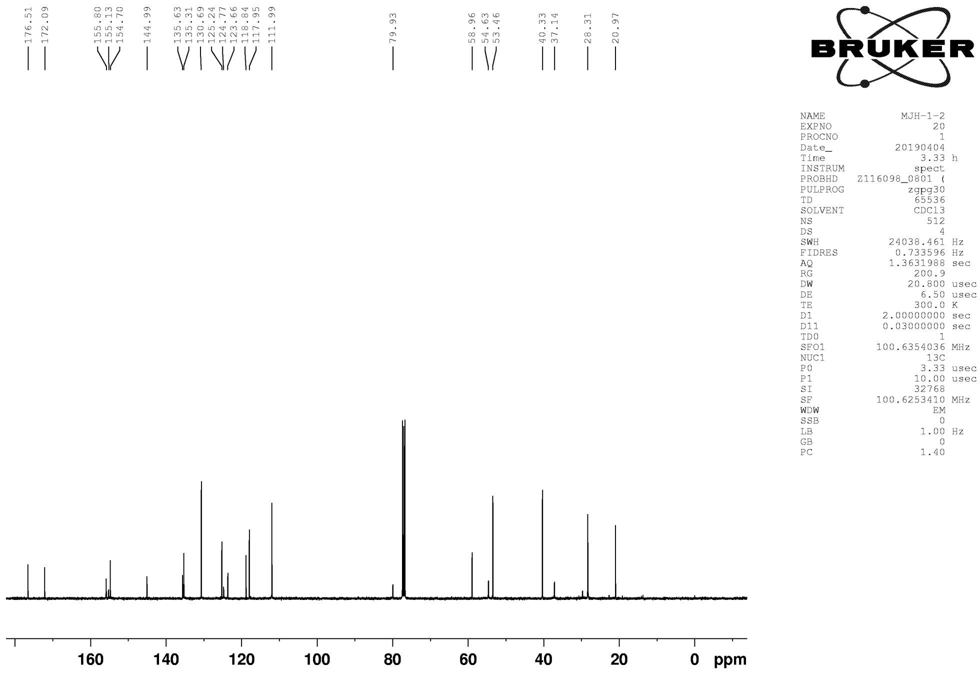

Figure S8. ^{13}C NMR of compound **17c**

Tolerance = 5.0 mDa / DBE: min = -1.5, max = 50.0
Element prediction: Off
Number of isotope peaks used for i-FIT = 3
Monoisotopic Mass, Even Electron Ions
15051 formula(e) evaluated with 109 results within limits (up to 10 closest results for each mass)
Elements Used:
C: 0-50 H: 0-100 N: 0-50 O: 0-100 Cl: 0-10

Mass	RA	Calc. Mass	mDa	PPM	DBE	Formula	i-FIT	i-FIT Norm	Fit Conf %	C	H	N	O	Cl
575.1713	100.00	575.1716	-0.3	-0.5	13.5	C29 H33 N2 O6 Cl2	616.8	0.000	100.00	29	33	2	6	2
		575.1716	-0.3	-0.5	5.5	C18 H32 N6 O13 Cl	627.8	11.030	0.00	18	32	6	13	1
		575.1712	0.1	0.2	-0.5	C22 H43 O8 Cl4	627.9	11.088	0.00	22	43		8	4
		575.1717	-0.4	-0.7	0.5	C18 H40 N8 O2 Cl5	628.6	11.793	0.00	18	40	8	2	5
		575.1711	0.2	0.3	23.5	C31 H24 N8 O2 Cl	628.9	12.079	0.00	31	24	8	2	1
		575.1716	-0.3	-0.5	16.5	C16 H20 N20 O3 Cl	629.5	12.741	0.00	16	20	20	3	1
		575.1712	0.1	0.2	2.5	C9 H30 N18 O5 Cl3	630.0	13.249	0.00	9	30	18	5	3
		575.1716	-0.3	-0.5	19.5	C3 H7 N38	636.8	20.062	0.00	3	7	38		
		575.1711	0.2	0.3	15.5	C20 H23 N12 O9	638.7	21.909	0.00	20	23	12	9	
		575.1716	-0.3	-0.5	8.5	C5 H19 N24 O10	639.0	22.234	0.00	5	19	24	10	

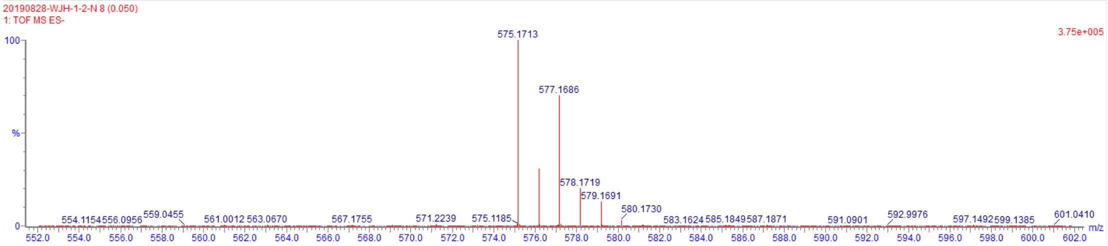

Figure S9. HR-MS of compound **17c**

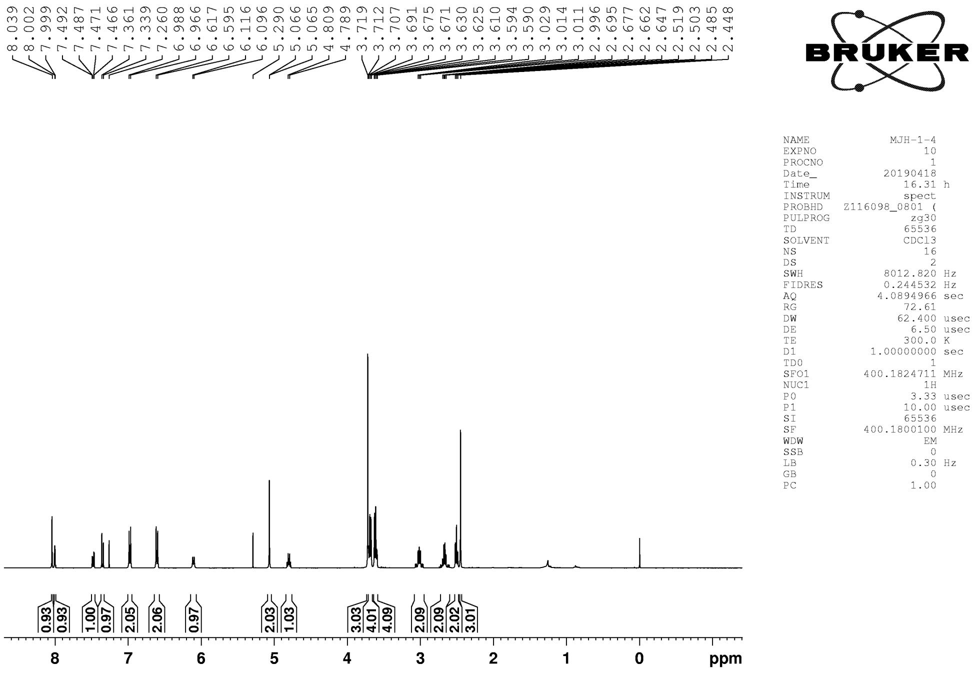

Figure S10. ^{1}H NMR of compound **17d**

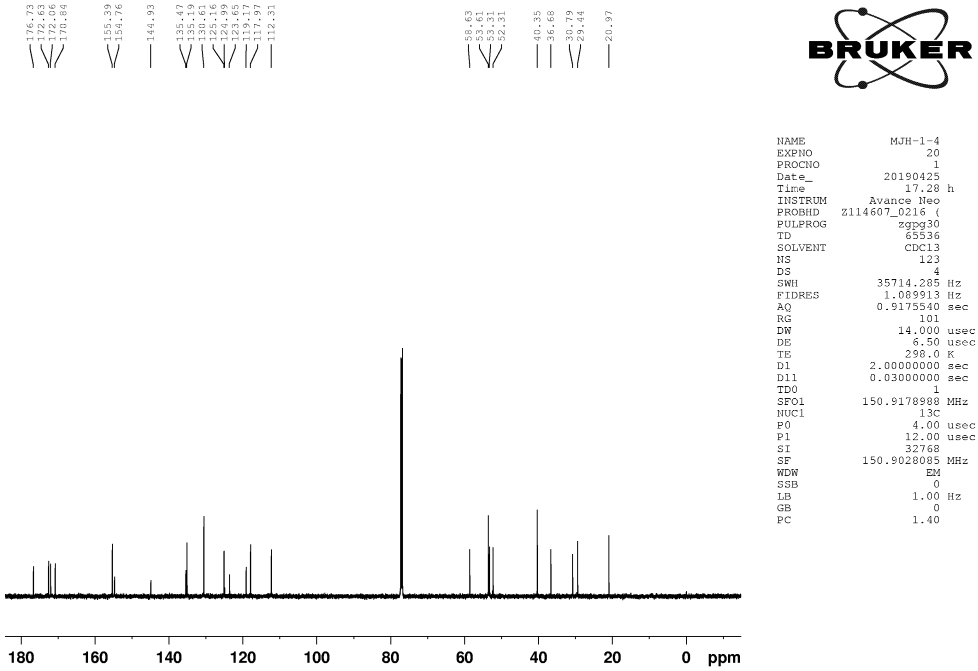

Figure S11. ^{13}C NMR of compound **17d**

Tolerance = 5.0 mDa / DBE: min = -1.5, max = 50.0
Element prediction: Off
Number of isotope peaks used for i-FIT = 3
Monoisotopic Mass, Even Electron Ions
16338 formula(e) evaluated with 126 results within limits (up to 10 closest results for each mass)
Elements Used:
C: 0-50 H: 0-100 N: 0-50 O: 0-100 Cl: 0-10

Mass	RA	Calc. Mass	mDa	PPM	DBE	Formula	i-FIT	i-FIT Norm	Fit Conf %	C	H	N	O	Cl
589.1506	100.00	589.1509	-0.3	-0.5	-1.5	C7 H29 N10 O21	185.2	1.595	20.30	7	29	10	21	
		589.1509	-0.3	-0.5	9.5	C5 H17 N24 O11	185.4	1.717	17.97	5	17	24	11	
		589.1504	0.2	0.3	16.5	C20 H21 N12 O10	185.8	2.186	11.24	20	21	12	10	
		589.1503	0.3	0.5	27.5	C18 H9 N26	185.9	2.255	10.49	18	9	26		
		589.1508	-0.2	-0.3	17.5	C16 H18 N20 O4 Cl	186.1	2.463	8.52	16	18	20	4	1
		589.1509	-0.3	-0.5	6.5	C18 H30 N6 O14 Cl	186.1	2.472	8.44	18	30	6	14	1
		589.1503	0.3	0.5	24.5	C31 H22 N8 O3 Cl	186.2	2.581	7.57	31	22	8	3	1
		589.1508	-0.2	-0.3	14.5	C29 H31 N2 O7 Cl2	186.4	2.807	6.04	29	31	2	7	2
		589.1505	0.1	0.2	3.5	C9 H28 N18 O6 Cl3	186.6	2.993	5.01	9	28	18	6	3
		589.1505	0.1	0.2	0.5	C22 H41 O9 Cl4	186.8	3.116	4.44	22	41		9	4

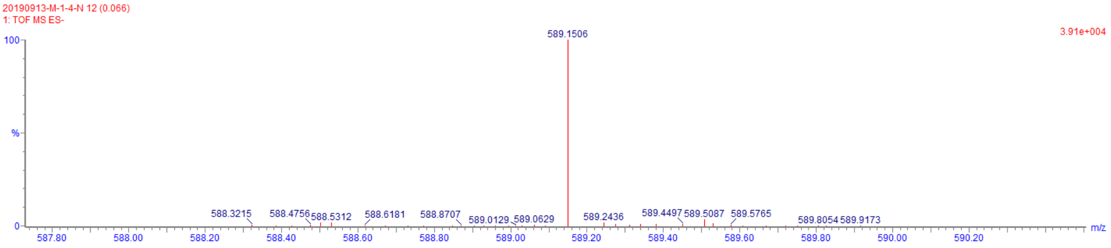

Figure S12. HR-MS of compound **17d**

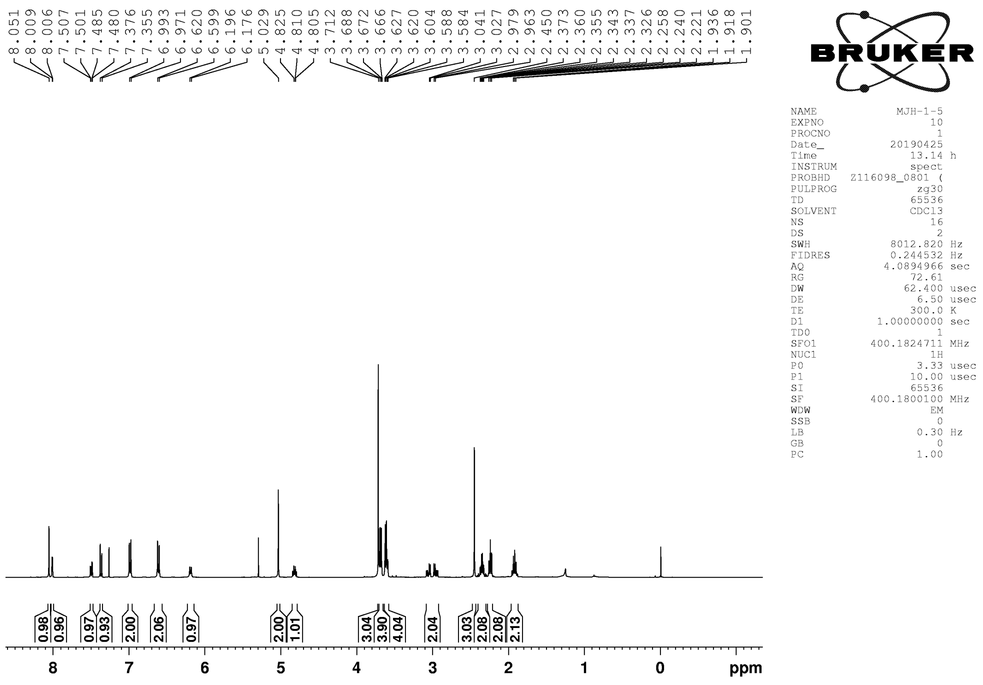

Figure S13. ^{1}H NMR of compound **17e**

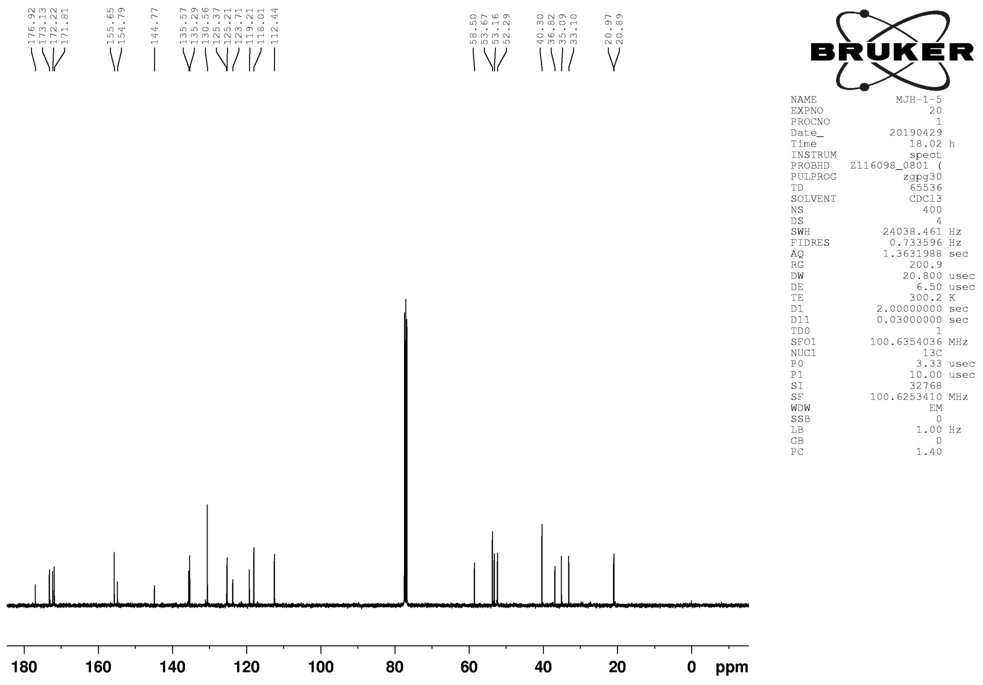

Figure S14. ^{13}C NMR of compound **17e**

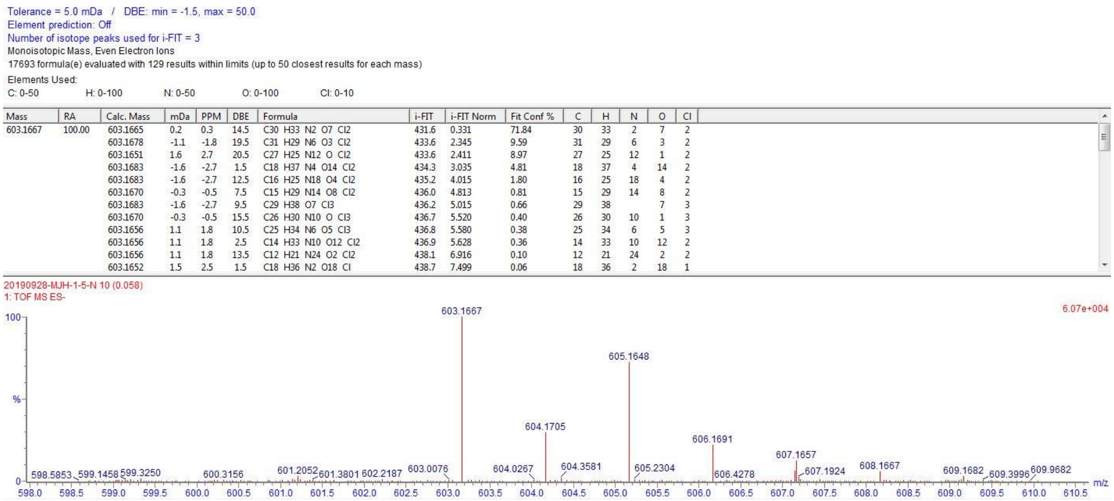

Tolerance = 5.0 mDa / DBE: min = -1.5, max = 50.0
Element prediction: Off
Number of isotope peaks used for i-FIT = 3
Monoisotopic Mass, Even Electron Ions
17693 formula(e) evaluated with 129 results within limits (up to 50 closest results for each mass)
Elements Used:
C: 0-50 H: 0-100 N: 0-50 O: 0-100 Cl: 0-10

Mass	RA	Calc. Mass	mDa	PPM	DBE	Formula	i-FIT	i-FIT Norm	Fit Conf %	C	H	N	O	Cl
603.1667	100.00	603.1665	0.2	0.3	14.5	C30 H33 N2 O7 Cl2	431.6	0.331	71.84	30	33	2	7	2
		603.1678	-1.1	-1.8	19.5	C31 H29 N6 O3 Cl2	433.6	2.345	9.59	31	29	6	3	2
		603.1651	1.6	2.7	20.5	C27 H25 N12 O Cl2	433.6	2.411	8.97	27	25	12	1	2
		603.1683	-1.6	-2.7	1.5	C18 H37 N4 O14 Cl2	434.3	3.035	4.81	18	37	4	14	2
		603.1683	-1.6	-2.7	12.5	C16 H25 N18 O4 Cl2	435.2	4.015	1.80	16	25	18	4	2
		603.1670	-0.3	-0.5	7.5	C15 H29 N14 O8 Cl2	436.0	4.813	0.81	15	29	14	8	2
		603.1683	-1.6	-2.7	9.5	C29 H38 O7 Cl3	436.2	5.015	0.66	29	38		7	3
		603.1670	-0.3	-0.5	15.5	C26 H30 N10 O Cl3	436.7	5.520	0.40	26	30	10	1	3
		603.1656	1.1	1.8	10.5	C25 H34 N6 O5 Cl3	436.8	5.580	0.38	25	34	6	5	3
		603.1656	1.1	1.8	2.5	C14 H33 N10 O12 Cl2	436.9	5.628	0.36	14	33	10	12	2
		603.1656	1.1	1.8	13.5	C12 H21 N24 O2 Cl2	438.1	6.916	0.10	12	21	24	2	2
		603.1652	1.5	2.5	1.5	C18 H36 N2 O18 Cl	438.7	7.499	0.06	18	36	2	18	1

Figure S15. HR-MS of compound **17e**

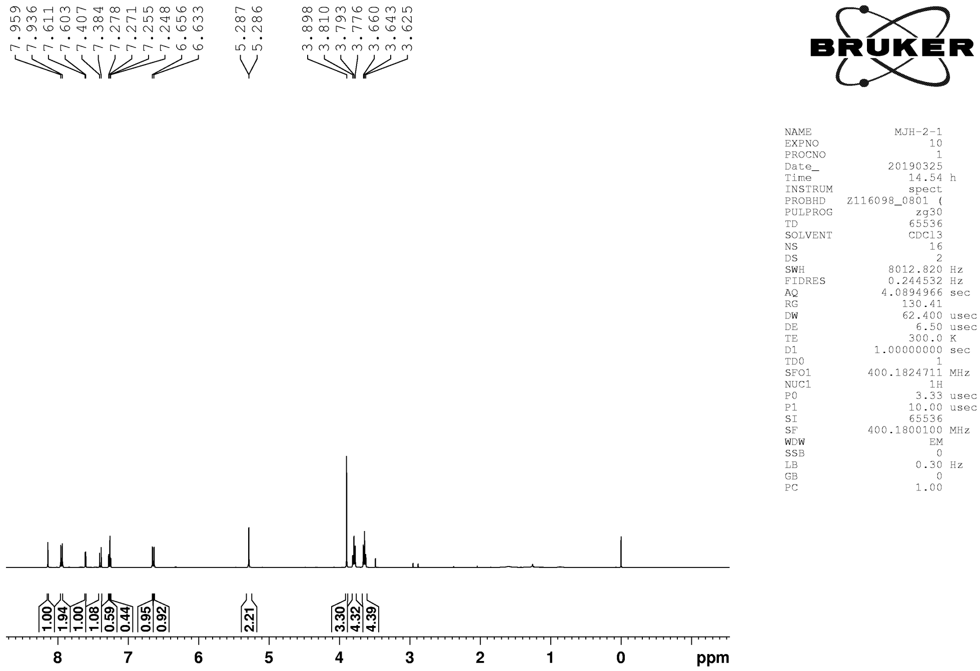

Figure S16. ^{1}H NMR of compound **18a**

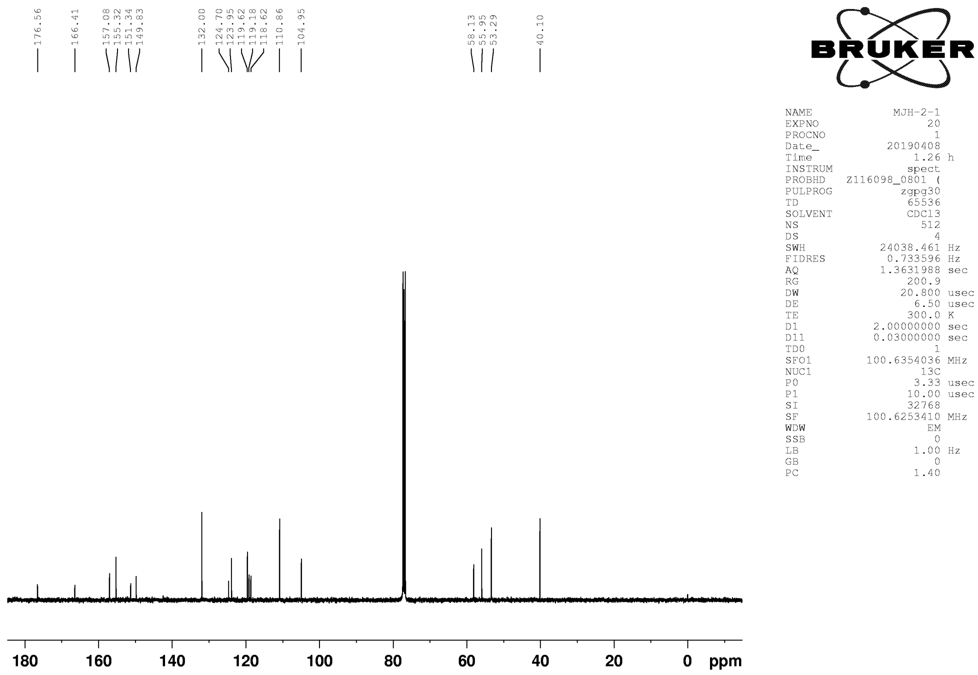

Figure S17. ^{13}C NMR of compound **18a**

Tolerance = 5.0 mDa / DBE: min = -1.5, max = 50.0
Element prediction: Off
Number of isotope peaks used for i-FIT = 3
Monoisotopic Mass, Even Electron Ions
6443 formula(e) evaluated with 68 results within limits (up to 10 closest results for each mass)
Elements Used:
C: 0-50 H: 0-100 N: 0-50 O: 0-100 Cl: 0-10

Mass	RA	Calc. Mass	mDa	PPM	DBE	Formula	i-FIT	i-FIT Norm	Fit Conf %	C	H	N	O	Cl
448.0714	100.00	448.0714	0.0	0.0	14.5	C13 H10 N11 O8	93.7	1.513	22.03	13	10	11	8	
		448.0719	-0.5	-1.1	15.5	C9 H7 N19 O2 Cl	94.3	2.092	12.34	9	7	19	2	1
		448.0719	-0.5	-1.1	4.5	C11 H19 N5 O12 Cl	94.3	2.113	12.09	11	19	5	12	1
		448.0714	0.0	0.0	22.5	C24 H11 N7 O Cl	94.4	2.183	11.27	24	11	7	1	1
		448.0710	0.4	0.9	11.5	C4 H8 N23 Cl2	94.7	2.504	8.17	4	8	23		2
		448.0719	-0.5	-1.1	12.5	C22 H20 N O5 Cl2	94.7	2.517	8.07	22	20	1	5	2
		448.0710	0.4	0.9	0.5	C6 H20 N9 O10 Cl2	94.8	2.529	7.98	6	20	9	10	2
		448.0710	0.4	0.9	8.5	C17 H21 N5 O3 Cl3	95.0	2.727	6.54	17	21	5	3	3
		448.0715	-0.1	-0.2	1.5	C2 H17 N17 O4 Cl3	95.0	2.734	6.50	2	17	17	4	3
		448.0720	-0.6	-1.3	-0.5	C11 H27 N7 O Cl5	95.2	2.993	5.01	11	27	7	1	5

20190913-M-2-1-N 152 (0.613)
1: TOF MS ES-

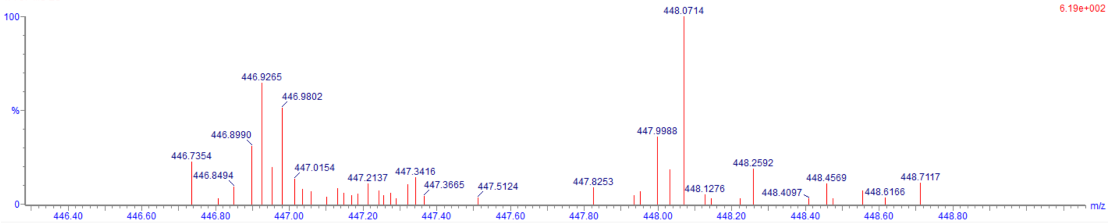

Figure S18. HR-MS of compound **18a**

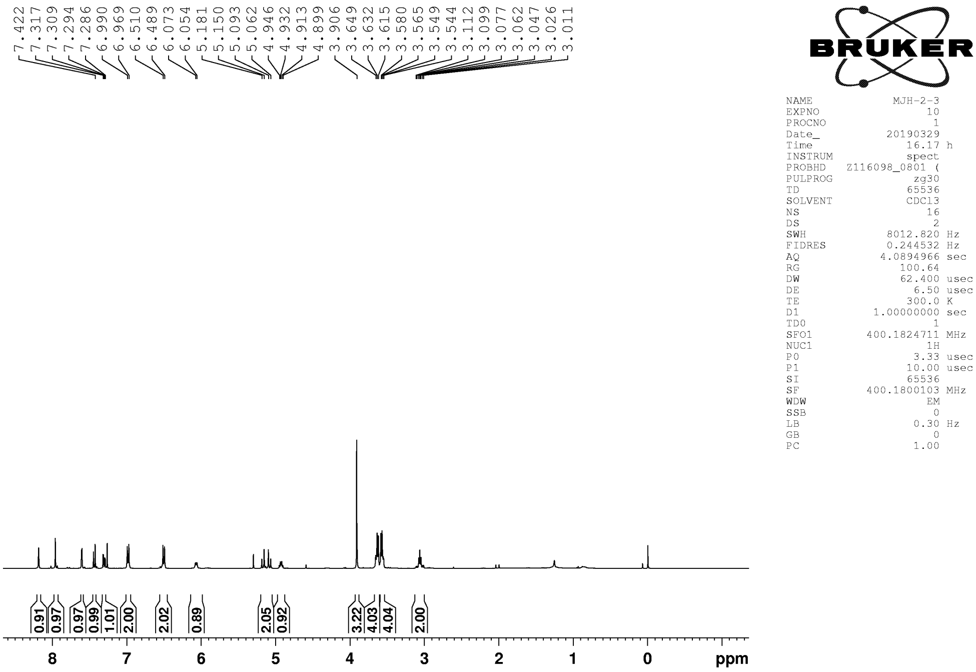

Figure S19. ^{1}H NMR of compound **18b**

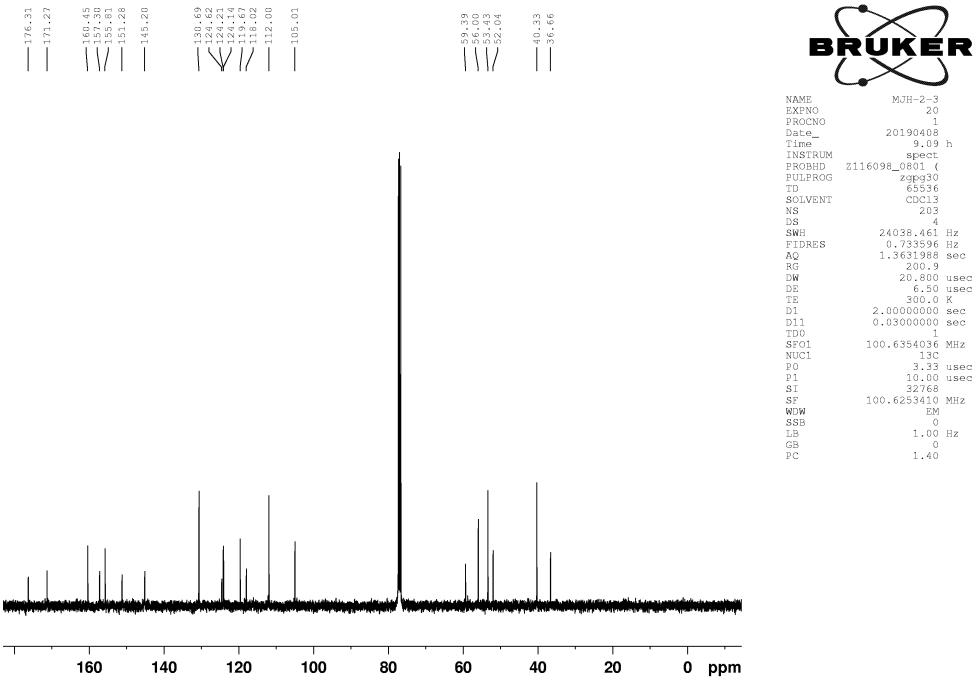

Figure S20. ^{13}C NMR of compound **18b**

Tolerance = 5.0 mDa / DBE: min = -1.5, max = 50.0
Element prediction: Off
Number of isotope peaks used for i-FIT = 3
Monoisotopic Mass, Even Electron Ions
10642 formula(e) evaluated with 100 results within limits (up to 10 closest results for each mass)

Elements Used:
C: 0-50 H: 0-100 N: 0-50 O: 0-100 Cl: 0-10

Mass	RA	Calc. Mass	mDa	PPM	DBE	Formula	i-FIT	i-FIT Norm	Fit Conf %	C	H	N	O	Cl
519.1094	100.00	519.1090	0.4	0.8	13.5	C25 H25 N2 O6 Cl2	490.9	0.010	98.97	25	25	2	6	2
		519.1094	0.0	0.0	14.5	C21 H22 N10 Cl3	496.2	5.280	0.51	21	22	10		3
		519.1095	-0.1	-0.2	6.5	C10 H21 N14 O7 Cl2	496.5	5.568	0.38	10	21	14	7	2
		519.1090	0.4	0.8	5.5	C14 H24 N6 O13 Cl	497.6	6.740	0.12	14	24	6	13	1
		519.1091	0.3	0.6	0.5	C14 H32 N8 O2 Cl5	499.9	8.992	0.01	14	32	8	2	5
		519.1090	0.4	0.8	16.5	C12 H12 N20 O3 Cl	500.8	9.882	0.01	12	12	20	3	1
		519.1098	-0.4	-0.8	20.5	C17 H11 N16 O5	509.6	18.722	0.00	17	11	16	5	
		519.1098	-0.4	-0.8	9.5	C19 H23 N2 O15	509.9	18.973	0.00	19	23	2	15	
		519.1093	0.1	0.2	27.5	C32 H15 N4 O4	510.4	19.517	0.00	32	15	4	4	
		519.1090	0.4	0.8	8.5	C H11 N24 O10	510.9	19.964	0.00	1	11	24	10	

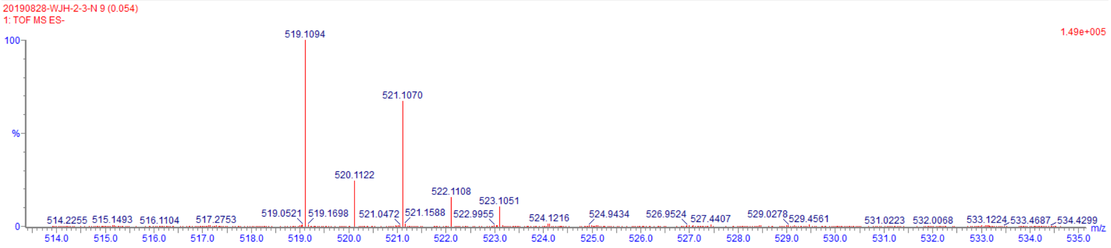

Figure S21. HR-MS of compound **18b**

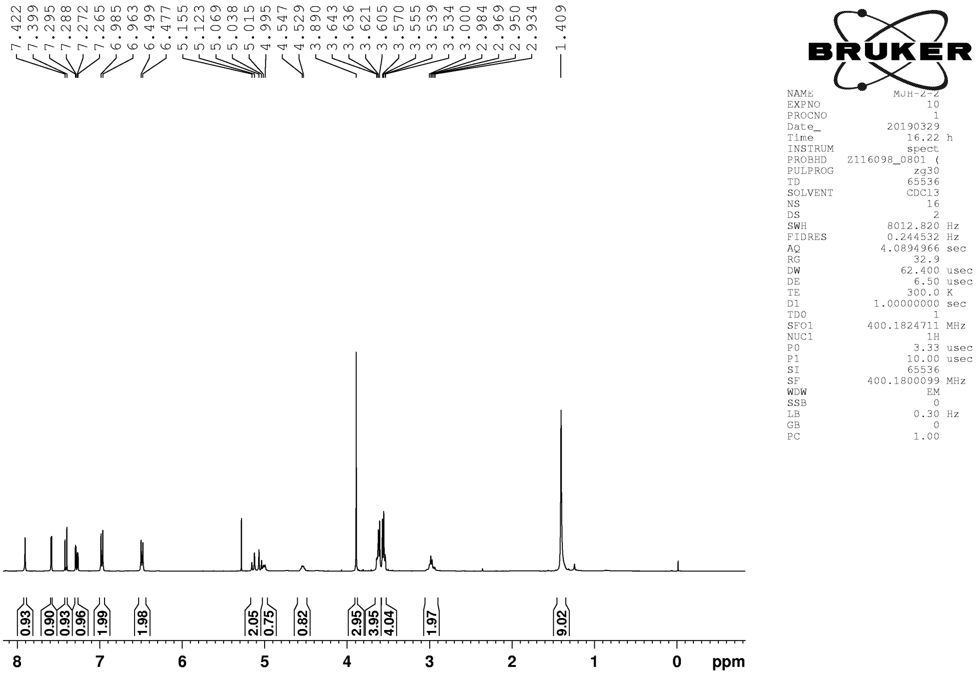

Figure S22. ^{1}H NMR of compound **18c**

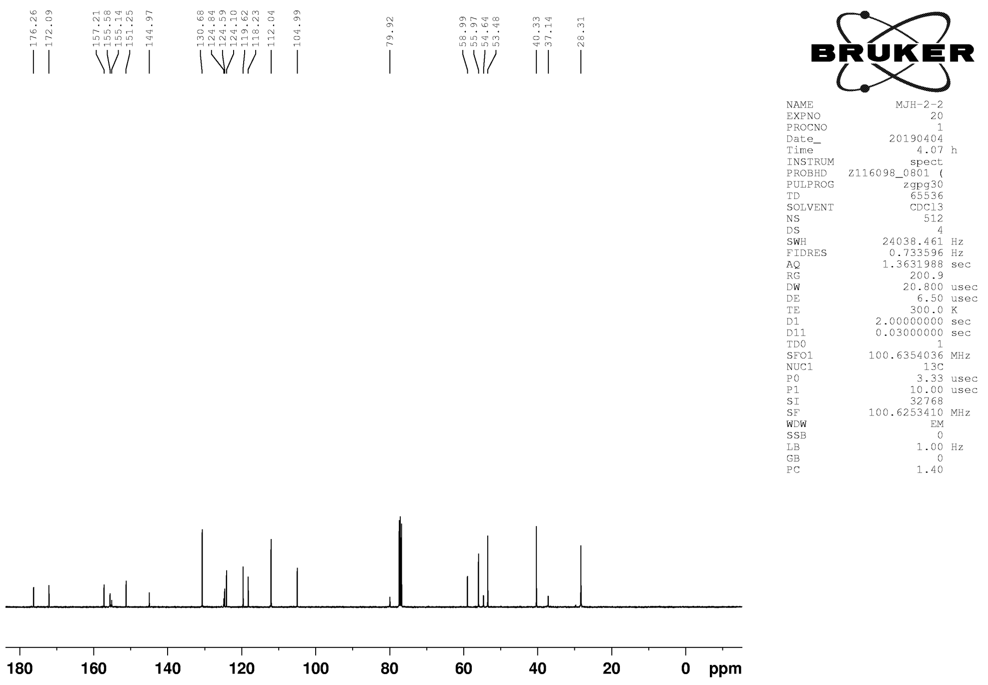

Figure S23. ^{13}C NMR of compound **18c**

Tolerance = 5.0 mDa / DBE: min = -1.5, max = 50.0
Element prediction: Off
Number of isotope peaks used for i-FIT = 3
Monoisotopic Mass, Even Electron Ions
16525 formula(e) evaluated with 120 results within limits (up to 50 closest results for each mass)
Elements Used:
C: 0-50 H: 0-100 N: 0-50 O: 0-100 Cl: 0-10

Mass	RA	Calc. Mass	mDa	PPM	DBE	Formula	i-FIT	i-FIT Norm	Fit Conf %	C	H	N	O	Cl
591.1677	100.00	591.1665	1.2	2.0	13.5	C29 H33 N2 O7 Cl2	479.7	0.342	71.06	29	33	2	7	2
		591.1697	-2.0	-3.4	16.5	C16 H21 N22 Cl2	481.6	2.162	11.51	16	21	22		2
		591.1678	-0.1	-0.2	18.5	C30 H29 N6 O3 Cl2	481.8	2.394	9.13	30	29	6	3	2
		591.1683	-0.6	-1.0	11.5	C15 H25 N18 O4 Cl2	482.5	3.054	4.72	15	25	18	4	2
		591.1670	0.7	1.2	6.5	C14 H29 N14 O8 Cl2	483.6	4.219	1.47	14	29	14	8	2
		591.1697	-2.0	-3.4	5.5	C18 H33 N8 O10 Cl2	483.8	4.378	1.26	18	33	8	10	2
		591.1683	-0.6	-1.0	0.5	C17 H37 N4 O14 Cl2	485.0	5.620	0.36	17	37	4	14	2
		591.1670	0.7	1.2	14.5	C25 H30 N10 O Cl3	485.9	6.493	0.15	25	30	10	1	3
		591.1688	-1.1	-1.9	1.5	C13 H34 N12 O8 Cl3	486.8	7.353	0.06	13	34	12	8	3
		591.1678	-0.1	-0.2	10.5	C19 H28 N10 O10 Cl	487.3	7.865	0.04	19	28	10	10	1

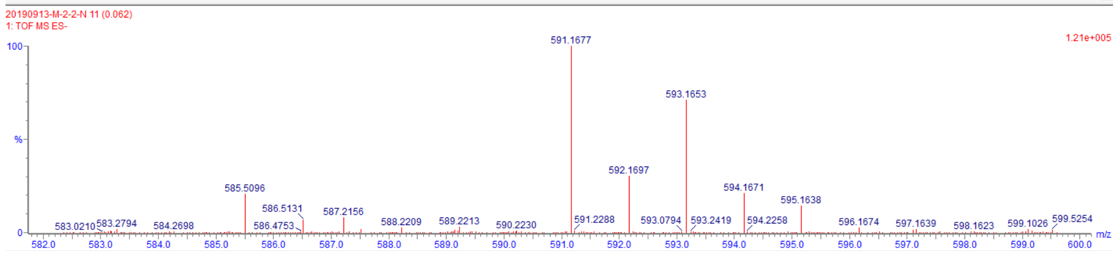

Figure S24. HR-MS of compound **18c**

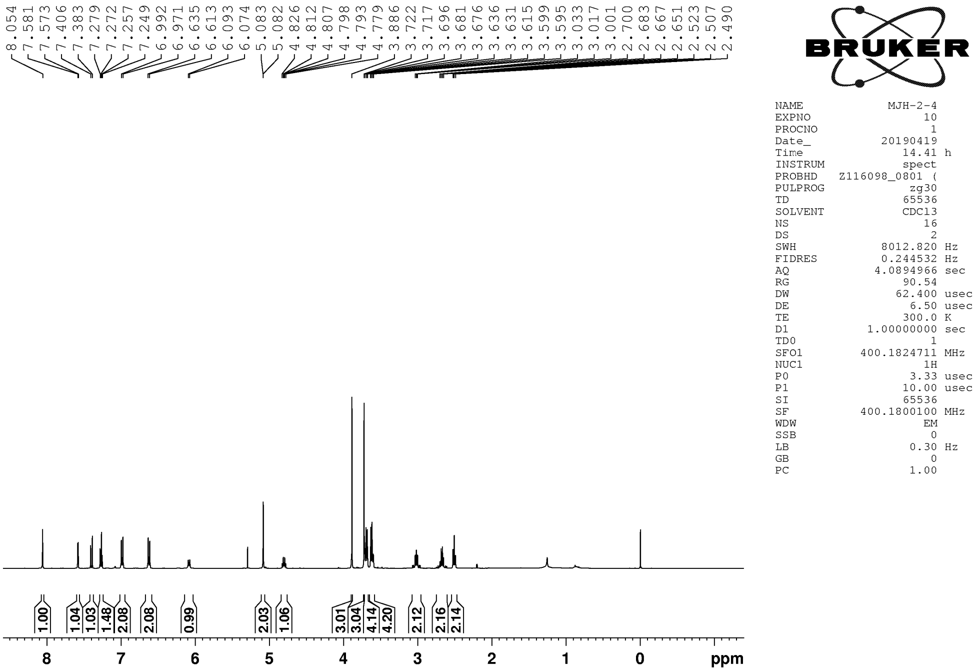

Figure S25. ^{1}H NMR of compound **18d**

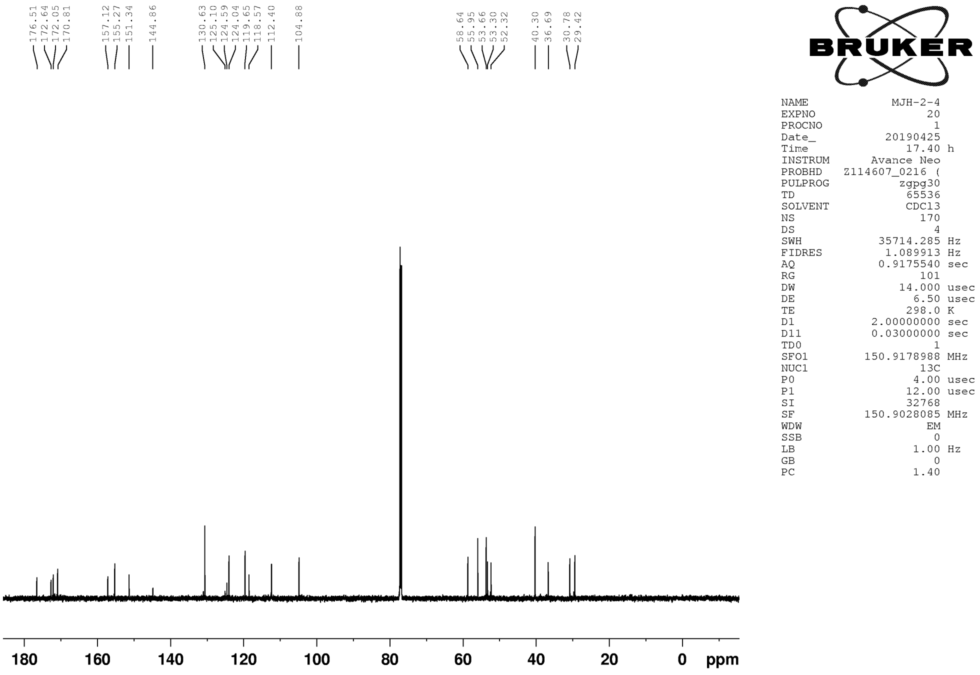

Figure S26. ^{13}C NMR of compound **18d**

Tolerance = 5.0 mDa / DBE: min = -1.5, max = 50.0
Element prediction: Off
Number of isotope peaks used for i-FIT = 3
Monoisotopic Mass, Even Electron Ions
17898 formula(e) evaluated with 140 results within limits (up to 20 closest results for each mass)
Elements Used:
C: 0-50 H: 0-100 N: 0-50 O: 0-100 Cl: 0-10

Mass	RA	Calc. Mass	mDa	PPM	DBE	Formula	i-FIT	i-FIT Norm	Fit Conf %	C	H	N	O	Cl
605.1450	100.00	605.1453	-0.3	-0.5	16.5	C20 H21 N12 O11	256.6	1.314	26.88	20	21	12	11	
		605.1448	0.2	0.3	34.5	C33 H13 N14	256.8	1.505	22.21	33	13	14		
		605.1448	0.2	0.3	23.5	C35 H25 O10	257.3	2.014	13.34	35	25		10	
		605.1453	-0.3	-0.5	27.5	C18 H9 N26 O	257.8	2.465	8.50	18	9	26	1	
		605.1453	-0.3	-0.5	24.5	C31 H22 N8 O4 Cl	258.3	2.936	5.31	31	22	8	4	1
		605.1444	0.6	1.0	1.5	C17 H34 N2 O19 Cl	258.6	3.248	3.89	17	34	2	19	1
		605.1444	0.6	1.0	20.5	C26 H23 N12 O2 Cl2	258.8	3.434	3.23	26	23	12	2	2
		605.1449	0.1	0.2	2.5	C13 H31 N10 O13 Cl2	258.9	3.617	2.69	13	31	10	13	2
		605.1449	0.1	0.2	10.5	C24 H32 N6 O6 Cl3	258.9	3.618	2.68	24	32	6	6	3
		605.1457	-0.7	-1.2	14.5	C29 H31 N2 O8 Cl2	259.1	3.769	2.31	29	31	2	8	2

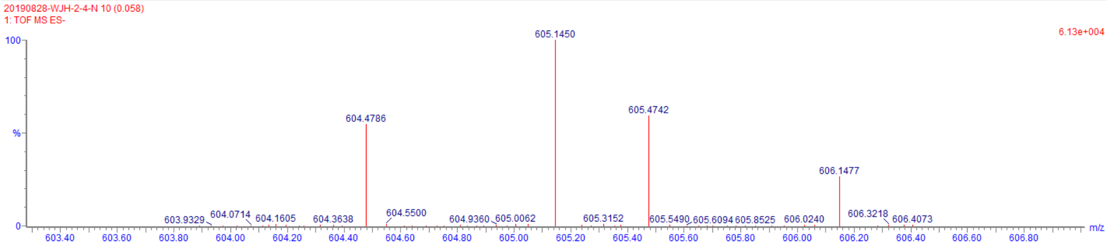

Figure S27. HR-MS of compound **18d**

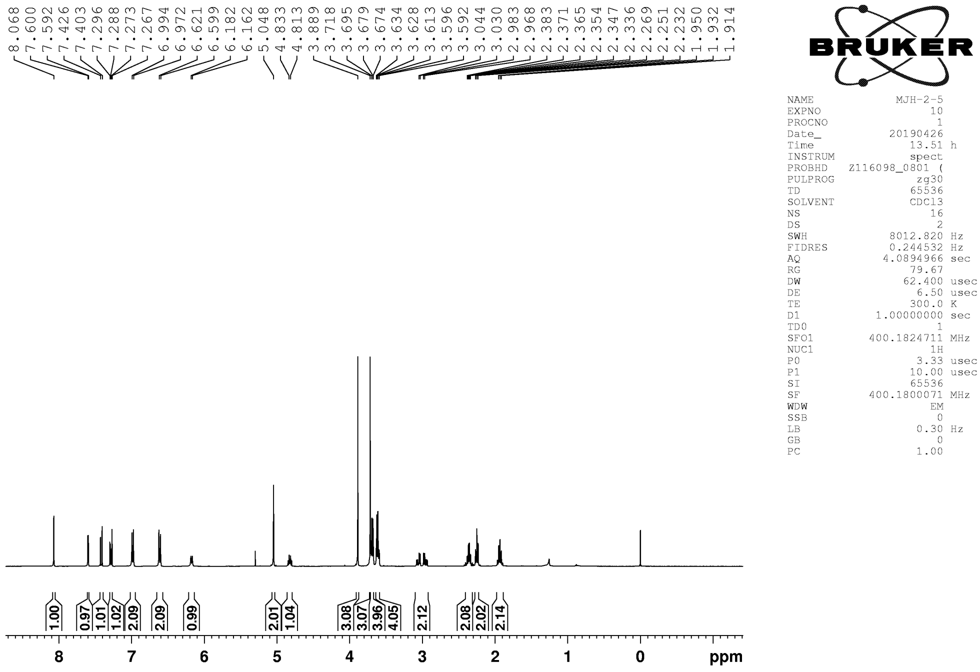

Figure S28. ^{1}H NMR of compound **18e**

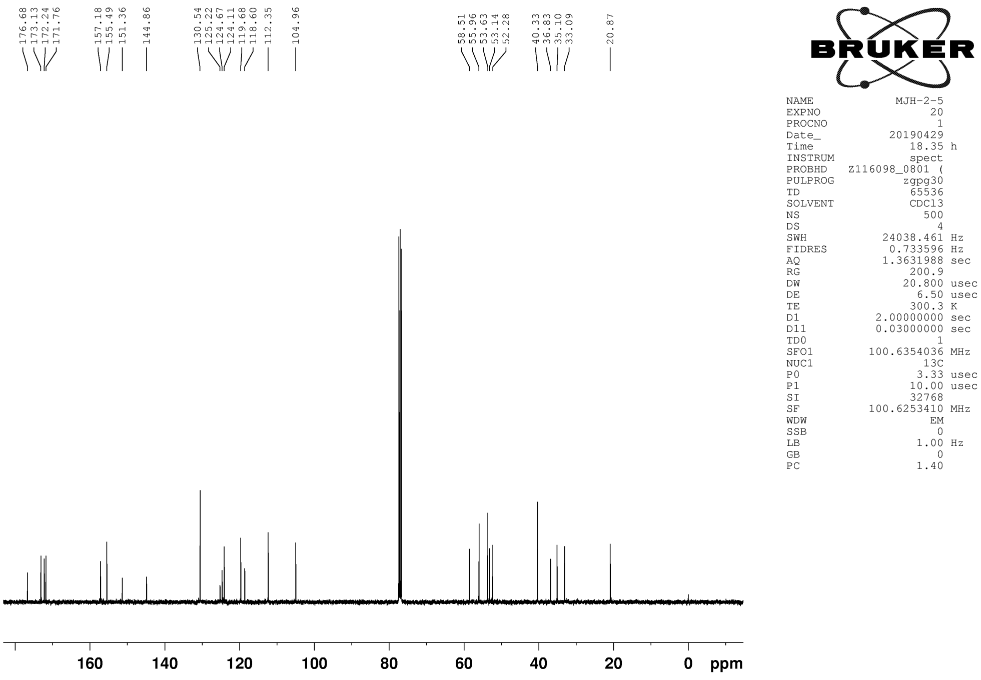

Figure S29. ^{13}C NMR of compound **18e**

Tolerance = 5.0 mDa / DBE: min = -1.5, max = 50.0
Element prediction: Off
Number of isotope peaks used for i-FIT = 3
Monoisotopic Mass, Even Electron Ions
19340 formula(e) evaluated with 141 results within limits (up to 50 closest results for each mass)
Elements Used:
C: 0-50 H: 0-100 N: 0-50 O: 0-100 Cl: 0-10

Mass	RA	Calc. Mass	mDa	PPM	DBE	Formula	i-FIT	i-FIT Norm	Fit Conf %	C	H	N	O	Cl
619.1616	100.00	619.1631	-1.5	-2.4	33.5	C38 H19 N8 O2	208.8	4.125	1.62	38	19	8	2	
		619.1618	-0.2	-0.3	28.5	C37 H23 N4 O6	208.5	3.797	2.24	37	23	4	6	
		619.1604	1.2	1.9	23.5	C36 H27 O10	208.6	3.963	1.90	36	27		10	
		619.1604	1.2	1.9	34.5	C34 H15 N14	208.7	4.005	1.82	34	15	14		
		619.1622	-0.6	-1.0	29.5	C33 H20 N12 Cl	208.6	3.952	1.92	33	20	12		1
		619.1609	0.7	1.1	24.5	C32 H24 N8 O4 Cl	208.6	3.958	1.91	32	24	8	4	1
		619.1627	-1.1	-1.8	19.5	C31 H29 N6 O4 Cl2	208.9	4.269	1.40	31	29	6	4	2
		619.1614	0.2	0.3	14.5	C30 H33 N2 O8 Cl2	208.8	4.108	1.64	30	33	2	8	2
		619.1632	-1.6	-2.6	9.5	C29 H38 O8 Cl3	209.3	4.615	0.99	29	38		8	3
		619.1602	1.4	2.3	4.5	C29 H45 O Cl6	209.5	4.849	0.78	29	45		1	6

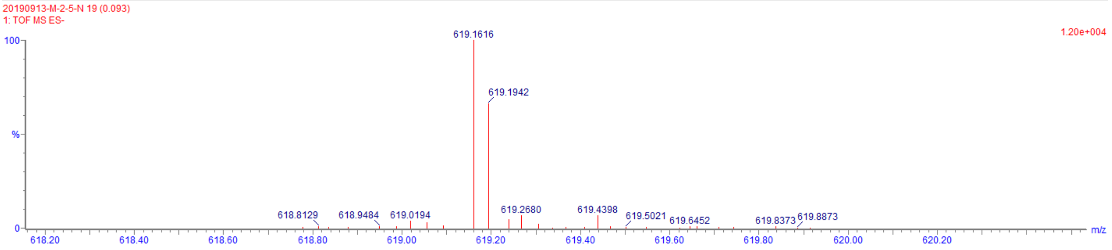

Figure S30. HR-MS of compound **18e**

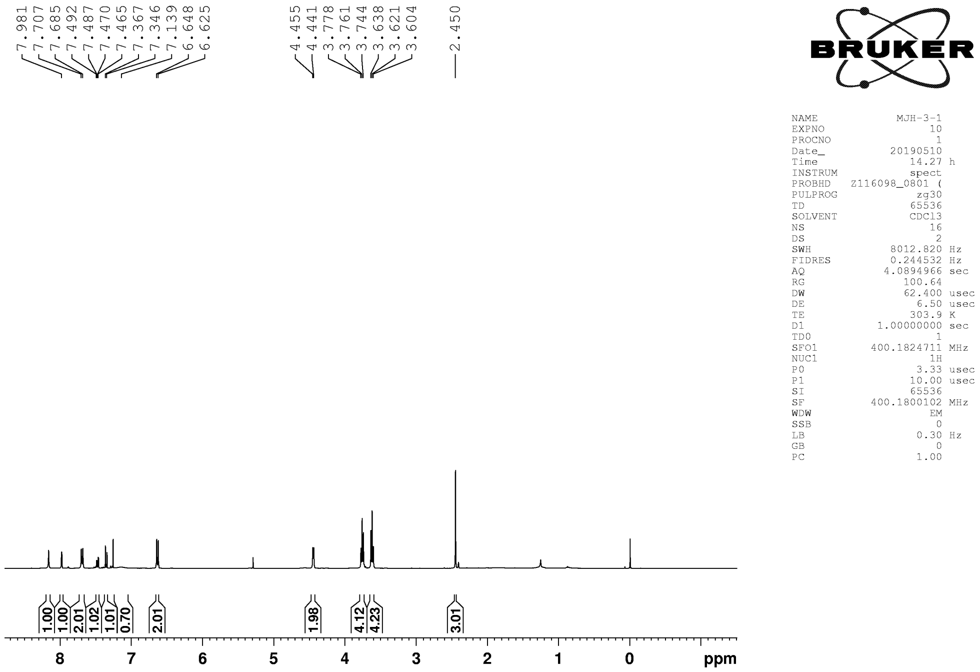

Figure S31. ^{1}H NMR of compound **21a**

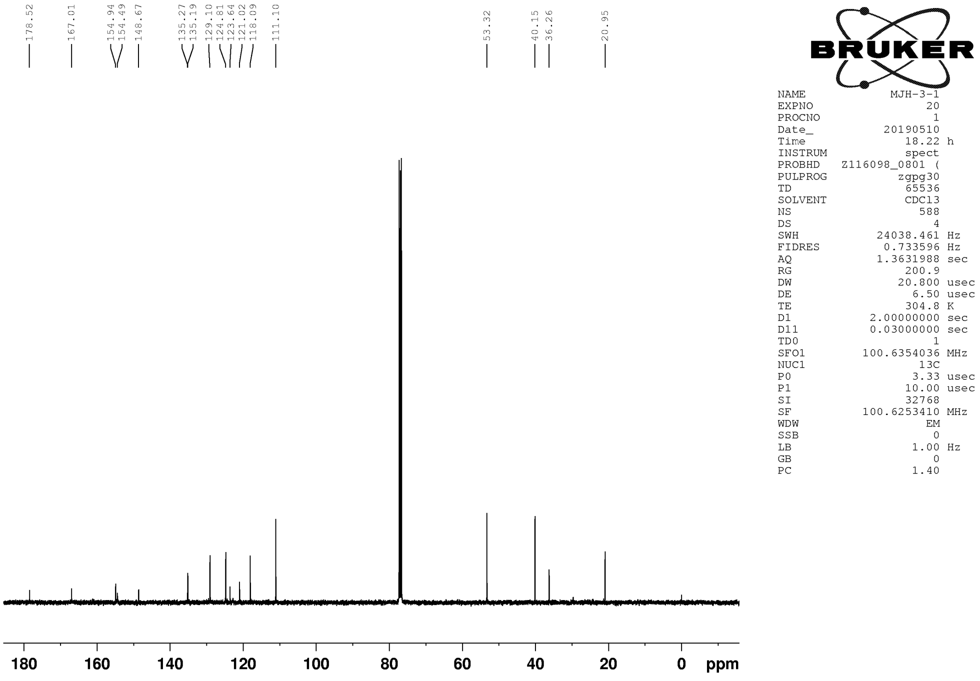

Figure S32. ^{13}C NMR of compound **21a**

Tolerance = 5.0 mDa / DBE: min = -1.5, max = 50.0
Element prediction: Off
Number of isotope peaks used for i-FIT = 3
Monoisotopic Mass, Even Electron Ions
5668 formula(e) evaluated with 59 results within limits (up to 10 closest results for each mass)
Elements Used:
C: 0-50 H: 0-100 N: 0-50 O: 0-100 Cl: 0-10

Mass	RA	Calc. Mass	mDa	PPM	DBE	Formula	i-FIT	i-FIT Norm	Fit Conf %	C	H	N	O	Cl
431.0921	100.00	431.0921	0.0	0.0	0.5	C6 H21 N10 O8 Cl2	476.3	0.136	87.30	6	21	10	8	2
		431.0926	-0.5	-1.2	1.5	C2 H18 N18 O2 Cl3	478.6	2.517	8.07	2	18	18	2	3
		431.0916	0.5	1.2	10.5	C8 H12 N16 O4 Cl	479.4	3.258	3.85	8	12	16	4	1
		431.0929	-0.8	-1.9	12.5	C22 H21 N2 O3 Cl2	481.8	5.727	0.33	22	21	2	3	2
		431.0921	0.0	0.0	8.5	C17 H22 N6 O Cl3	482.4	6.229	0.20	17	22	6	1	3
		431.0929	-0.8	-1.9	4.5	C11 H20 N6 O10 Cl	482.6	6.432	0.16	11	20	6	10	1
		431.0916	0.5	1.2	-0.5	C10 H24 N2 O14 Cl	483.2	7.059	0.09	10	24	2	14	1
		431.0926	-0.5	-1.2	-1.5	C15 H31 O5 Cl4	485.3	9.133	0.01	15	31		5	4
		431.0925	-0.4	-0.9	14.5	C13 H11 N12 O6	489.8	13.693	0.00	13	11	12	6	
		431.0919	0.2	0.5	21.5	C28 H15 O5	491.1	15.003	0.00	28	15		5	

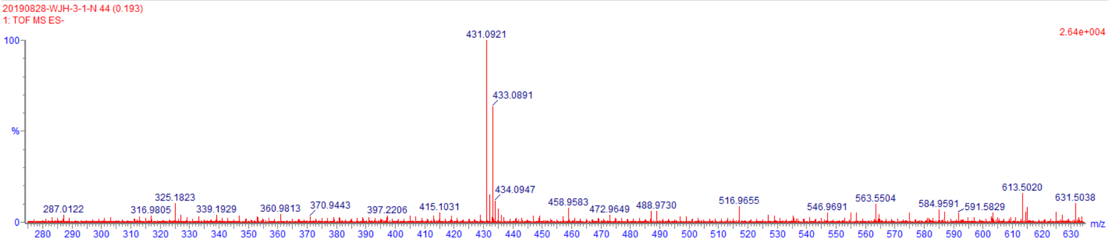

Figure S33. HR-MS of compound **21a**

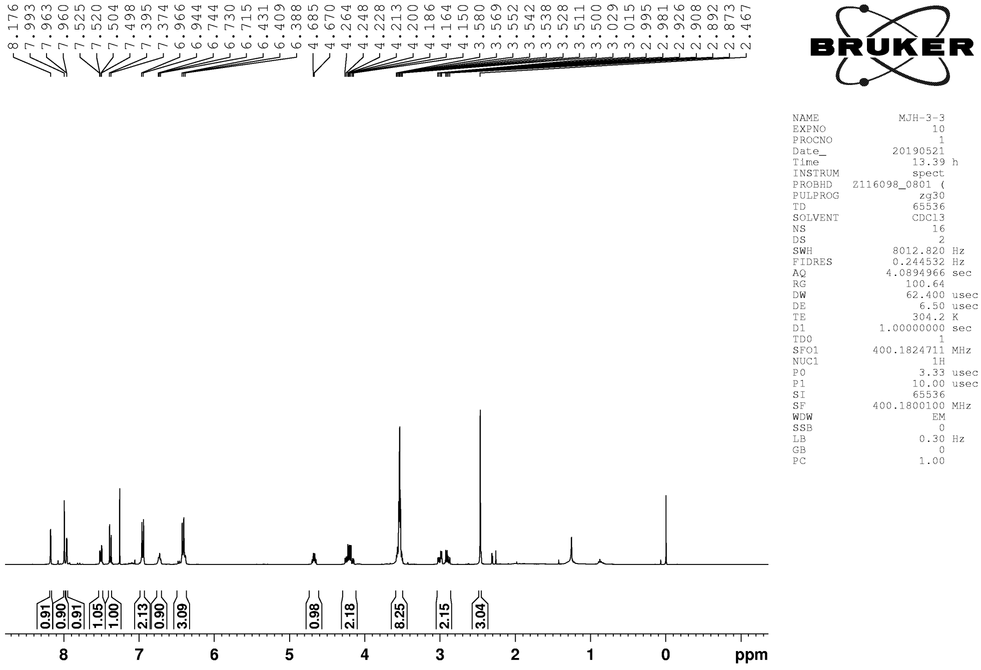

Figure S34. ^{1}H NMR of compound **21b**

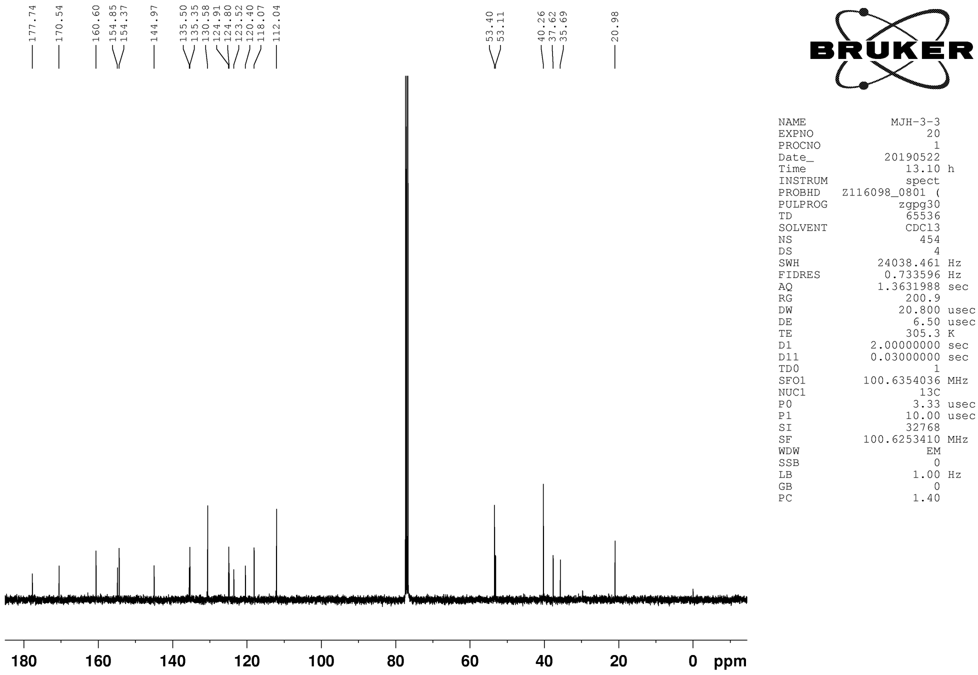

Figure S35. ^{13}C NMR of compound **21b**

Tolerance = 5.0 mDa / DBE: min = -1.5, max = 50.0
Element prediction: Off
Number of isotope peaks used for i-FIT = 3
Monoisotopic Mass, Even Electron Ions
9468 formula(e) evaluated with 76 results within limits (up to 50 closest results for each mass)
Elements Used:
C: 0-50 H: 0-100 N: 0-50 O: 0-100 Cl: 0-10

Mass	RA	Calc. Mass	mDa	PPM	DBE	Formula	i-FIT	i-FIT Norm	Fit Conf %	C	H	N	O	Cl
502.1294	100.00	502.1300	-0.6	-1.2	13.5	C25 H26 N3 O4 Cl2	830.4	0.237	78.87	25	26	3	4	2
		502.1274	2.0	4.0	14.5	C21 H22 N9 O2 Cl2	831.8	1.616	19.87	21	22	9	2	2
		502.1314	-2.0	-4.0	18.5	C26 H22 N7 Cl2	834.6	4.384	1.25	26	22	7		2
		502.1319	-2.5	-5.0	0.5	C13 H30 N5 O11 Cl2	840.2	9.977	0.00	13	30	5	11	2
		502.1292	0.2	0.4	9.5	C20 H27 N7 O2 Cl3	840.7	10.516	0.00	20	27	7	2	3
		502.1319	-2.5	-5.0	11.5	C11 H18 N19 O Cl2	841.6	11.386	0.00	11	18	19	1	2
		502.1278	1.6	3.2	4.5	C19 H31 N3 O6 Cl3	841.6	11.456	0.00	19	31	3	6	3
		502.1287	0.7	1.4	0.5	C13 H29 N3 O15 Cl	841.7	11.502	0.00	13	29	3	15	1
		502.1301	-0.7	-1.4	5.5	C14 H25 N7 O11 Cl	841.9	11.686	0.00	14	25	7	11	1

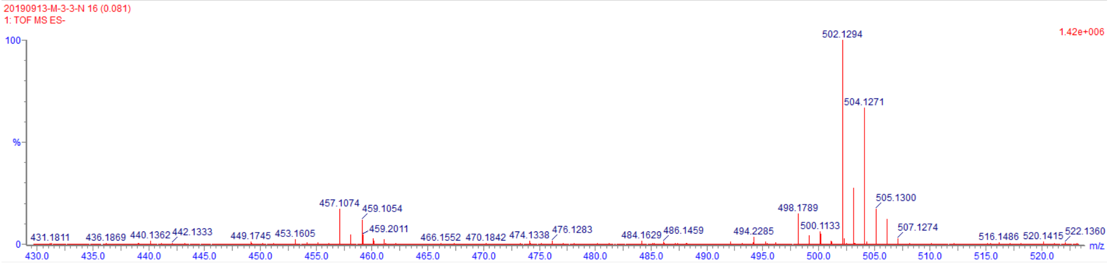

Figure S36. HR-MS of compound **21b**

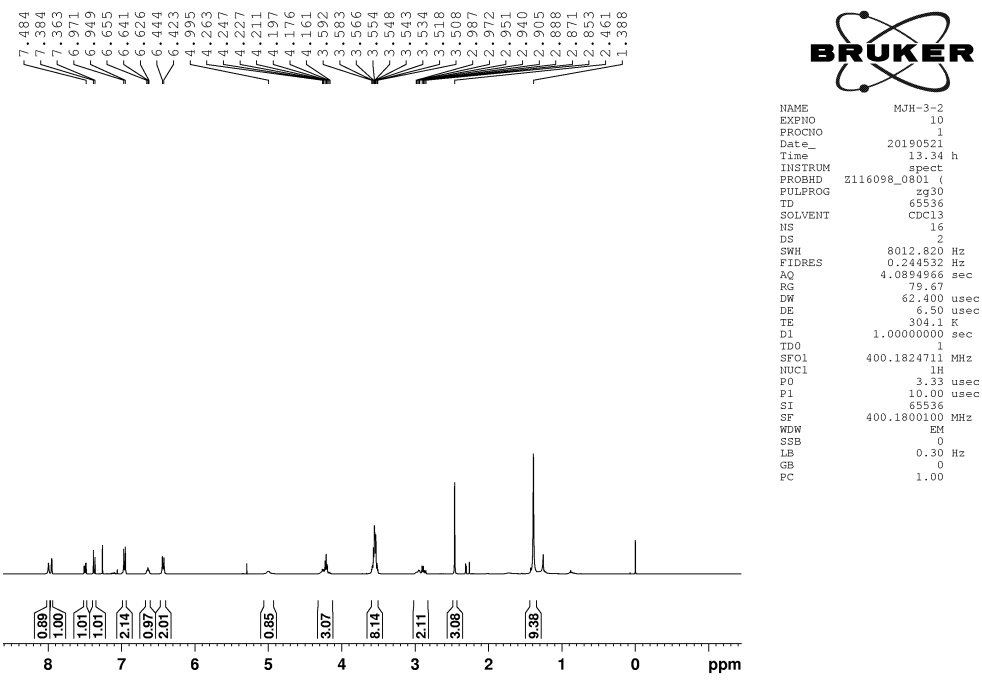

Figure S37. ^{1}H NMR of compound **21c**

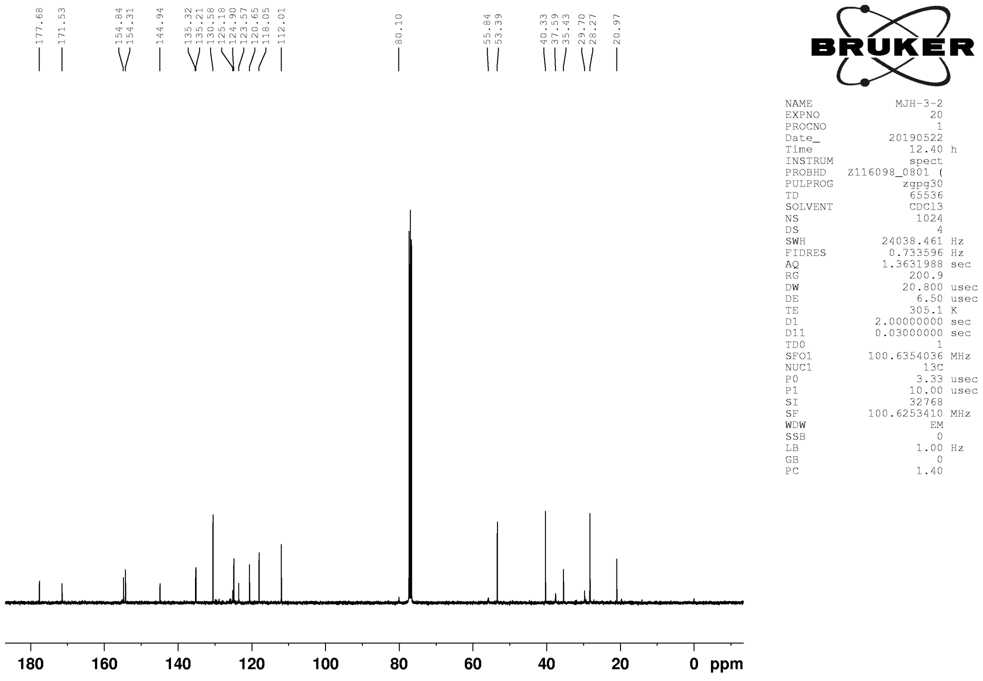

Figure S38. ^{13}C NMR of compound **21c**

Tolerance = 5.0 mDa / DBE: min = -1.5, max = 50.0
Element prediction: Off
Number of isotope peaks used for i-FIT = 3
Monoisotopic Mass, Even Electron Ions
14932 formula(e) evaluated with 94 results within limits (up to 50 closest results for each mass)
Elements Used:
C: 0-50 H: 0-100 N: 0-50 O: 0-100 Cl: 0-10

Mass	RA	Calc. Mass	mDa	PPM	DBE	Formula	i-FIT	i-FIT Norm	Fit Conf %	C	H	N	O	Cl
574.1889	100.00	574.1876	1.3	2.3	13.5	C29 H34 N3 O5 Cl2	584.3	0.338	71.29	29	34	3	5	2
		574.1894	-0.5	-0.9	11.5	C15 H26 N19 O2 Cl2	585.9	1.962	14.05	15	26	19	2	2
		574.1889	0.0	0.0	18.5	C30 H30 N7 O Cl2	586.6	2.677	6.88	30	30	7	1	2
		574.1881	0.8	1.4	6.5	C14 H30 N15 O6 Cl2	587.3	3.387	3.38	14	30	15	6	2
		574.1867	2.2	3.8	12.5	C11 H22 N25 Cl2	588.1	4.115	1.63	11	22	25		2
		574.1907	-1.8	-3.1	5.5	C18 H34 N9 O8 Cl2	588.5	4.539	1.07	18	34	9	8	2
		574.1867	2.2	3.8	1.5	C13 H34 N11 O10 Cl2	589.3	5.316	0.49	13	34	11	10	2
		574.1894	-0.5	-0.9	0.5	C17 H38 N5 O12 Cl2	589.8	5.882	0.28	17	38	5	12	2
		574.1885	0.4	0.7	7.5	C10 H27 N23 Cl3	590.0	6.027	0.24	10	27	23		3
		574.1872	1.7	3.0	2.5	C9 H31 N19 O4 Cl3	590.7	6.774	0.11	9	31	19	4	3

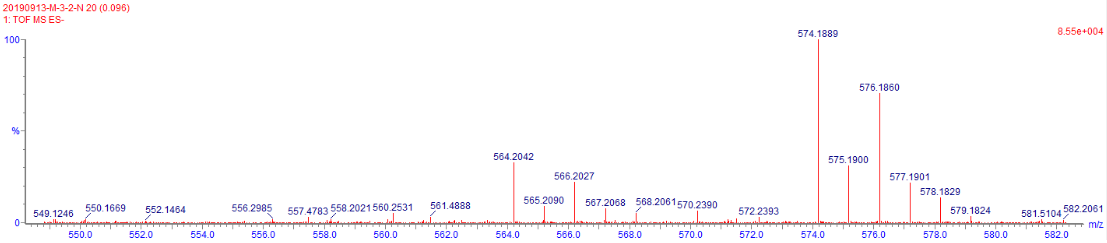

Figure S39. HR-MS of compound **21c**

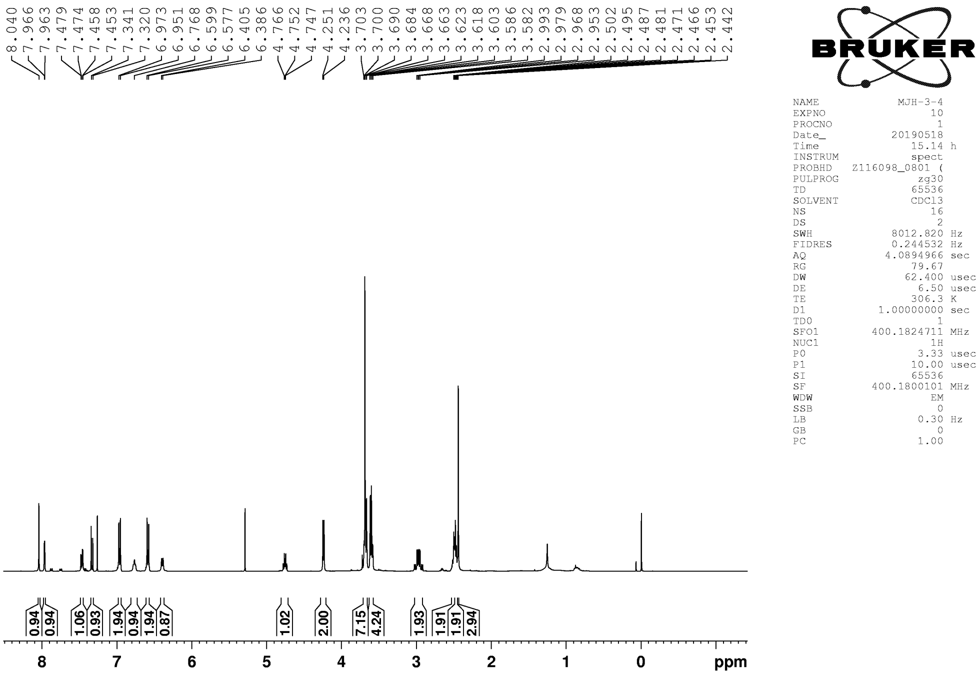

Figure S40. ^{1}H NMR of compound **21d**

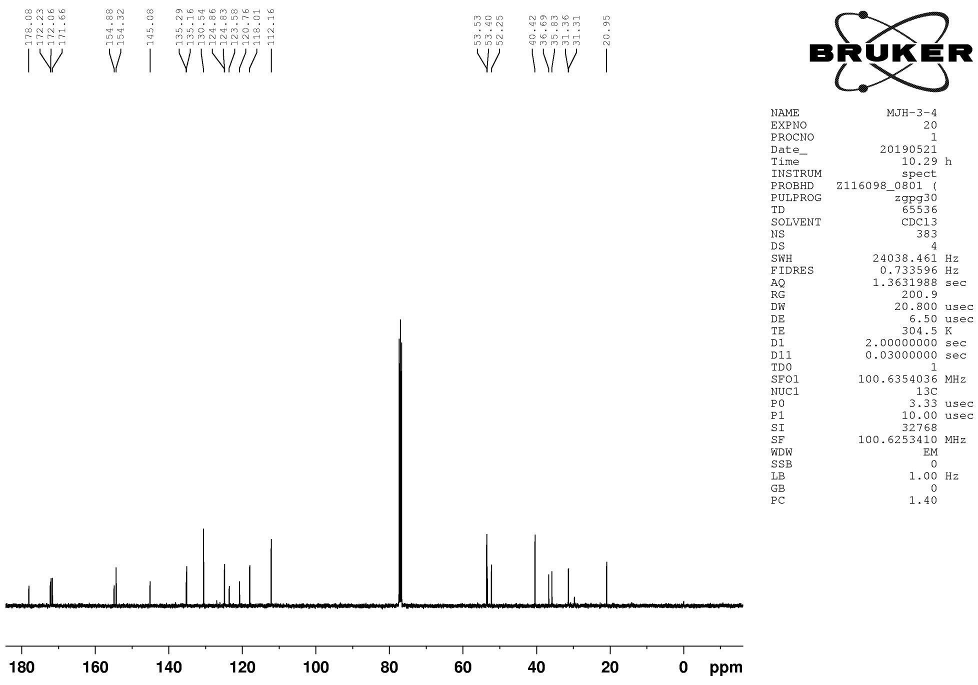

Figure S41. ^{13}C NMR of compound **21d**

Tolerance = 5.0 mDa / DBE: min = -1.5, max = 50.0
Element prediction: Off
Number of isotope peaks used for i-FIT = 3
Monoisotopic Mass, Even Electron Ions
16211 formula(e) evaluated with 117 results within limits (up to 50 closest results for each mass)
Elements Used:
C: 0-50 H: 0-100 N: 0-50 O: 0-100 Cl: 0-10

Mass	RA	Calc. Mass	mDa	PPM	DBE	Formula	i-FIT	i-FIT Norm	Fit Conf %	C	H	N	O	Cl
588.1677	100.00	588.1668	0.9	1.5	14.5	C29 H32 N3 O6 Cl2	730.4	0.122	88.53	29	32	3	6	2
		588.1655	2.2	3.7	20.5	C26 H24 N13 Cl2	732.7	2.368	9.37	26	24	13		2
		588.1682	-0.5	-0.9	19.5	C30 H28 N7 O2 Cl2	734.2	3.898	2.03	30	28	7	2	2
		588.1687	-1.0	-1.7	1.5	C17 H36 N5 O13 Cl2	738.4	8.092	0.03	17	36	5	13	2
		588.1687	-1.0	-1.7	12.5	C15 H24 N19 O3 Cl2	739.4	9.075	0.01	15	24	19	3	2
		588.1673	0.4	0.7	15.5	C25 H29 N11 Cl3	739.9	9.623	0.01	25	29	11		3
		588.1673	0.4	0.7	7.5	C14 H28 N15 O7 Cl2	740.0	9.714	0.01	14	28	15	7	2
		588.1686	-0.9	-1.5	9.5	C28 H37 N O6 Cl3	740.2	9.884	0.01	28	37	1	6	3
		588.1660	1.7	2.9	10.5	C24 H33 N7 O4 Cl3	740.6	10.268	0.00	24	33	7	4	3

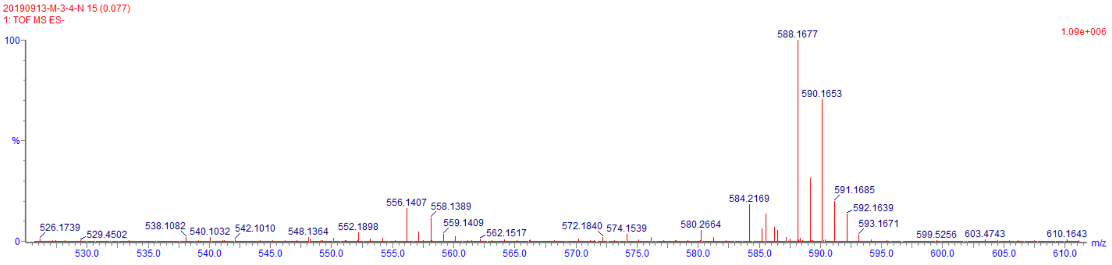

Figure S42. HR-MS of compound **21d**

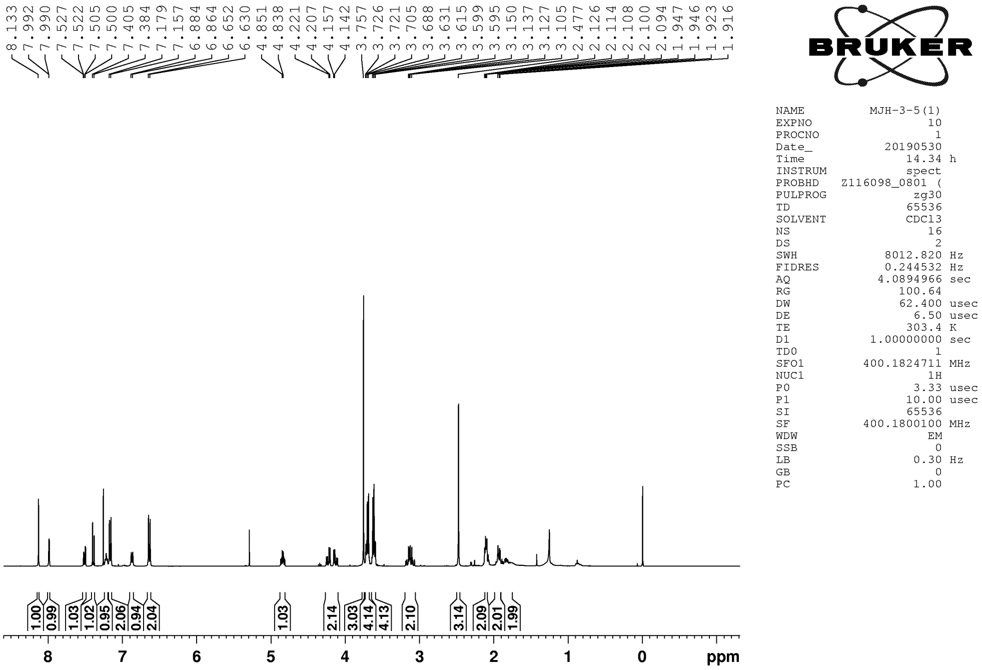

Figure S43. ^{1}H NMR of compound **21e**

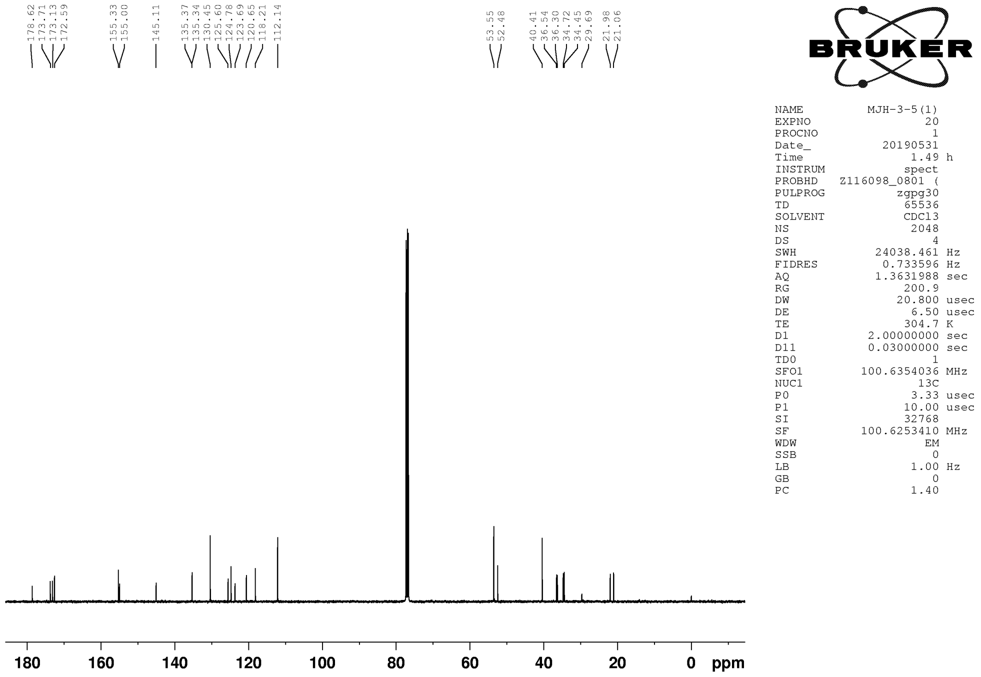

Figure S44. ^{13}C NMR of compound **21e**

Tolerance = 5.0 mDa / DBE: min = -1.5, max = 50.0
Element prediction: Off
Number of isotope peaks used for i-FIT = 3
Monoisotopic Mass, Even Electron Ions
17562 formula(e) evaluated with 116 results within limits (up to 50 closest results for each mass)
Elements Used:
C: 0-50 H: 0-100 N: 0-50 O: 0-100 Cl: 0-10

Mass	RA	Calc. Mass	mDa	PPM	DBE	Formula	i-FIT	i-FIT Norm	Fit Conf %	C	H	N	O	Cl
602.1837	100.00	602.1825	1.2	2.0	14.5	C30 H34 N3 O6 Cl2	689.3	0.015	98.47	30	34	3	6	2
		602.1838	-0.1	-0.2	19.5	C31 H30 N7 O2 Cl2	693.4	4.200	1.50	31	30	7	2	2
		602.1857	-2.0	-3.3	6.5	C19 H34 N9 O9 Cl2	698.4	9.142	0.01	19	34	9	9	2
		602.1843	-0.6	-1.0	12.5	C16 H26 N19 O3 Cl2	698.7	9.506	0.01	16	26	19	3	2
		602.1843	-0.6	-1.0	1.5	C18 H38 N5 O13 Cl2	699.0	9.750	0.01	18	38	5	13	2
		602.1830	0.7	1.2	7.5	C15 H30 N15 O7 Cl2	699.6	10.333	0.00	15	30	15	7	2
		602.1829	0.8	1.3	15.5	C26 H31 N11 Cl3	700.5	11.253	0.00	26	31	11		3
		602.1816	2.1	3.5	2.5	C14 H34 N11 O11 Cl2	700.8	11.532	0.00	14	34	11	11	2
		602.1843	-0.6	-1.0	9.5	C29 H39 N O6 Cl3	701.3	12.020	0.00	29	39	1	6	3

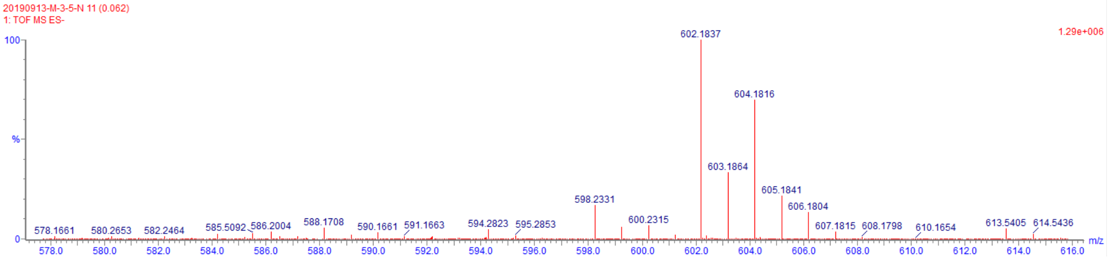

Figure S45. HR-MS of compound **21e**

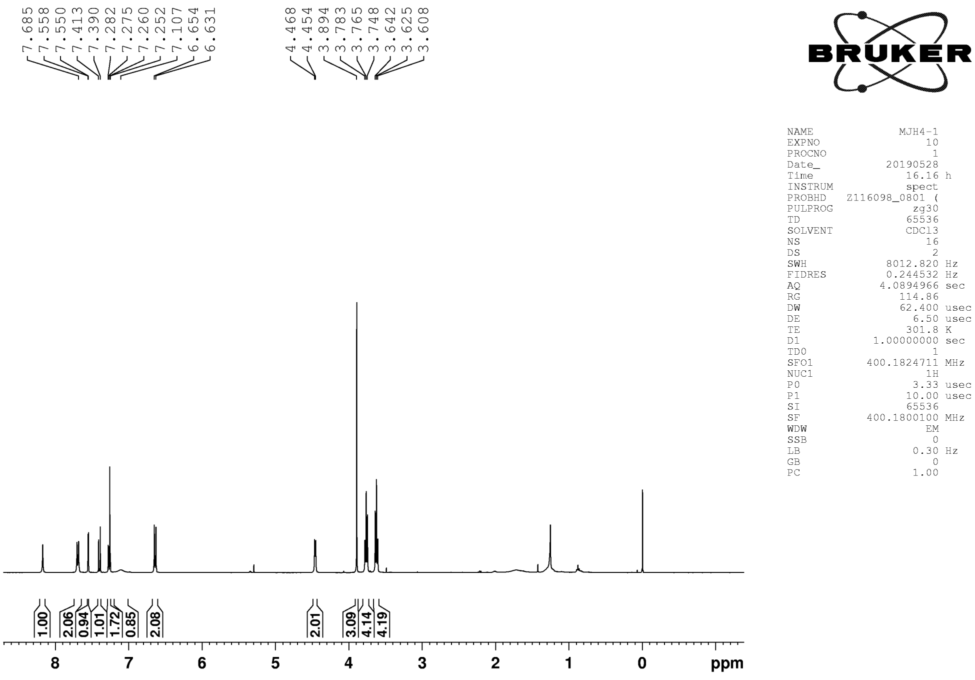

Figure S46. ^{1}H NMR of compound **22a**

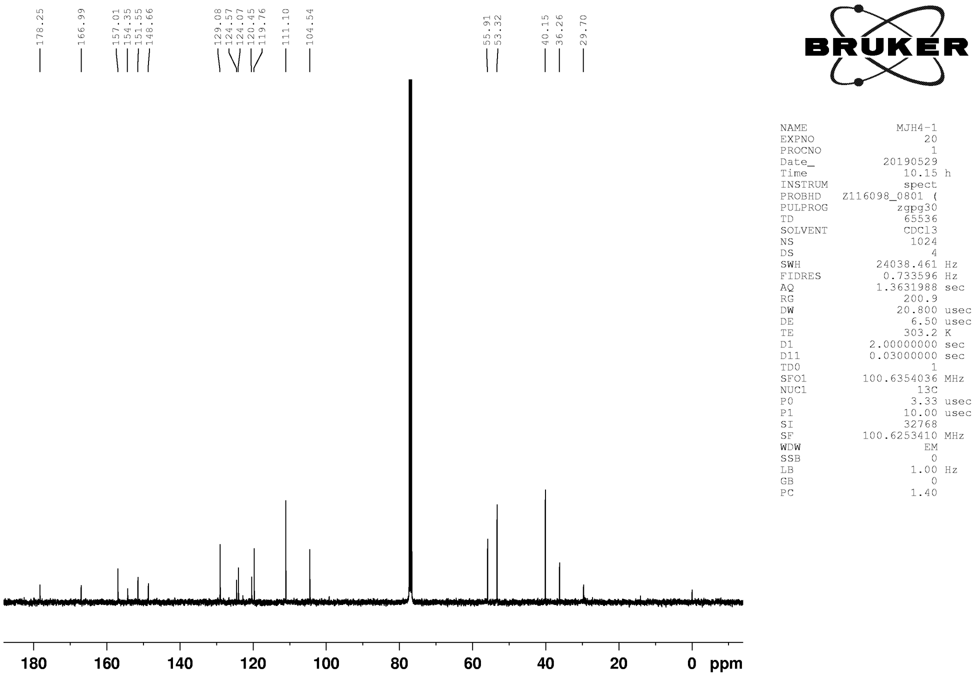

Figure S47. ^{13}C NMR of compound **22a**

Tolerance = 5.0 mDa / DBE: min = -1.5, max = 50.0
Element prediction: Off
Number of isotope peaks used for i-FIT = 3
Monoisotopic Mass, Even Electron Ions
6413 formula(e) evaluated with 68 results within limits (up to 10 closest results for each mass)
Elements Used:
C: 0-50 H: 0-100 N: 0-50 O: 0-100 Cl: 0-10

Mass	RA	Calc. Mass	mDa	PPM	DBE	Formula	i-FIT	i-FIT Norm	Fit Conf %	C	H	N	O	Cl
447.0878	100.00	447.0879	-0.1	-0.2	4.5	C11 H20 N6 O11 Cl	578.9	0.375	68.73	11	20	6	11	1
		447.0878	0.0	0.0	15.5	C9 H8 N20 O Cl	579.7	1.228	29.29	9	8	20	1	1
		447.0874	0.4	0.9	14.5	C13 H11 N12 O7	583.5	4.976	0.69	13	11	12	7	
		447.0882	-0.4	-0.9	26.5	C29 H11 N4 O2	583.8	5.277	0.51	29	11	4	2	
		447.0873	0.5	1.1	22.5	C24 H12 N8 Cl	584.1	5.556	0.39	24	12	8		1
		447.0878	0.0	0.0	12.5	C22 H21 N2 O4 Cl2	584.9	6.408	0.16	22	21	2	4	2
		447.0883	-0.5	-1.1	5.5	C7 H17 N14 O5 Cl2	584.9	6.420	0.16	7	17	14	5	2
		447.0875	0.3	0.7	1.5	C2 H18 N18 O3 Cl3	586.8	8.276	0.03	2	18	18	3	3
		447.0875	0.3	0.7	-1.5	C15 H31 O6 Cl4	586.9	8.431	0.02	15	31		6	4
		447.0880	-0.2	-0.4	-0.5	C11 H28 N8 Cl5	587.4	8.931	0.01	11	28	8		5

20190828-WJH-4-1-N 60 (0.256)
1: TOF MS ES-

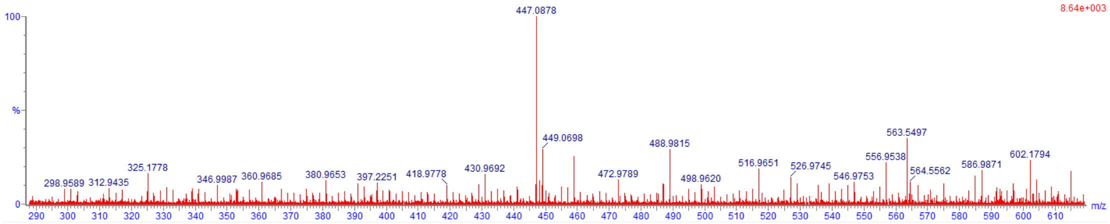

Figure S48. HR-MS of compound **22a**

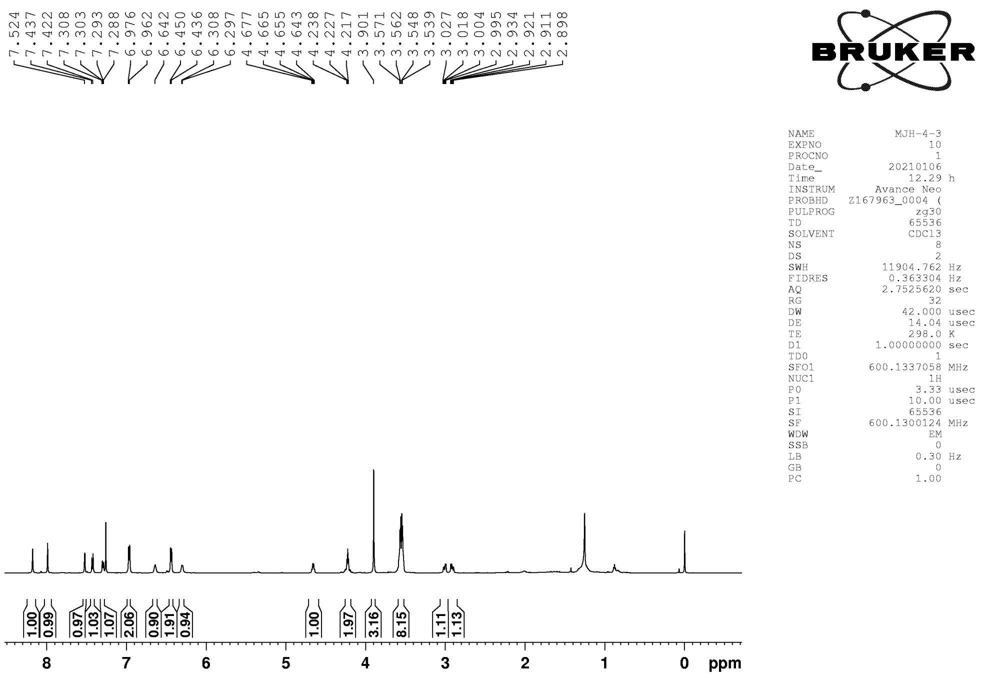

Figure S49. ^{1}H NMR of compound **22b**

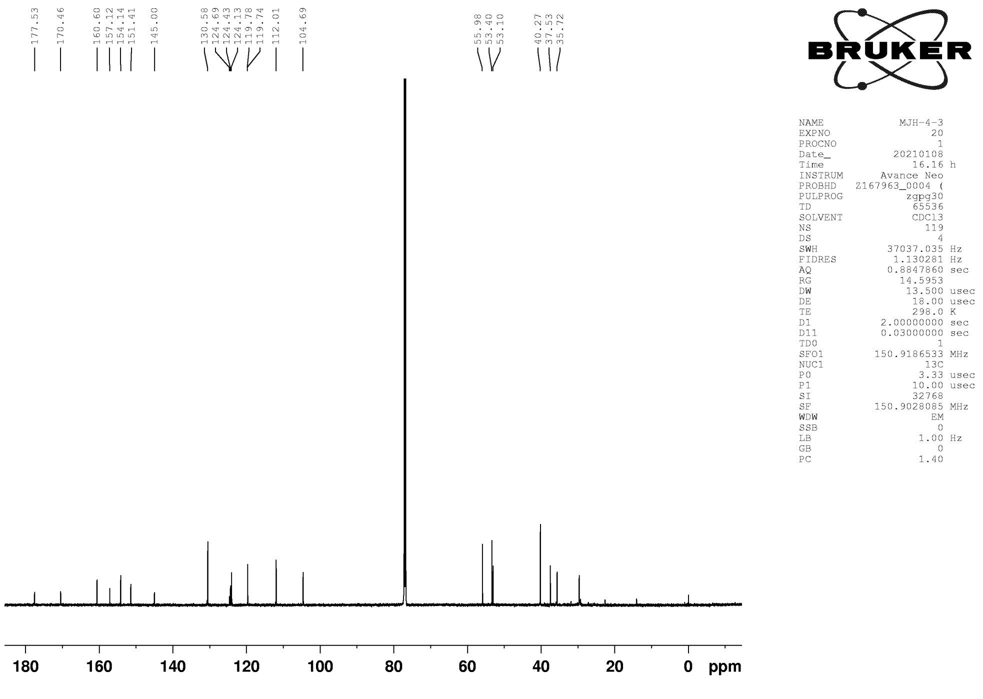

Figure S50. ^{13}C NMR of compound **22b**

Tolerance = 5.0 mDa / DBE: min = -1.5, max = 50.0
Element prediction: Off
Number of isotope peaks used for i-FIT = 3
Monoisotopic Mass, Even Electron Ions
10538 formula(e) evaluated with 87 results within limits (up to 10 closest results for each mass)

Elements Used:
C: 0-50 H: 0-100 N: 0-50 O: 0-100 Cl: 0-10

Mass	RA	Calc. Mass	mDa	PPM	DBE	Formula	i-FIT	i-FIT Norm	Fit Conf %	C	H	N	O	Cl
518.1246	100.00	518.1250	-0.4	-0.8	13.5	C25 H26 N3 O5 Cl2	758.1	0.001	99.93	25	26	3	5	2
		518.1241	0.5	1.0	9.5	C20 H27 N7 O3 Cl3	765.9	7.807	0.04	20	27	7	3	3
		518.1250	-0.4	-0.8	5.5	C14 H25 N7 O12 Cl	767.1	8.969	0.01	14	25	7	12	1
		518.1245	0.1	0.2	23.5	C27 H17 N9 O Cl	767.5	9.422	0.01	27	17	9	1	1
		518.1246	0.0	0.0	-0.5	C18 H36 N O7 Cl4	769.0	10.842	0.00	18	36	1	7	4
		518.1251	-0.5	-1.0	0.5	C14 H33 N9 O Cl5	769.6	11.450	0.00	14	33	9	1	5
		518.1250	-0.4	-0.8	16.5	C12 H13 N21 O2 Cl	770.3	12.189	0.00	12	13	21	2	1
		518.1246	0.0	0.0	2.5	C5 H23 N19 O4 Cl3	772.3	14.213	0.00	5	23	19	4	3
		518.1245	0.1	0.2	15.5	C16 H16 N13 O8	779.0	20.859	0.00	16	16	13	8	
		518.1250	-0.4	-0.8	8.5	C H12 N25 O9	780.1	21.986	0.00	1	12	25	9	

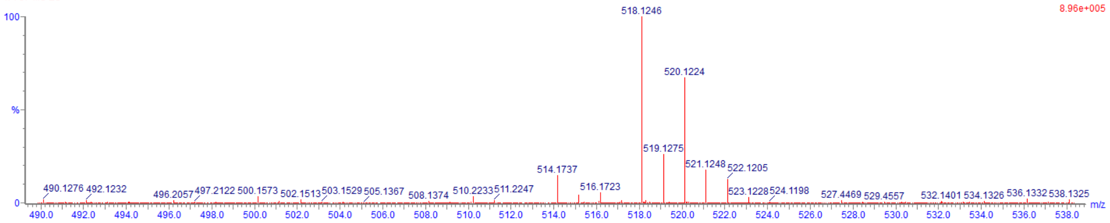

Figure S51. HR-MS of compound **22b**

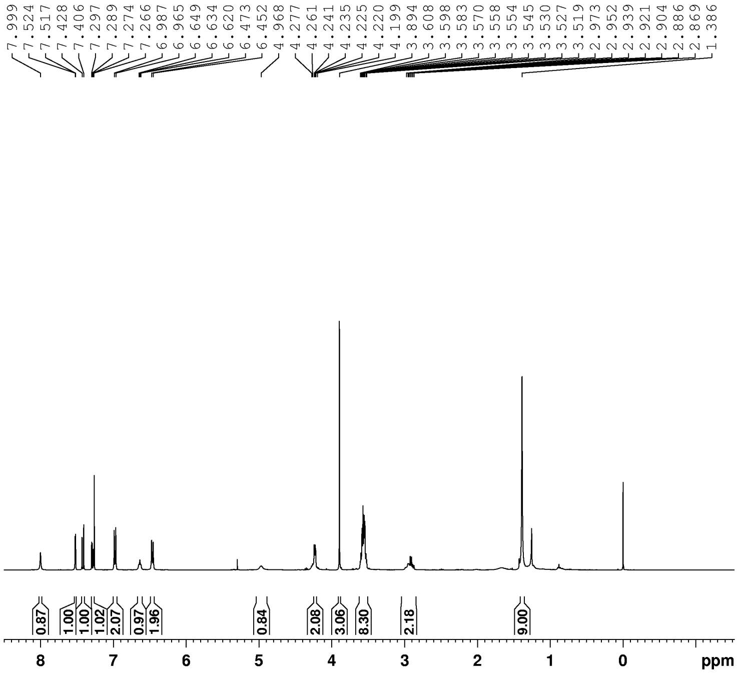

NAME MJH-4-2
EXPNO 10
PROCNO 1
Date_ 20190529
Time 14.04 h
INSTRUM spect
PROBHD Z116098_0801 (
PULPROG zg30
TD 65536
SOLVENT CDCl3
NS 16
DS 2
SWH 8012.820 Hz
FIDRES 0.244532 Hz
AQ 4.0894966 sec
RG 114.86
DW 62.400 usec
DE 6.50 usec
TE 301.5 K
D1 1.00000000 sec
TD0 1
SFO1 400.1824711 MHz
NUC1 1H
P0 3.33 usec
P1 10.00 usec
SI 65536
SF 400.1800100 MHz
WDW EM
SSB 0
LB 0.30 Hz
GB 0
PC 1.00

Figure S52. ^{1}H NMR of compound **22c**

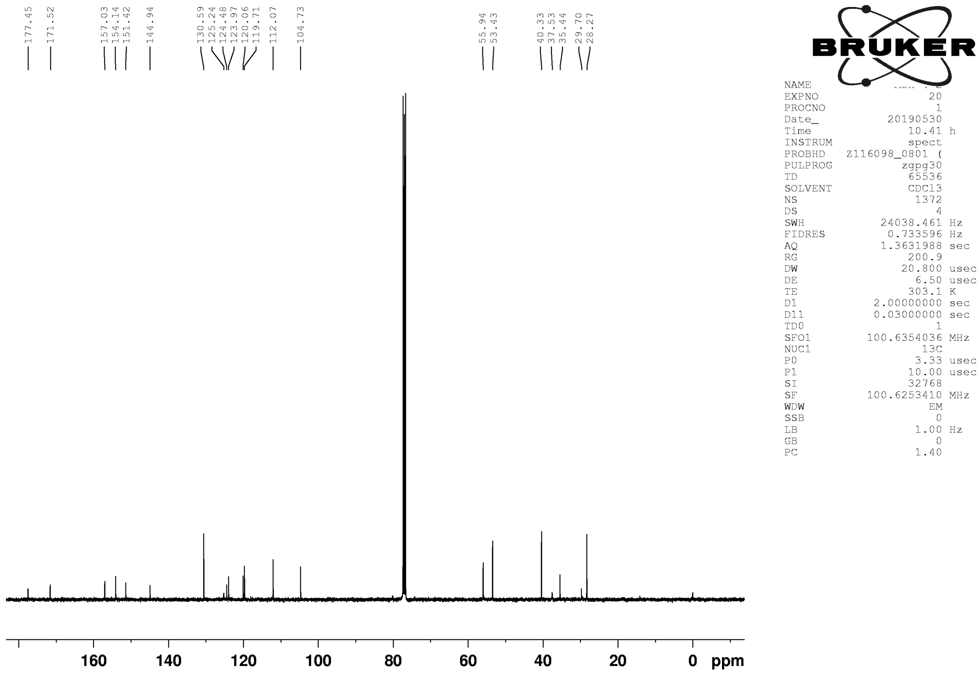

Figure S53. ^{13}C NMR of compound **22c**

Tolerance = 5.0 mDa / DBE: min = -1.5, max = 50.0
Element prediction: Off
Number of isotope peaks used for i-FIT = 3
Monoisotopic Mass, Even Electron Ions
16398 formula(e) evaluated with 108 results within limits (up to 50 closest results for each mass)
Elements Used:
C: 0-50 H: 0-100 N: 0-50 O: 0-100 Cl: 0-10

Mass	RA	Calc. Mass	mDa	PPM	DBE	Formula	i-FIT	i-FIT Norm	Fit Conf %	C	H	N	O	Cl
590.1823	100.00	590.1825	-0.2	-0.3	13.5	C29 H34 N3 O6 Cl2	554.5	0.232	79.28	29	34	3	6	2
		590.1838	-1.5	-2.5	18.5	C30 H30 N7 O2 Cl2	556.6	2.355	9.49	30	30	7	2	2
		590.1811	1.2	2.0	19.5	C26 H26 N13 Cl2	556.8	2.510	8.13	26	26	13		2
		590.1843	-2.0	-3.4	0.5	C17 H38 N5 O13 Cl2	558.9	4.680	0.93	17	38	5	13	2
		590.1843	-2.0	-3.4	8.5	C28 H39 N O6 Cl3	559.4	5.184	0.56	28	39	1	6	3
		590.1829	-0.6	-1.0	14.5	C25 H31 N11 Cl3	559.7	5.426	0.44	25	31	11		3
		590.1816	0.7	1.2	9.5	C24 H35 N7 O4 Cl3	559.9	5.650	0.35	24	35	7	4	3
		590.1843	-2.0	-3.4	11.5	C15 H26 N19 O3 Cl2	560.4	6.128	0.22	15	26	19	3	2
		590.1803	2.0	3.4	4.5	C23 H39 N3 O8 Cl3	560.6	6.399	0.17	23	39	3	8	3
		590.1830	-0.7	-1.2	6.5	C14 H30 N15 O7 Cl2	560.8	6.570	0.14	14	30	15	7	2

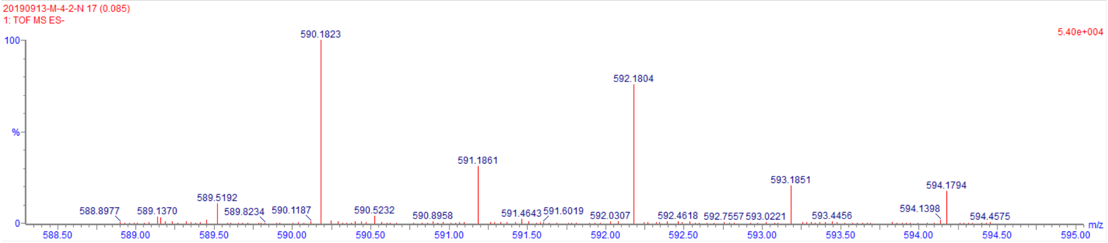

Figure S54. HR-MS of compound **22c**

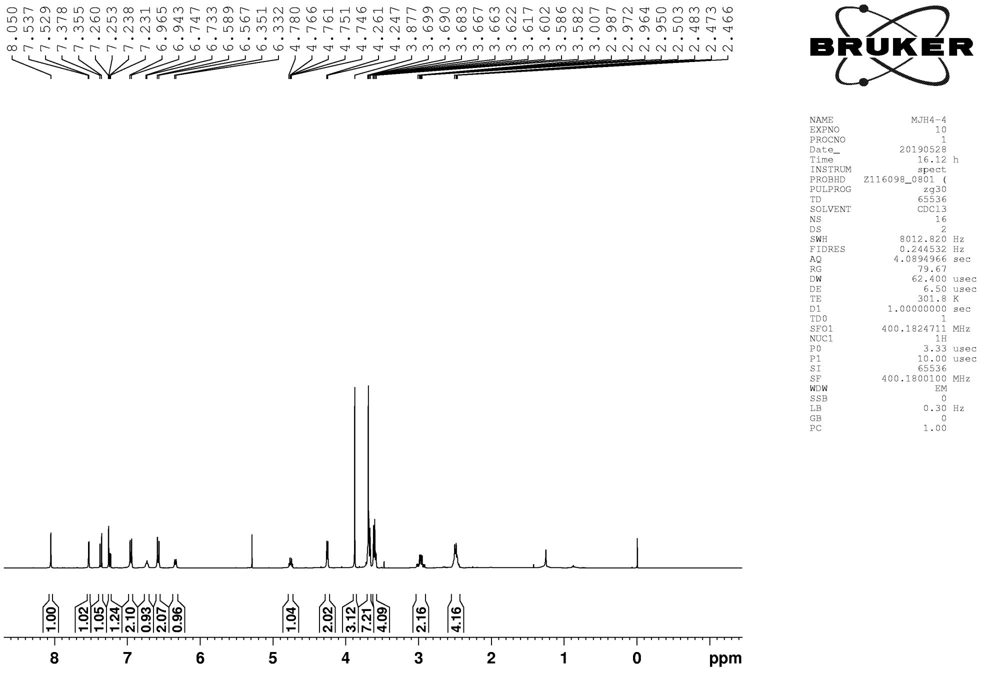

Figure S55. ^{1}H NMR of compound **22d**

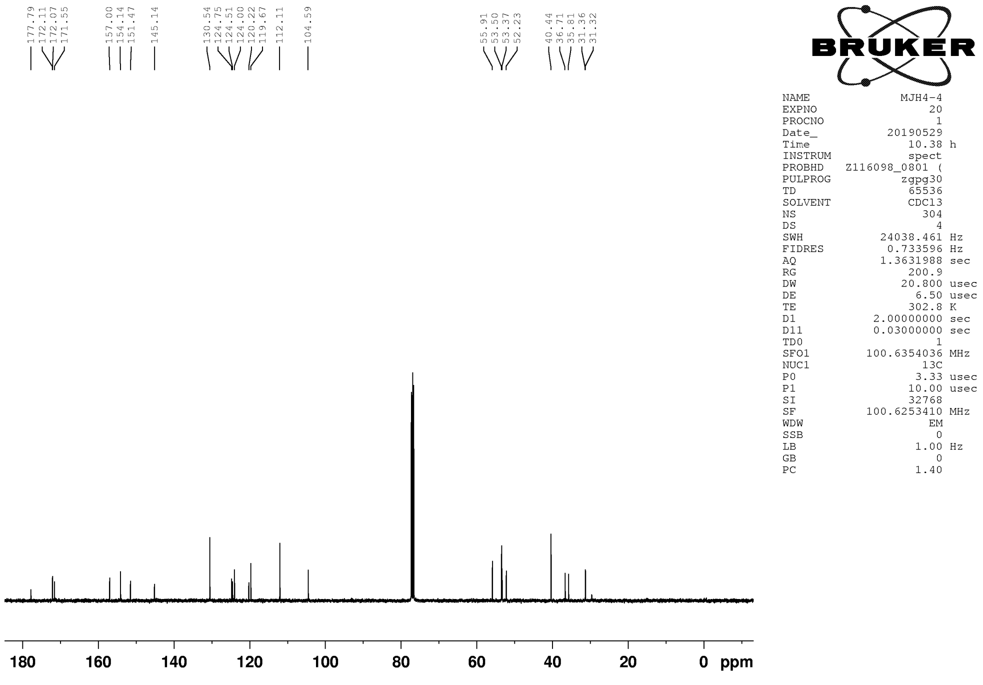

Figure S56. ^{13}C NMR of compound **22d**

Tolerance = 5.0 mDa / DBE: min = -1.5, max = 50.0
Element prediction: Off
Number of isotope peaks used for i-FIT = 3
Monoisotopic Mass, Even Electron Ions
17763 formula(e) evaluated with 128 results within limits (up to 50 closest results for each mass)
Elements Used:
C: 0-50 H: 0-100 N: 0-50 O: 0-100 Cl: 0-10

Mass	RA	Calc. Mass	mDa	PPM	DBE	Formula	i-FIT	i-FIT Norm	Fit Conf %	C	H	N	O	Cl
604.1627	100.00	604.1617	1.0	1.7	14.5	C29 H32 N3 O7 Cl2	649.0	0.065	93.71	29	32	3	7	2
		604.1631	-0.4	-0.7	19.5	C30 H28 N7 O3 Cl2	651.8	2.847	5.80	30	28	7	3	2
		604.1636	-0.9	-1.5	1.5	C17 H36 N5 O14 Cl2	654.9	6.030	0.24	17	36	5	14	2
		604.1636	-0.9	-1.5	12.5	C15 H24 N19 O4 Cl2	656.2	7.254	0.07	15	24	19	4	2
		604.1636	-0.9	-1.5	9.5	C28 H37 N O7 Cl3	656.6	7.665	0.05	28	37	1	7	3
		604.1622	0.5	0.8	15.5	C25 H29 N11 O Cl3	656.9	8.009	0.03	25	29	11	1	3
		604.1622	0.5	0.8	7.5	C14 H28 N15 O8 Cl2	657.0	8.057	0.03	14	28	15	8	2
		604.1609	1.8	3.0	10.5	C24 H33 N7 O5 Cl3	657.6	8.648	0.02	24	33	7	5	3
		604.1609	1.8	3.0	2.5	C13 H32 N11 O12 Cl2	657.8	8.919	0.01	13	32	11	12	2

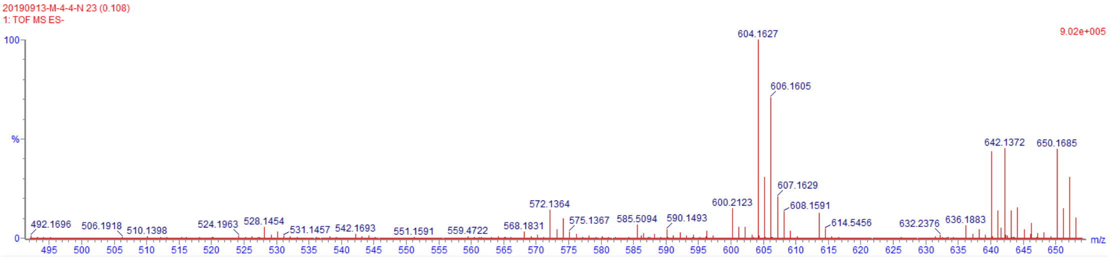

Figure S57. HR-MS of compound **22d**

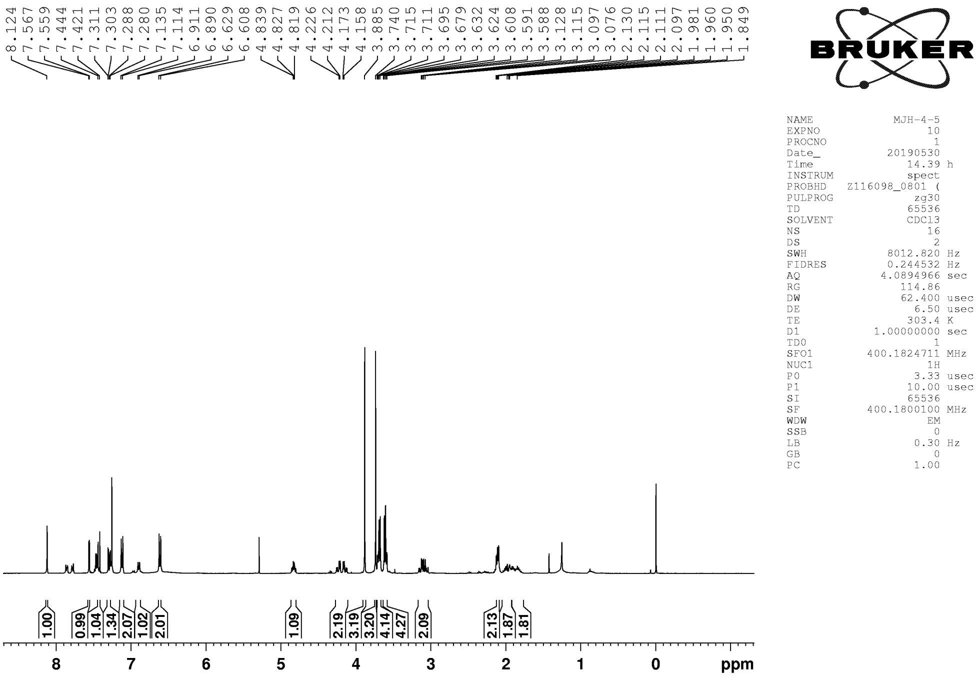

Figure S58. ^{1}H NMR of compound **22e**

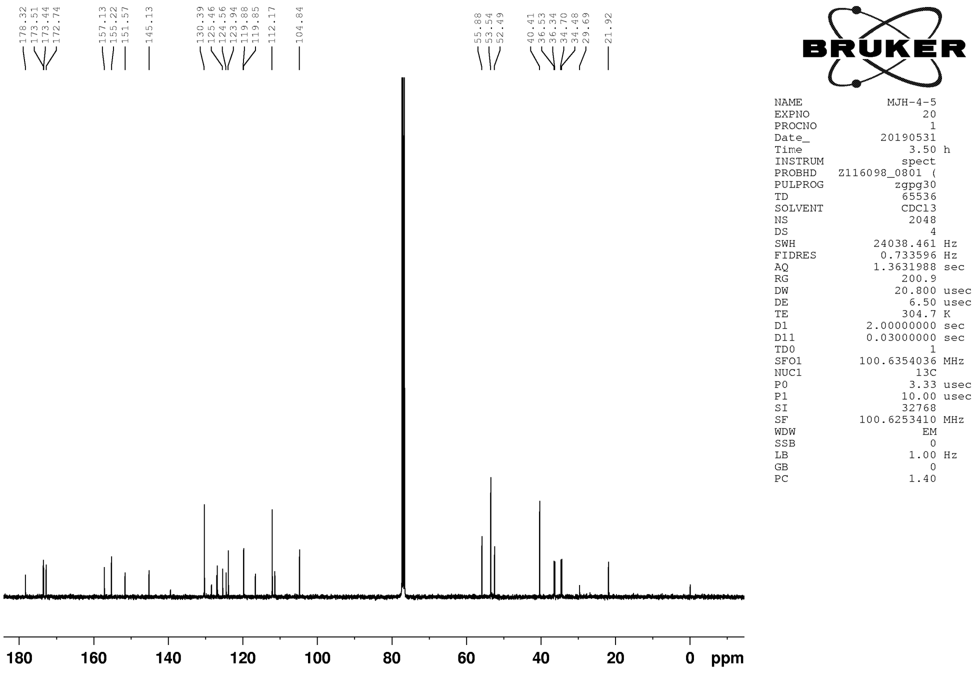

Figure S59. ^{13}C NMR of compound **22e**

Tolerance = 5.0 mDa / DBE: min = -1.5, max = 50.0
Element prediction: Off
Number of isotope peaks used for i-FIT = 3
Monoisotopic Mass, Even Electron Ions
19200 formula(e) evaluated with 129 results within limits (up to 10 closest results for each mass)
Elements Used:
C: 0-50 H: 0-100 N: 0-50 O: 0-100 Cl: 0-10

Mass	RA	Calc. Mass	mDa	PPM	DBE	Formula	i-FIT	i-FIT Norm	Fit Conf %	C	H	N	O	Cl
618.1776	100.00	618.1774	0.2	0.3	14.5	C30 H34 N3 O7 Cl2	672.9	0.000	100.00	30	34	3	7	2
		618.1779	-0.3	-0.5	15.5	C26 H31 N11 O Cl3	683.5	10.646	0.00	26	31	11	1	3
		618.1774	0.2	0.3	6.5	C19 H33 N7 O14 Cl	685.3	12.438	0.00	19	33	7	14	1
		618.1775	0.1	0.2	1.5	C19 H41 N9 O3 Cl5	687.2	14.335	0.00	19	41	9	3	5
		618.1774	0.2	0.3	17.5	C17 H21 N21 O4 Cl	687.6	14.682	0.00	17	21	21	4	1
		618.1775	0.1	0.2	4.5	C6 H28 N27 Cl4	691.6	18.749	0.00	6	28	27		4
		618.1774	0.2	0.3	20.5	C4 H8 N39 O	695.1	22.180	0.00	4	8	39	1	
		618.1774	0.2	0.3	9.5	C6 H20 N25 O11	696.6	23.704	0.00	6	20	25	11	
		618.1774	0.2	0.3	-1.5	C8 H32 N11 O21	696.6	23.730	0.00	8	32	11	21	
		618.1777	-0.1	-0.2	28.5	C37 H24 N5 O5	696.7	23.775	0.00	37	24	5	5	

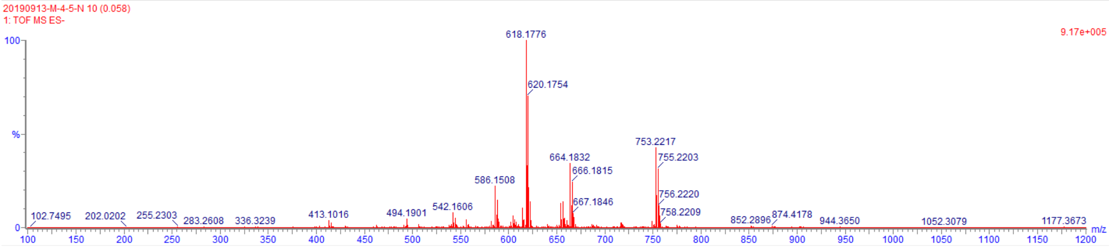

Figure S60. HR-MS of compound **22e**
